# Ribosomal protein mRNAs are translationally-regulated during human dendritic cells activation by LPS

**DOI:** 10.1186/1745-7580-5-5

**Published:** 2009-11-27

**Authors:** Maurizio Ceppi, Giovanna Clavarino, Evelina Gatti, Enrico K Schmidt, Aude de Gassart, Derek Blankenship, Gerald Ogola, Jacques Banchereau, Damien Chaussabel, Philippe Pierre

**Affiliations:** 1 Centre d'Immunologie de Marseille-Luminy, Université de la Méditerranée, Case 906, 13288 Marseille cedex 9, France; 2 INSERM, U631, 13288 Marseille, France; 3 CNRS, UMR6102, 13288 Marseille, France; 4 Baylor Institute for Immunology Research (BIIR), 3434 Live Oak, Dallas, TX 75204, USA; 5 Baylor Institute for Health Care Research and Improvement, 8080 North Central Expressway, Dallas, TX 75206, USA; 6 Genomic Vision, Paris Santé Cochin, 75014 Paris, France

## Abstract

**Background:**

Dendritic cells (DCs) are the sentinels of the mammalian immune system, characterized by a complex maturation process driven by pathogen detection. Although multiple studies have described the analysis of activated DCs by transcriptional profiling, recent findings indicate that mRNAs are also regulated at the translational level. A systematic analysis of the mRNAs being translationally regulated at various stages of DC activation was performed using translational profiling, which combines sucrose gradient fractionation of polysomal-bound mRNAs with DNA microarray analysis.

**Results:**

Total and polysomal-bound mRNA populations purified from immature, 4 h and 16 h LPS-stimulated human monocyte-derived DCs were analyzed on Affymetrix microarrays U133 2.0. A group of 375 transcripts was identified as translationally regulated during DC-activation. In addition to several biochemical pathways related to immunity, the most statistically relevant biological function identified among the translationally regulated mRNAs was protein biosynthesis itself. We singled-out a cluster of 11 large ribosome proteins mRNAs, which are disengaged from polysomes at late time of maturation, suggesting the existence of a negative feedback loop regulating translation in DCs and linking ribosomal proteins to immuno-modulatory function.

**Conclusion:**

Our observations highlight the importance of translation regulation during the immune response, and may favor the identification of novel protein networks relevant for immunity. Our study also provides information on the potential absence of correlation between gene expression and protein production for specific mRNA molecules present in DCs.

## Background

Dendritic cells (DCs) are haematopoietic cells specialized in antigen capture and presentation for initiation of primary and secondary immune responses. Due to this central role in induction and regulation of immunity, they represent an attractive target for immunotherapy against various diseases, including cancer and microbial infections [1]. We recently demonstrated that translation regulation is required for function and survival of mouse activated DCs [2]. Moreover, emerging evidence indicate that translation plays a major role in immune regulation and its dysfunction can lead to pathology [3-5]. Although several seminal studies have described the use of microarrays to define the gene expression and functional signature of DCs upon pathogen detection [6,7], there were no attempts to include the additional layer of complexity brought by translational regulation. As the relationship between inflammation, innate immunity, and post-transcriptional regulation is becoming clearer [8], we have in a recent study used a microarray-based screen to identify the immunologically relevant pathways regulated by miR-155 in lipopolysaccharide (LPS)-activated human monocyte-derived DC (moDC) [9]. To increase further our understanding of post-transcriptional regulation and establish the contribution of translation in the control of immune response, we carried-out, using Affymetrix microarrays, a systematic and comparative analysis of polysome-bound mRNA [10-12] purified from differently LPS-activated moDCs.

Using this approach, and in addition to several immunologically relevant mRNAs, we identified a network of ribosomal protein mRNAs being strongly down-modulated at the translational level at late time of DC maturation. Ribosomal proteins are integral components of the basal cellular machinery involved in protein synthesis, whose roles have been regarded collectively as important, but individually disregarded. Recent findings, however, have demonstrated that components of the translational apparatus are multifunctional and that several individual ribosomal proteins play a role in regulating cell growth, transformation and death [13]. Our results clearly support these views and underline the importance of these proteins for DC function.

## Results and discussion

### Translation is regulated in LPS-activated human moDCs

**Figure 1:**
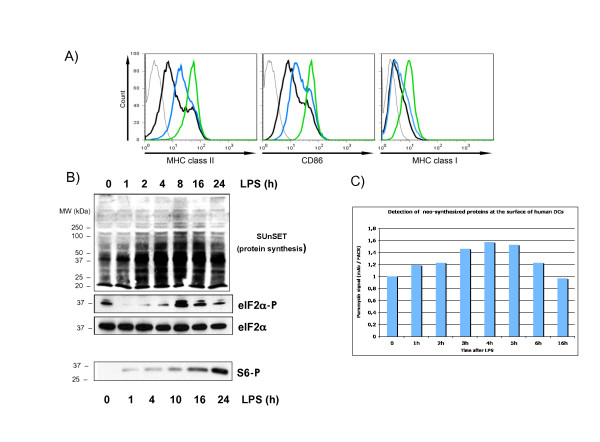
**Translation is regulated in LPS-activated moDCs**. moDCs were stimulated with LPS for the indicated timepoints and after harvesting both the maturation status and protein synthesis were monitored in parallel. (A) The surface accumulation of MHC II, CD86 and MHC I, were measured by flow cytometry in immature (black line), 4 h (blue) or 16 h (green) LPS-stimulated moDCs. (B) The rate of protein synthesis was monitored with puromycin incorporation using immunoblots (top). Cells extracts were separated by denaturing electrophoresis and analyzed by western blot with antibody to the phosphorylated form of eIF2α and the S6 ribosomal protein (bottom). Total eIF2α detection is shown for equal loading control. (C) FACS analysis with antibody to puromycin (SUnSET method) to quantify protein synthesis. Data are representative of at least three independent experiments, each derived from a different DC preparation.

Human monocyte-derived DCs were activated with LPS and displayed the expected cell surface accumulation of MHC I, MHC II and CD86 as measured by flow cytometry (Figure 1A). The rate of protein synthesis in activated moDCs was monitored with puromycin incorporation using immunoblot or FACS analysis (SUnSET) [14]. Protein synthesis intensity was strongly increased upon LPS-stimulation and peaked at 4 h of activation, prior a steady decrease to basal levels (Figure 1B and 1C). As the rate-limiting step in protein synthesis is the initiation of mRNA translation, we investigated the status of translation factors involved in the regulation of protein synthesis. Thus, we monitored by immunoblot the phosphorylation of the alpha-subunit of eukaryotic translation initiation factor 2 (eIF2α), which prevents the assembly of ribosomal pre-initiation complexes [15]. Almost complete eIF2α dephosphorylation was detected between 1 h and 4 h after LPS-stimulation, correlating with the increase in protein synthesis. Interestingly, after several hours of LPS-stimulation eIF2α phosphorylation was recovered and even increased compared to the immature DC levels. Thus eIF2α phosphorylation is regulated during the maturation of human moDCs and correlates well with the regulation of mRNA translation in these cells.

Translation initiation is also controlled through the stimulation of the PI3 kinase/AKT/mTOR signal transduction cascade, which leads to the phosphorylation of eIF4E-binding proteins (4E-BPs) and of the S6 ribosomal protein (S6) by the ribosomal protein S6 kinase (S6K1) [16]. Both molecules have an important role in regulating cap-mediated translation and S6 phosphorylation represents a hallmark of protein synthesis activation. S6 phosphorylation was monitored in LPS-activated moDCs by immunoblot (Figure 1B). We confirmed that S6 phosphorylation is steadily and strongly increased over time of LPS-exposure and this in a correlated manner to the moDCs maturation phenotype.

Thus, protein synthesis is tightly controlled upon LPS-sensing by MoDCs and is likely to represent a functionally important aspect of human DC maturation. Furthermore, this process implies that specific mRNA molecules might be translationally regulated in response to eIF2α or S6 phosphorylation under these conditions.

### mRNA translational engagement changes at late time points of DC-activation

**Figure 2:**
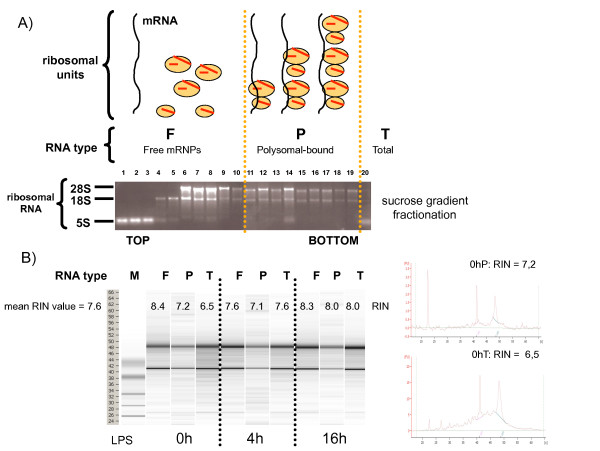
**Efficient profiling and isolation of polysomes out of human moDCs**. Polysomes sedimentation profiles (A) and RNA-integrity profiles (B) after sucrose gradient fractionation of untreated (0 h) or 4 h and 16 h LPS-stimulated moDCs.

In order to identify mRNA molecules potentially regulated at the translational level, the global engagement of mRNA molecules on polysomes was measured on LPS-activated moDCs by translational profiling [10-12]. Actively translated polysome-bound mRNAs (P) were separated from messenger free ribonucleoproteins (mRNPs, F) using sucrose gradient fractionation and visualized by denaturing agarose gel (Figure 2A). Efficient polysome separation was confirmed by the presence of 5S rRNA in the lighter fractions of the gradient (lanes 1-3), followed by fractions where only 18S rRNA was visible (lanes 4-5) and fractions in which an excess of large ribosomal subunit was detected (28S rRNA >> 18S rRNA, lanes 6-9). From fraction 11, a constant ratio of 28S over 18S rRNA was observed, thus indicating the presence of complete and functional ribosomes (Polysomes, lanes 11-19). Fractions 11 to 19 were pooled and polysome-bound RNAs were isolated (P). In parallel, total RNAs (T) were directly purified from moDCs without fractionation (lane 20). The integrity of purified RNAs was quantified to an average RIN-value of 7.6, thereby allowing for successful microarrays analysis (Figure 2B) [17].

In addition to non-activated cells (0 h), moDCs at 4 h and 16 h post-LPS stimulation were also chosen, since they represent two distinct activation states in which protein synthesis is either increasing (4 h), or decreasing (16 h), and in which the phosphorylation status of eIF2-α is radically different. No major difference in the polysomes sedimentation profiles was observed after the fractionation of the various DCs samples, confirming that actively translated mRNAs are present at all stages of DC maturation (not shown). To further identify translationally regulated mRNAs in maturing moDC, expression levels of all polysome-bound mRNAs were compared by microarray analysis (Affymetrix U133 Plus 2.0 GeneChip array comprising 54'675 probe sets) to those of unfractionated total mRNAs (translational profiling, Figure 2). Polysome-bound mRNAs (P) and total RNA (T) were isolated from moDCs generated from four different blood donors for three LPS activation time points (0 h, 4 h and 16 h), resulting in a total of twenty-four RNA samples to be analyzed. In addition to FACS characterization of the cells, the quality of the samples was further evaluated by comparing the total mRNA expression values of several DC-maturation markers from our experimental setting with available public databases values obtained under similar conditions [6]. Transcription of the co-stimulatory molecules CD80 and CD86, as well as the MHC I and II mRNAs were found to be all up-regulated after DC stimulation by LPS (see Additional file 1), thus confirming the quality of our samples and reliability of our analysis.

**Figure 3:**
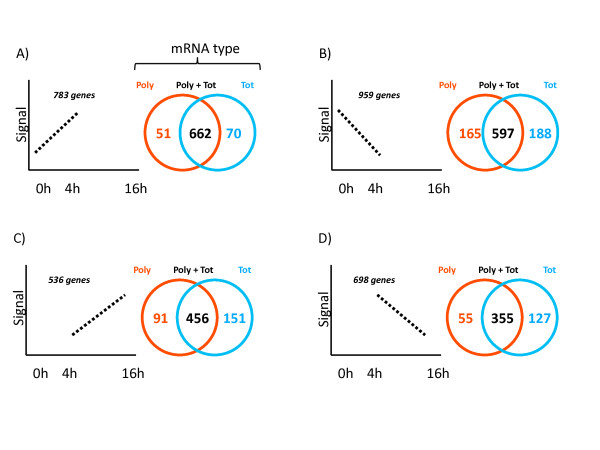
**Global alterations of total and polysomal-bound mRNA in LPS-activated moDCs**. The 54'675 probe sets present on the Affymetrix U133 Plus 2.0 GeneChip array were first filtered on expression (signal > 100 in all tested conditions) to obtain a preliminary list of 7'709 probe sets. The 7'709 probe sets were then filtered on fold change (applying a 2-fold cut-off) comparing Polysomal (Poly, red) and Total (Tot, blue) mRNA, between 0 h and 4 h (A and B) or 4 h and 16 h (C and D) post-LPS. For effective Venn diagram visualization, the transcriptionally up-regulated probe sets (A and C) were distinguished from the transcriptionally down-regulated probe sets (B and D). See Additional file 3 for a detailed description of the different genes subsets.

Global alterations of total and polysomal-bound mRNA as a function of time post-LPS was investigated, to obtain a comprehensive view of translation regulation in moDCs. The 54'675 probe sets present were first filtered on expression (signal > 100 in all tested conditions) to obtain a preliminary list of 7'709 probe sets. The 7'709 probe sets were then selected on fold change (applying a 2-fold cut-off), comparing Polysomal and Total mRNA at different timepoints. Between 0 h and 4 h post-LPS, among the 783 transcriptionally up-regulated genes (signal 0 h < signal 4 h), 662 genes (84%) were shared between total and polysomal RNA, 51 genes (6%) were unique to polysomal RNA, and 70 genes (10%) were unique to total RNA (Figure 3A). Among the 959 transcriptionally down-regulated genes (signal 0 h > signal 4 h), 597 genes (63%) were shared between total and polysomal RNA, 165 genes (17%) were unique to polysomal RNA, and 188 genes (20%) were unique to total RNA (Figure 3B). Between 4 h and 16 h post-LPS, among the 536 transcriptionally up-regulated genes (signal 4 h < signal 16 h), 456 genes (65%) were shared between total and polysomal RNA, 91 genes (13%) were unique to polysomal RNA, and 151 genes (22%) were unique to total RNA (Figure 3C). Similarly, among the 698 transcriptionally down-regulated genes (signal 4 h > signal 16 h), 355 genes (66%) were shared between total and polysomal RNA, 55 genes (10%) were unique to polysomal RNA, and 127 genes (24%) were unique to total RNA (Figure 3D). These results indicate that during DC maturation, transcription and translation intensity are relatively well coupled upon LPS-sensing, as suggested by the overall increase in protein synthesis at the onset of maturation. However, we evaluate to 30% (the mean percentage of the genes unique to polysomal RNA and total RNA) the proportion of translationally-engaged mRNA molecules, in which transcription and translation are not linearly connected. The polysome-bound (that is, translated) mRNAs were up-regulated and down-regulated with the same proportions indicating that translation regulation in DCs is probably targeting discrete subsets of genes, whereas the majority of genes are regulated by transcription and mRNA stability, a detailed description of these genes subsets is given in Additional file 2. Most of the identified genes were distributed in all biological activities with no clear functional clustering capable of unraveling a distinct pattern of regulation. However, an over-representation of protein synthesis genes was found in the genes identified as translationaly down-regulated at late time of maturation (see Additional file 2D).

### Biological functions of translationally regulated mRNAs in activated moDCs

We decided to take a statistically unbiased approach to further identify entire functional pathways, which could be regulated at the translational level. Importantly, the data obtained from four different donors were homogenous and no major pattern variation for both total and polysomal-bound RNA expression was found, thus allowing statistical analysis (see Additional file 3, compare the four columns within each T or P).

**Figure 4:**
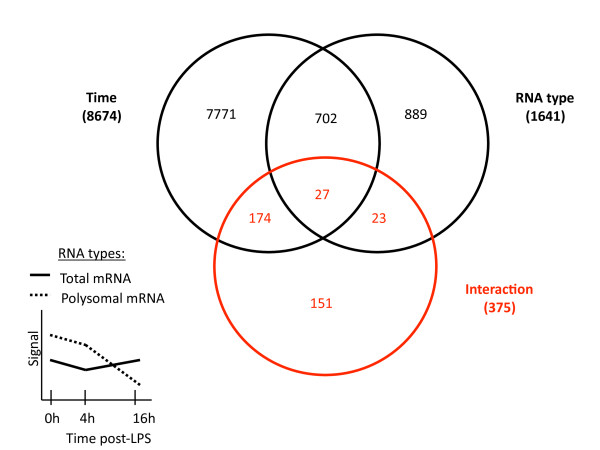
**Statistical approach to identify translationally-regulated mRNA molecules in LPS-activated moDCs**. The 54'675 probe sets present on the Affymetrix U133 Plus 2.0 GeneChip array were first filtered on flags (P in 50% 0 h or P in 50% 4 h or P in 50% 16 h) to obtain a preliminary list of 20'438 probe sets. A 2-way ANOVA analysis with repeats on time (false discovery rate= 0.05) was then performed on the 20'438 selected probe sets, to identify the 375 translationally-regulated mRNA molecules with statistically significant interaction (red circle in Venn diagram). An example of interaction (the two curves are parallel between 0 h and 4 h and are "interacting" between 4 h and 16 h post-LPS) between Total and Polysomal mRNA is indicated on the simplified graph on left, where the different time points of the two RNA groups are connected by their mean gene expression signals. The number of probe sets belonging to each parameter is indicated in brackets. See Additional file 4 for the complete list of translationally-regulated mRNA molecules. Groups defined within each parameter, Time: 0 h, 4 h and 16 h post-LPS; RNA type: Total mRNA and Polysomal mRNA.

The 54'675 probe sets present on the Affymetrix GeneChip array were first filtered on flags (P in 50% 0 h or P in 50% 4 h or P in 50% 16 h) to obtain a preliminary list of 20,438 probe sets. A 2-way ANOVA with repeats on time was performed on 20,438 selected probe sets (using a false discovery rate of 0.05). 375 probe sets (2%) had a statistically significant interaction (Figure 4), indicating that only a relatively small subset of mRNA molecules were translationally regulated in LPS-activated moDCs (complete list in Additional file 4). From this list of translationally regulated genes and using the Ingenuity Pathway analysis software (IPA version 6.3), we were able to identify several major "biological functions" controlled at the post-transcriptional level during the activation of moDCs (Table 1).

**Table 1:** Top "biological functions" of mRNA molecules affected by translation regulation in LPS-activated moDCs

**Category (nr. of molecules)**	**Biological Function**	***p*-value**	**Molecules (gene symbols)**
Protein Synthesis (26)	biosynthesis of proteins and translation regulation	1.73E-04	ADAMTS5, **CASP9**, EEF2, EIF2A, EIF4A2, **EIF4B**, NCK1, PLAU, PSMC2, RBM3, RPL3, RPL5, RPL7, RPL13, **RPL14**, RPL15, **RPL26**, RPL31, RPL37, RPL38, RPL9, RPS11, SERPINB1, SRGN, UBC, UBE2K
RNA Post-Transcriptional Modification (12)	modification of mRNA	8.67E-03	CDC5L, CPSF1, DBR1, EIF4A2, **EIF4B**, GRSF1, HNRNPR, CPSF1, GRSF1, MAPKAPK2, ZFP36, QARS
Amino Acid Metabolism (11)	catabolism of L-tryptophan and dephosphorylation of amino acids	8.57E-08	**CD80**, **IL6**, **INDO**, KMO, KYNU, DUSP2, DUSP3, PPM1A, PPM1F, PPP1CB, PTPN6
Cell Morphology (10)	transmembrane potential of mitochondria	1.26E-03	AP2A2, BNIP3, **CASP9**, CFLAR, **IL6**, MAPK9, MOAP1, NFE2L2, PTPN6, SRGN
Immune Cell Trafficking (8)	emigration of leukocytes	2.08E-03	CD44, CXCL3, CXCR4, CYTIP, GFI1, LDLR, PLAU
Cancer (7)	tumorigenesis of intestinal tissue	2.64E-03	APC, MLH1, IFI16, SMARCA2, HSPA1A, IFI16, IGFBP4
Nucleic Acid Metabolism (5)	metabolism of nucleic acid component	6.15E-04	ATIC, MTAP, **OAS1, OAS2**, REXO2
Cell-mediated Immune Response (4)	secretion of cytokine	3.60E-03	CADM1, LCP2, SRGN, GFI1
Antigen Presentation (2)	antigen peptide transporter	8.84E-03	**TAP1, TAP2**
Small molecule biochemistry (1)	activation by LPS	4.21E-02	**LY96 (MD2)**

The 375 probe sets with statistically significant interaction (Additional file 4) have been analysed with the Ingenuity Pathway software. The table displays the 10 most significant biological "categories" affected by translation regulation (out of 29 found), ranked by the number of molecules per category. The significance is expressed as *p*-value and is based on a Fisher's exact test, calculated by the software itself. The translationally regulated mRNA molecules validated by qPCR are indicated in bold (Table 2). RPL26 has been also validated at the protein level and is underlined (Figure 6).

The biological function with the most significative p-value for translational regulation was protein synthesis itself (26 molecules), including 3 translation factors among which was found eIF2α. Other identified pathways included "post-transcriptional modification" and "amino acid metabolism" comprising again some molecules involved in protein synthesis and amino acid modification (e.g. QARS, eIF4B, or INDO, DUSP2 and DUSP3).

As for molecules directly relevant to DC immune function, we identified genes involved in pathogen sensing (e.g.: OAS1, OAS2, LY96), antigen processing (e.g.: TAP1 and TAP2), immune regulation (e.g.: IL-6, INDO, CD80, SLP2) or leukocytes emigration (e.g. CXCL3 and CXCR4). Clearly this list indicates that a number of mRNAs expressed during DC maturation and important for their immuno-modulatory function are regulated at the translational level, such as Indoleamine 2,3-dioxygenase (IDO or INDO). IDO is a potent immuno-regulatory enzyme that degrades the essential amino acid tryptophan and results in a rise in uncharged tRNA, which activates the GCN2 kinase and downstream signaling such as the phosphorylation of eIF2α. Thus, IDO is likely to be preferentially translated in conditions of eIF2α phosphorylation and might therefore be regulated at the translational level.

A subset of transcripts from the original list of 375 translationally regulated genes was selected for real-time PCR validation (Table 2). The validation was performed on the total RNA and the polysome-bound RNA populations, for 14 different genes directly relevant to DC biology (see Additional file 5). The PCR results obtained on three independent experiments confirmed the microarray data for most of the selected genes, a fact further supported statistically by applying the Spearman correlation method (see Additional file 6). Although the level of translation regulation is relatively weak for some of these genes (e.g. TAP1, TAP2 or CASP9), several displayed a marked down-regulation of translation engagement while transcription remained unchanged (e.g. RPL 14 and RPL26 or MD2).

**Table 2:** Validation of the array data by real time PCR using total and polysome-bound RNA populations.

Gene Symbol	Affy probe set	RNA type	Fold change (p-value) Array		Fold change (+/- SD) qPCR	
			**4 h vs 0 h**	**16 h vs 4 h**	**4 h vs 0 h**	**16 h vs 4 h**

IL-6	205207_at	Poly	503,11 (0,040)	-7,3 (0,101)	3641,21 (11,92)	-58,26 (1,21)
		Tot	239,22 (0,010)	-7,3 (0,051)	2017,22 (19,13)	-12,95 (2,11)
RPL26	222229_x_at	Poly	1,41 (0,205)	-1,78 (0,044)	1,81 (0,32)	-1,06 (0,33)
		Tot	-1,36 (0,129)	-1,33 (0,321)	-3,12 (0,41)	-1,52 (0,34)
RPL14	213588_x_at	Poly	1,45 (0,008)	-1,47 (0,101)	0,31 (0,12)	-0,81 (0,22)
		Tot	-1,1 (0,191)	-1,17 (0,089)	-1,31 (0,32)	-1,29 (0,33)
RPS23	200926_at	Poly	1,73 (0,099)	-2,74 (0,100)	0,72 (0,13)	-2,52 (0,35)
		Tot	1,05 (0,011)	-1,72 (0,111)	0,32 (0,14)	-1,89 (0,07)
CD80	1554519_at	Poly	22,81 (0,133)	1,31 (0,002)	19,71 (0,72)	0,77 (0,22)
		Tot	33,65 (0,001)	1,43 (0,005)	22,71 (1,52)	0,41 (0,11)
OAS1	202869_at	Poly	11,56 (0,048)	1,36 (0,044)	7,26 (1,24)	1,74 (0,71)
		Tot	13,12 (0,026)	1,86 (0.003)	12,32 (0,32)	1,26 (0,11)
OAS2	204972_at	Poly	34,15 (0,043)	-1,16 (0,111)	7,51 (0.12)	1,48 (0,45)
		Tot	23,5 (0,001)	1,2 (0,011)	5,91 (0,11)	1,21 (0,22)
CASP9	203984_s_at	Poly	-3,94 (0,011)	2,92 (0,009)	-14,71 (1,3)	2,79 (0,81)
		Tot	-3,74 (0,005)	2,02 (0,001)	-11,7 (2,1)	3,09 (0,62)
HLA-F	221875_x_at	Poly	2,07 (0,021)	1,21 (0,008)	3,41 (0,42)	1,97 (0,32)
		Tot	1,92 (0,002)	1,97 (0,007)	2,31 (0,71)	2,25 (0,55)
INDO	210029_at	Poly	117,32 (0,022)	1,34 (0,001)	1176,21 (8,33)	2,01 (0,26)
		Tot	97,51 (0,011)	1,91 (0,008)	737,2 (3,55)	2,54 (0,27)
TAP1	202307_s_at	Poly	6,62 (0,340)	1,02 (0,009)	4,71 (1,1)	0,79 (0,20)
		Tot	8,38 (0,013)	1,31 (0,002)	6,2 (0,89)	1,55 (0,41)
TAP2	225973_at	Poly	3,19 (0,001)	1,56 (0,111)	3,01 (0,66)	1,62 (0,61)
		Tot	3,20 (0,013)	1,95 (0,505)	3,21 (0,52)	1,93 (1,10)
MD2	206584_at	Poly	-1,37 (0,008)	-3,43 (0,043)	-2,91 (0,77)	-3,30 (0,21)
		Tot	1,11 (0,008)	-2,31 (0,002)	1,51 (0,33)	-2,10 (0,41)
eIF4B	211938_at	Poly	-2,26 (0,001)	-1,65 (0,031)	-2,21 (0,23)	0,87 (0,04)
		Tot	-3,81 (0,006)	-1,66 (0,003)	-17,91 (2,21)	0,35 (0,02)

### Specific ribosomal proteins are translationally regulated in activated moDCs

**Figure 5:**
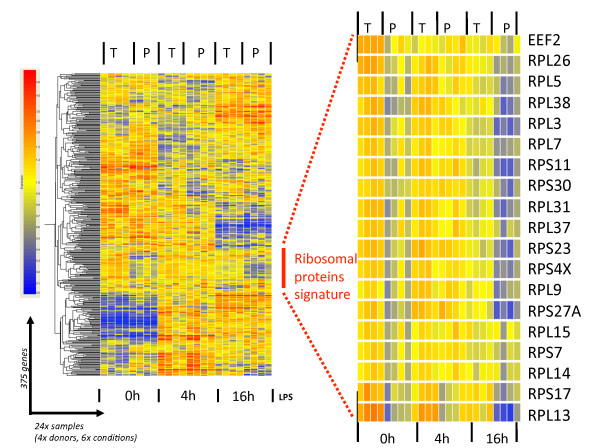
**Specific ribosomal protein mRNAs are translationally-regulated in LPS-activated moDCs**. The 375 mRNA molecules with statistically significant interaction (see Figure 4) were clustered on a heat-map using the software GeneSpring GX 7.3. The translationally regulated ribosomal protein mRNAs appear as a specific signature (left panel), which has been extracted and enlarged (right panel). See the text for more details.

To complement the Ingenuity pathway analysis and identify co-regulated mRNAs during DC- activation, the 375 translationally regulated transcripts were clustered on a heat-map using Genespring default setting. Once again, the most obvious cluster of translationally regulated mRNAs was a group of 11 different large ribosomal subunit proteins and translation factor genes, which are part of the protein synthesis pathway identified during the Ingenuity pathway analysis and validated by qPCR (Figure 5). The gene expression signals for this cluster containing, RPL3, RPL5, RPL7, RPL9, RPL13, RPL14, RPL15, RPL26, RPL31, RPL37 and RPL38 was considerably reduced in the polysomal-bound mRNAs compared to total mRNA signals at 0 h and 16 h post-LPS activation. Comparatively no significant difference among the two RNA types was detected after 4 h of LPS stimulation, suggesting that these RPL mRNAs were engaged in translation at this early time of DC activation. Thus, RPL transcripts seem to undergo "translational engagement" between 0 h and 4 h, followed by "translational disengagement" between 4 h and 16 h, thus matching the observed rate of protein synthesis in DCs. Transcription of ribosomal protein genes starts at a C-residue that is embedded in an oligopyrimidine tract of length 5-25 base pairs, also known as the 5'-TOP signal. The 5'-TOP signal is an essential *cis*-regulatory motif for the translational control of most ribosomal protein mRNAs expression [18,19]. A preponderance of evidence suggests that translation of mRNAs containing a 5'-TOP signal is strongly up-regulated in response to ribosome protein S6 phosphorylation triggered by mTORC1 stimulation and subsequent S6K1 activation. In addition to ribosomal proteins mRNAs, TOP mRNAs include also those coding for several translation elongation factors such as eEF2, indicating that most of the translationally down-regulated genes identified in DCs during their response to LPS contain a 5'-TOP signal.

In recent years, several reports have also suggested that translation of TOP mRNAs can be independent of S6 phosphorylation [20,21]. In activated DCs, S6 phosphorylation correlates well with the translational engagement of the ribosomal proteins after 4 h of LPS stimulation (Figure 5, 4h). However even if the intensity of S6 phosphorylation keeps increasing during the following 12 h of DC maturation, several of the large ribosomal protein mRNAs are nevertheless clearly disengaged from polysomes. Thus, although S6 phosphorylation is likely to favor translational engagement of 5'-TOP containing mRNAs, it does not counteract their increased release from the ribosomes.

**Figure 6:**
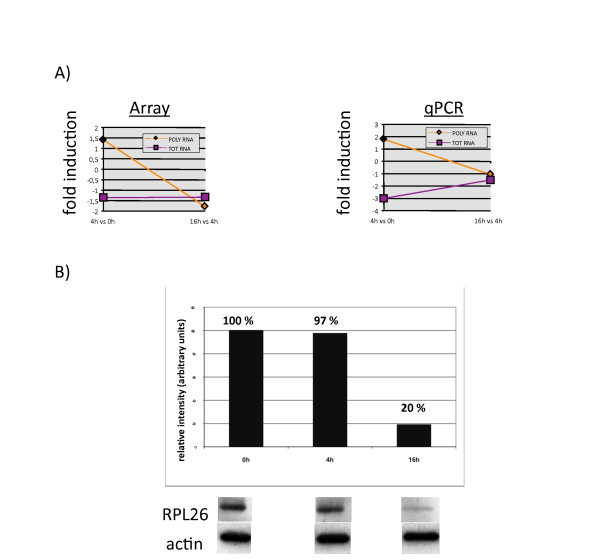
**Correlation between RPL26 mRNA translational disengagement and protein down-regulation in LPS-activated moDCs**. (A) Gene expression of the RPL26 Total and Polysomal mRNAs determined by microarrays analysis (left) and confirmed by qRT-PCR analysis (right), depicted as fold induction between 4 h and 0 h and 16 h and 4 h post-LPS. (B) Immunoblot to assay RPL26 protein expression at 0 h, 4 h and 16 h post-LPS. An actin immunoblot is shown for equal loading control. The relative protein expression (top) has been determined by quantifying the immublot signals with the software ImageQuant (Fuji) and is representative of a typical experiment (n = 3).

A role for ribosome proteins (RLP) outside of translation is emerging, however most of the current knowledge about these proteins links them with specific translation regulation activity including ER-binding for RLP7 and control of survival for RPL5, 11, 23 and 26 [22]. Indeed, these particular RLPs are involved in the murine double minute protein (MDM2)-mediated p53 pathway regulation [23]. Mdm2 is a E3-ubiquitin ligase known to regulate p53 levels directly by promoting its degradation and indirectly by targeting ribosomal protein L26, which normally binds the p53 mRNA and augments its translation. As the ribosomal protein L26 (RPL26) represents an important component of the auto-regulatory feedback loop that maintains low p53 activity in non-stressed cells [22], we further investigated the regulation of RPL26 at the mRNA and protein levels in activated DCs. Although RPL26 mRNA gene expression was stable upon-LPS stimulation, its polysomal-bound engagement was lost at 16 h, as identified by microarrays and confirmed by qPCR (Figure 6A). More importantly, RPL26 protein expression was down-regulated by 80% after 16 h of LPS- mediated activation, correlating with the down-regulation of polysomal-bound mRNAs (Figure 6B).

The translation regulation of ribosomal protein mRNAs is one of the features of LPS-activated human DCs. In the early phase of DC maturation, a specific increase in ribososomal proteins synthesis might impact positively on the global translation enhancement in response to TLR-4 engagement and subsequent PI3K activation, while a reduction at later time might favor a decrease in translation intensity. The reduction in RPL26 might also have a specific effect on the survival of activated DCs. Although many aspects of the non-translational role of ribosomal proteins remain to be investigated, it is noteworthy to underline that several ribosomal protein (RLP4, RLP22 and RLP35) and translation factors (eEF1d) mRNAs represent the most significant genes up-regulated in a statistical group comparison performed to identified genes differentially expressed in Systemic onset juvenile idiopathic arthritis (SoJIA) patients compared with healthy children [24]. SoJIA patients are also characterized by abundant production of interleukin (IL)-1, which is an important mediator of this disease and can be induced in DCs by LPS, thus creating an additional link between 5'-TOP containing mRNAs and inflammatory conditions. Our results on the characterization of RPL26 mRNA and its protein expression strongly support our conclusion that other RLP genes belong to a cluster being translationally regulated during DC maturation. Translation regulation therefore appears as a key function to control specific gene expression and more precise analysis combining traditional gene expression arrays and translation profiling will have to be carried-out to follow the immune response and host-pathogen interactions and single-out functionally important mRNAs regulated at the translation level.

## Methods

### Cell Culture

Fresh human leukapheresis products were obtained from the EFS (Marseille, France). Human peripheral blood mononuclear cells (PBMC) were isolated by Ficoll-PaqueTM PLUS (Amersham Biosciences, Uppsala, Sweden), washed four times with RPMI and CD14^+ ^cells were immunomagnetically purified with AutoMACS system following the protocol of the manufacturer (Miltenyi Biotech, Auburn, CA). Purified CD14^+ ^monocytes were analyzed using a FACSCalibur (Becton Dickinson, San Jose, CA), confirming the purity of CD14^+ ^cells to be 95%. To promote differentiation into iDC, the purified CD14^+ ^cells (0.5 × 10^6 ^cells/ml) were plated in 6-well plates (2 × 10^6 ^cells/well) and cultured in RPMI 1640 medium supplemented with 10% FCS, non essential amino acids, penicillin/streptomycin 100 ng/mL (>1000 U/ml), recombinant human GM-CSF and 20 ng/mL (>100 U/ml) IL-4 for 5 days (both from PeproTech, Rocky Hill, NJ). At days 2 and 4, half of the volume of the medium was replaced by fresh medium supplemented with GM-CSF and IL-4. For DC maturation, 100 ng/mL LPS (*Escherichia coli *type 026:B6; Sigma-Aldrich, St. Louis, MO) was added to the cells at day 5, for the indicated number of hours.

### Polysomal profiling by sucrose gradient fractionation

Polysome-bound mRNA molecules were enriched by sucrose gradient fractionation following the protocol originally developed by Garcia-Sanz and collaborators [25]. Briefly, 60 to 80 × 10^6 ^day 5 human moDCs were lysed in 1 ml of polysome buffer (10 mM Tris-HCl (pH 8), 140 mM NaCl, 1.5 mM MgCl2, 0.5% NP40, 0.1 mg/ml cycloheximide, and 500 units/mL RNasin (Promega, Madison, WI). After 10 min on ice, lysates were quickly centrifuged (10.000 × g for 10 sec at 4°C) and the supernatant was resuspended in a stabilizing solution (0.2 mg/ml cycloheximide, 0.7 mg/ml heparin, 1 mM phenylmethanesulfonyl fluoride). After a quick centrifugation (12.000 × g for 2 min at 4°C) to remove mitochondria and membrane debris, the resulting supernatant was layered on a 15% to 40% sucrose gradient. Gradients were then ultracentrifuged (35.000 × g for 2 h at 4°C, SW41 rotor) and after centrifugation 20 × 550 ml fractions were collected, starting from the top of the gradient. All the fractions were then digested with Proteinase K (200 mg/ml) in presence of 1%SDS and 10 mM EDTA. RNA was then extracted with Phenol/Chloroform/Isoamylalcohol (volume ratio 25:24:1) and precipitated with 2.5 Volumes of 100% Ethanol in presence of 0.8 M lithium chloride, necessary to get rid of heparin, a known inhibitor of RT activity [26]. After precipitation all the RNA were resuspended in 20 ml RNase free H_2_O. The correct fractionation of the polysomes was tested by detecting the different rRNA types on a 1% denaturing agarose gel. Total RNA was directly extracted out of moDCs without fractionation. Total and polysomal-bound RNAwere purified using the RNeasy miniprep kit (Qiagen, Chatsworth, CA). To exclude theamplification of genomic DNA, an on-column DNase digestion was performed using the RNase-Free DNase Set (Qiagen). The RNA Integrity Number (RIN-value) of all RNA types and timepoints was measured with the Agilent 2100 Bioanalyzer. RIN-values between 6.5 and 8.5 (mean RIN value = 7.6) were obtained, indicating that the RNA had sufficient integrity to be analyzed by microarrays.

### Affymetrix microarray hybridization and data mining

For each condition 100 ng of total or polysomal-bound RNA were employed to synthesize double-stranded cDNA using two successive reverse-transcription reactions according to standard Affymetrix protocols (GeneChip Two-Cycle Target Labelling, Affymetrix, Santa Clara, CA). Linear amplification with T7-RNA polymerase and biotin labelling were performed by in vitro transcription by standard Affymetrix procedures. The resulting biotin-labeled cRNA was fragmented and hybridized to the Affymetrix Human Genome U133 2.0 oligonucleotide 14,500-gene microarray chip for 16 h at 45°C. Following hybridization, the probe array was washed and stained on a fluidics station and immediately scanned on a Affymetrix GCS 3000 GeneArray Scanner. The data generated from the scan were then analyzed using the MicroArray Suite software (MAS 5.0, Affymetrix). The data derived from four independent experiments were normalized using the GC-RMA algorithm and bioinformatic analysis was performed using GeneSpring GX 7.3 (Agilent, Palo Alto, CA) and Statistics Analysis System (SAS v9.1.3). Probe selection was performed using 2-way ANOVAs accounting for repeated measures with a false discovery rate of 0.05. Hierarchical clustering was performed using the default clustering algorithm and setting in GX7.3.

### Quantitative real-time RT-PCR

Total RNA was extracted and purified using the RNeasy kit (Qiagen). To exclude the amplification of genomic DNA, an on-column DNase digestion was performed using the RNase-Free DNase Set (Qiagen). 1 μg of RNA was retro-transcribed using SuperScript II reverse transcriptase (Invitrogen) and random (pDN6) primers. First-strand cDNA templates were then used for PCR amplification of short (100 to 150 bp) exon fragments of the gene of interest using the appropriate primers (Additional file 4 shows the complete list of the 375 probe sets with statistically significant interaction). PCR was carried out using a Stratagene MX3000P *Real-Time *PCR System in complete SYBR Green PCR buffer (PE Biosystems, Warrington, UK) using 200 nM of each specific primer. A total of 20 μl of PCR mix was added to 5 μl of cDNA template, and the amplification was tracked via SYBR Green incorporation by using a Stratagene (La Jolla, CA) sequence detection system 4.01. Comparative real-time PCR was done in triplicate, including no-template controls. A dissociation curve was generated at the end of each PCR cycle to verify that a single product was amplified. Relative quantification of target cDNA was determined by calculating the difference in cross-threshold (*C*_t_) values after normalization to GAPDH signals, according to the Pfaffl method and the automated Excel-based program available (REST^©^). The sequences of all employed primers are available in Additional file 7.

### Immunodetection and antibodies

50 μg of TX-100 soluble material or 200.000 cells, lysed directly in Laemmli buffer, were loaded on 10% SDS-PAGE prior to immunoblotting and chemiluminescence detection (SuperSignal, Pierce, USA). SUnSET was performed as previously described [14] using A647-labelled mouse IgG2a anti-puromycin antibody (12D10). Antibody against RPL26 was from Abnova. For flow cytometry analysis, APC-conjugated anti-HLA-DR (L243 clone) and PE-conjugated anti-CD86 (clone IT2.2) were from BD PharMingen (San Jose, CA, USA). Anti- eIF2, phospho-eIF2 and -phospho-S6 were from Cell Signaling Technology (Beverly, MA, USA). For immunofluorescence analysis, MoDCs were let adhere on Alcyan blue coated-coverslips and surface stained with unlabelled antibody at 4°C for 30 min. After washes, coverslips were placed in warm medium for the indicated time and then fixed in 3% PFA for conventional staining with secondary antibodies.

### GEO Reviewers Link

Following link has been created to allow read-only access review of record GSE14000 and associated accessions, which are the Affymetrix gene expression data related to the submitted manuscript.

http://www.ncbi.nlm.nih.gov/geo/query/acc.cgi?token=jlalxuycqigwqnw&acc=GSE14000

## Competing interests

The authors declare that they have no competing interests.

## Authors' contributions

All authors read and approved the final manuscript. MC, GC, ES, AdG performed experiments. DB, GO, JB and DC contributed to bioinformatics and statistical analysis. MC, EG and PP designed experiments and wrote the manuscript.

## Supplementary Material

Additional file 1 Shows the validation of our microarray analysis in the human moDCs system. Click here for file

Additional file 2 **(A-D) shows a detailed description of the genes subsets related to the global alterations of total and polysomal-bound mRNA in LPS-activated moDCs described in Fig. **3. Click here for file

Additional file 3 **Shows a heatmap of the preliminary list of 20'438 probe sets filtered on flags described in Fig. **4. Click here for file

Additional file 4 **Shows the complete list of the 375 probe sets with statistically significant interaction and is related to Fig. **4. Click here for file

Additional file 5 **(A-E) shows the validation of the array data by real time qPCR using Total and Polysomal RNA populations, and is related to Table **2. Click here for file

Additional file 6 **(A-D) shows correlations between Array and PCR Data after Spearman correlations**. Click here for file

Additional file 7 **Shows the sequences of all employed primers for quantitative real-time PCR**. Click here for file
