# Supplementary material for: Ribosomal protein mRNAs are translationally-regulated during human dendritic cells activation by LPS
Source: Immunome Res. 2009 Nov 27;5:5. doi: 10.1186/1745-7580-5-5 (PMC2788525; doi:10.1186/1745-7580-5-5)
Supplement: Additional file 1 — Shows the validation of our microarray analysis in the human moDCs system. [file 1745-7580-5-5-S1.PDF]

Regulation of genes related to DC-maturation, determined by Affymetrix microarray analysis.

Affymetrix ID	Gene symbol	Description	moDCs + LPS		
			4h vs 0h	16h vs 4h	p-value
	co-stimulatory molecules				
1554519_at	CD80	CD80 antigen (CD28 antigen ligand 1, B7-1 antigen)	33,7	1,4	1,87E-07
1555689_at			39,4	1,3	2,24E-10
205685_at	CD86	CD86 antigen (CD28 antigen ligand 2, B7-2 antigen)	2,1	2,9	8,41E-08
205686_s_at			3,6	3,4	1,59E-10
210895_s_at			3,1	2,9	1,92E-07
	antigen presentation				
211799_x_at	HLA-C	major histocompatibility complex, class I, C	2,2	2,4	4,34E-04
221978_at	HLA-F	major histocompatibility complex, class I, F	2,8	-1,1	1,65E-06
210514_x_at	HLA-G	HLA-G histocompatibility antigen, class I, G	2,1	2,8	1,60E-03
211530_x_at			2,3	2,5	1,19E-02
205671_s_at	HLA-DOB	major histocompatibility complex, class II, DO beta	15,6	3,9	3,30E-08
	cytokines				
207160_at	IL12A	interleukin 12A (p35)	4,9	1,8	3,45E-09
207901_at	IL12B	interleukin 12B (p40)	177,2	-1,1	9,86E-09
208173_at	IFNB1	interferon, beta 1, fibroblast	107,9	-28,0	1,77E-09
