# Supplementary material for: Ribosomal protein mRNAs are translationally-regulated during human dendritic cells activation by LPS
Source: Immunome Res. 2009 Nov 27;5:5. doi: 10.1186/1745-7580-5-5 (PMC2788525; doi:10.1186/1745-7580-5-5)

A) Translationally regulated genes from 0h to 4h post-LPS (transcription UP)

<u>Translationally *up*-regulated:</u>

- immediate early response (IER3)
- bone marrow stromal cell antigen (BST2)
- stomatin (STOM)
- *ferritin heavy chain 1 (FTH1)*
- GTP-binding protein Ram (RAB27A)
- eukaryotic translation initiation factor 5 (EIF5)
- ubiquitin specific peptidase 25 (USP25)
- major histocompatibility complex, class I, F (HLA-F)
- ribosomal protein S27a (RPS27A)
- RNA binding motif, single stranded interacting protein 1 (RBMS1)
- HIV-1 rev binding protein 2 (HRB2)

<u>Translationally *down*-regulated:</u>

- enthoprotin (ENTH)
- cIAP1 = IAP (BIRC2)
- interferon-related developmental regulator 1 (IFRD1)
- major histocompatibility complex, class I, C (HLA-C)
- fragile X mental retardation (FMR1)
- proteasome subunit, α type1 (PSMA1)
- ubiquitin specific peptidase 15 (USP15)
- ADP-ribosylation factor 6 (ARF6)
- major histocompatibility complex, class II, DR beta 1 (HLA-DRB1)

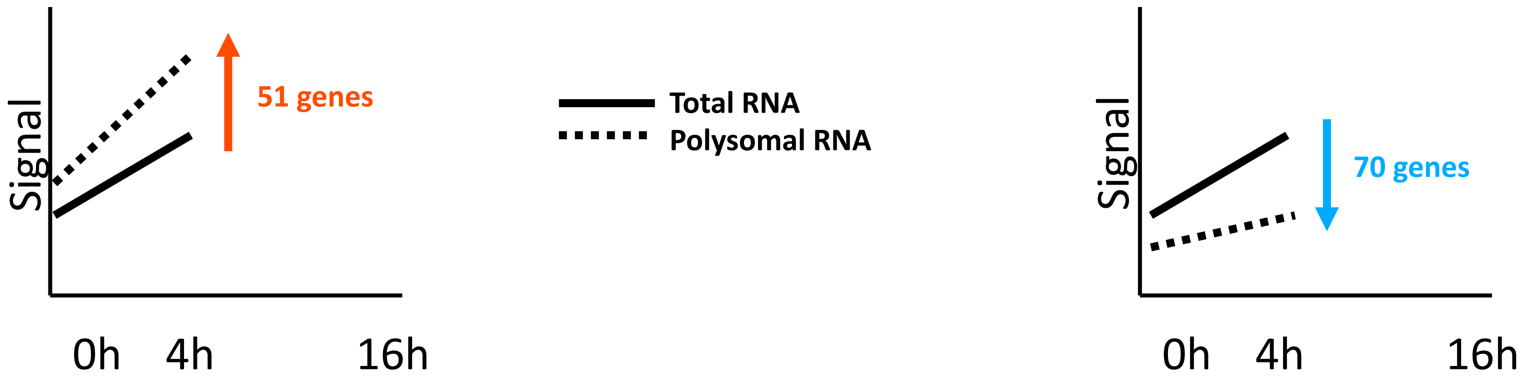

B) Translationally regulated genes from 0h to 4h post-LPS (transcription DOWN)

Translationally *up*-regulated:

translation regulation:

- MAP kinase interacting serine/threonine kinase 2 or MNK2 (MKNK2)
- protein-kinase, interferon-inducible double stranded RNA dependent inhibitor or P58 repressor (PRKRIR)
- ligatin (LGTN)
- glutaminyl-tRNA synthetase (QARS)
- phenylalanine-tRNA synthetase-like, beta subunit FARSLB)
- eukaryotic translation initiation factors: EIF4B**;** EIF2S3**;** EIF4A2**;** EIF3S7**;** EIF4EBP2

transcription regulation:

- BTB (POZ) domain containing 4
- SERTA domain containing 2
- general transcription factor IIIA
- death effector domain containing 2
- homeodomain interacting protein kinase 1
- vitamin D (1,25- dihydroxyvitamin D3) receptor
- ret finger protein
- nemo like kinase

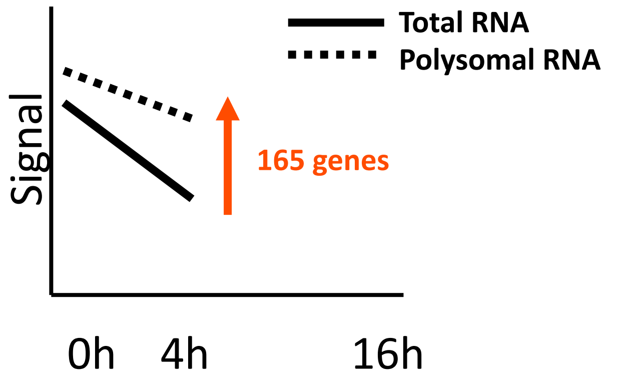

ubiquitination:

- ubiquitin specific peptidase 7 (herpes virus-associated) (USP7)
- ubiquitin protein ligase E3 component n-recognin 2, (UBR2)
-STIP1 homology and U-box containing protein 1 (STUB1)

cellular trafficking:

- RAB32, member RAS oncogene family (RAB32)
- RAB34, RAB34, member RAS oncogene family (RAB34)
- ADP-ribosylation factor-like 7 (ARL7)
- golgi autoantigen, golgin subfamily a, 3 (GOLGA3)
- transmembrane emp24 protein transport domain containing 8 (TMED8)
- transmembrane emp24 protein transport domain containing 1 (TMED1)
- importin 7 or Ran-binding protein 7 (IPO7)

autophagy:

- regulator of G-protein signalling 19 (RGS19)

cytoskeleton organization:

- adducin1 or ADDA (ADD1)

signalling:

- caspase recruitment domain family, member 4 (CARD4)
- inositol 1,3,4-triphosphate 5/6 kinase, (ITPK1)
- vitamin D receptor (VDR)

immune response:

- immunoglobulin (CD79A) binding protein 1 (IGBP1)
- SOCS or cytokine inducible SH2-containing protein (CISH)

B) Translationally regulated genes from 0h to 4h post-LPS (transcription DOWN)

Translationally *down*-regulated:

immune response:
- interleukin 1 receptor, type I (ILR1)
- chemokine (C-C motif) receptor 1 (CCR1)
- C-type lectin domain family 4, member A (CLEC4A)

signalling:
- sorting nexin 8 (SNX8) ???
- stress-associated endoplasmic reticulum protein 1 (SERP1)
- mitogen-activated protein kinase 1 (MAPK1)
- Phosphoinositide-3-kinase, class 2, alpha polypeptide (PIK3C2A)

viral response:
- barrier to autointegration factor 1 (BANF1)

ubiquitination:
- ubiquitin specific peptidase 50 (USP50)

apoptosis:
- cytokine induced apoptosis inhibitor 1 (CIAPIN1)
- catenin (cadherin-associated protein), alpha-like 1 (CTNNAL)

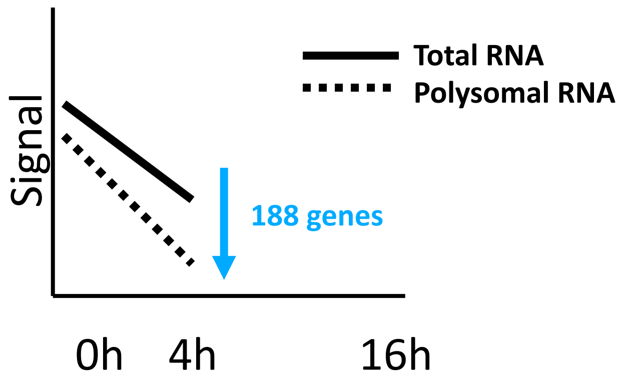

translation regulation:
- JTV1 gene (JTV1)
- mitochondrial ribosomal protein L1, L9, L15, L16, L18, L19, L22, L36 (MRPL-X)
- eukaryotic translation initiation factors: EIF2B1, EIF3S9, EIF2B4

transcription regulation:
- SERTA domain containing 2 (SERTAD2)
- zinc finger and BTB domain containing 1 (ZBTB1)
- Kruppel-like factor 13 (KLF13)
- PHD finger protein 3 (PHF3)
- CCR4-NOT transcription complex, subunit 8 (CNOT8)
- prolactin regulatory element binding (PREB)
- zinc finger protein 45 (ZNF45)
- SWI/SNF related, matrix associated, actin dependent regulator of chromatin, subfamily a, member 4 (SMARCA4)
- protein inhibitor of activated STAT, 1 (PIAS1)
- activity-dependent neuroprotector (ADNP)
- zinc finger, HIT type 3 (ZNHIT3)
-transcription factor CP2 (TFCP2)

cellular trafficking:
- chromatin modifying protein 2B (CHMP2B)
- peroxisomal biogenesis factor 7 (PEX7)
- RAN binding protein 6 (RANBP6)
- Chloride channel 3 (CLCN3)
- ralA binding protein 1 (RALBP1)
- ADP-ribosylation factor guanine nucleotide exchange factor 2 (brefeldin A-inhibited) (ARFGEF2)
- RAB guanine nucleotide exchange factor (GEF) 1 (RABGEF1)

C) Translationally regulated genes from 4h to 16h post-LPS (transcription UP)

Translationally *up*-regulated:

transcription regulation: 3 genes (5%)

immune response:

antigen presentation:

Translationally *down*-regulated:

immune response: 15 genes (10%)
- interleukin 10 receptor, beta (IL10RB)
- interferon gamma receptor 1 (IFNGR1) (?)
- interferon gamma receptor 2 (IFNGR2)
- protectin – present in exosomes (CD59)
- CD164 antigen, sialomucin (CD164)
- interferon induced transmembrane protein 2 (IFITM2)
- interferon induced transmembrane protein 3 (IFITM3)
- bone marrow stromal cell antigen 2
- colony stimulating factor 2 receptor, alpha, low-affinity (granulocyte-macrophage) (CSF2RA)
- DKFZP564J0863 protein (DKFZP564J0863)
- antigen presentation:

major histocompatibility complex, class I, B, C, E (?), F,

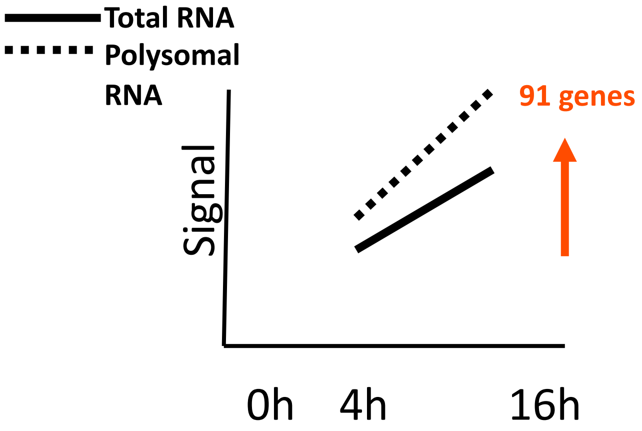

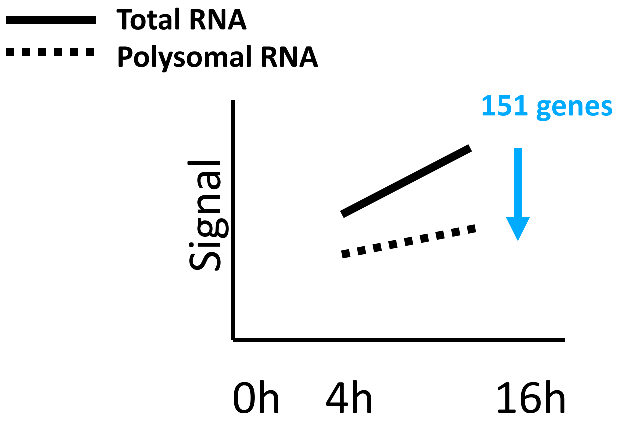

D) Translationally regulated genes from 4h to 16h post-LPS (transcription DOWN)

<u>Translationally *up*-regulated:</u>

<u>transcription regulation: 3 genes (5%)</u>

- retinoic acid induced 17 (RAI17)
- mastermind-like 2 (MAML2)
- general transcription factor IIA, 2, (GTF2A2)

<u>other biological process: <3 genes (51%)</u>

<u>unknown function: 24 genes (44%)</u>

<u>translation regulation: NO GENES</u>

<u>Translationally *down*-regulated:</u>

<u>translation regulation: 53 genes (42%):</u>

- eukaryotic translation initiation factors:
EIF3S3, EIF3S8
-eukaryotic translation elongation factors:
EEF1G (3x probesets), EEF2K,
- ribosomal proteins small subunit (21 proteins):
RPS3, RPS3A, RPS4X, RPS5, RPS6, RPS7, RPS8, RPS10, RPS11, RPS12, RPS14, RPS15A, RPS16, RPS18, RPS19, RPS21, RPS23, RPS24, RPS25, RPS27A, RPS30
- ribosomal proteins large subunit (26 proteins):
RPL3, RPL4, RPL5, RPL6, RPL7, RPL8, RPL9, RPL13, RPL13A, RPL19, RPL22, RPL23, RPL23A, RPL27A, RPL29, RPL30, RPL31, RPL32, RPL34, RPL35A, RPL36A, RPL38, RPLP2, RPLP0, RPL22L1
- other proteins:
cysteinyl-tRNA synthetase (CARS)
glycyl-tRNA synthetase (GARS)

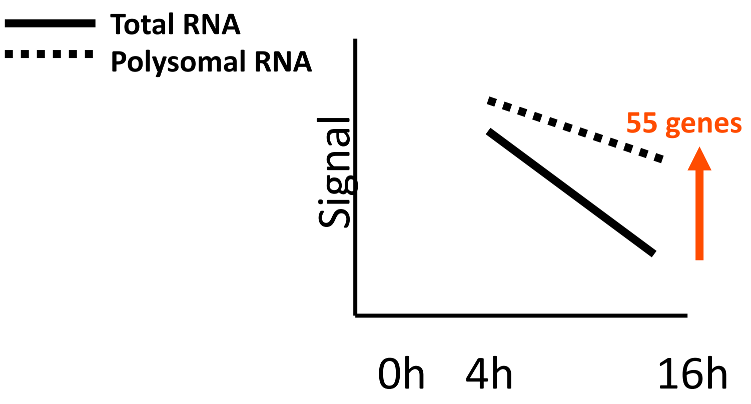

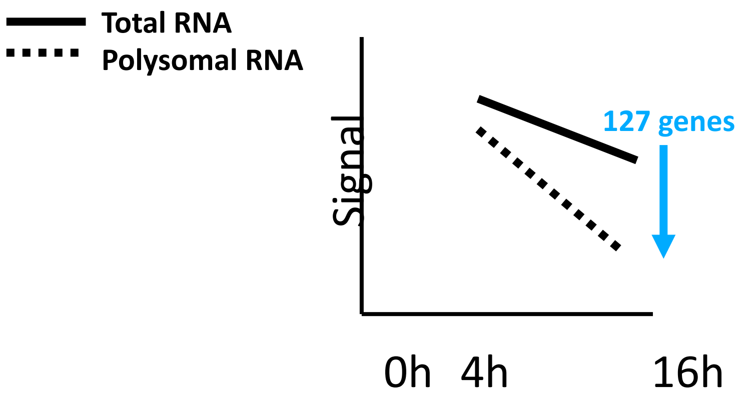

Supplement: Additional file 2 — (A-D) shows a detailed description of the genes subsets related to the global alterations of total and polysomal-bound mRNA in LPS-activated moDCs described in Fig. 3. [file 1745-7580-5-5-S2.PDF]
