# Supplementary figures and images for: Ribosomal protein mRNAs are translationally-regulated during human dendritic cells activation by LPS
Source: Immunome Res. 2009 Nov 27;5:5. doi: 10.1186/1745-7580-5-5 (PMC2788525; doi:10.1186/1745-7580-5-5)

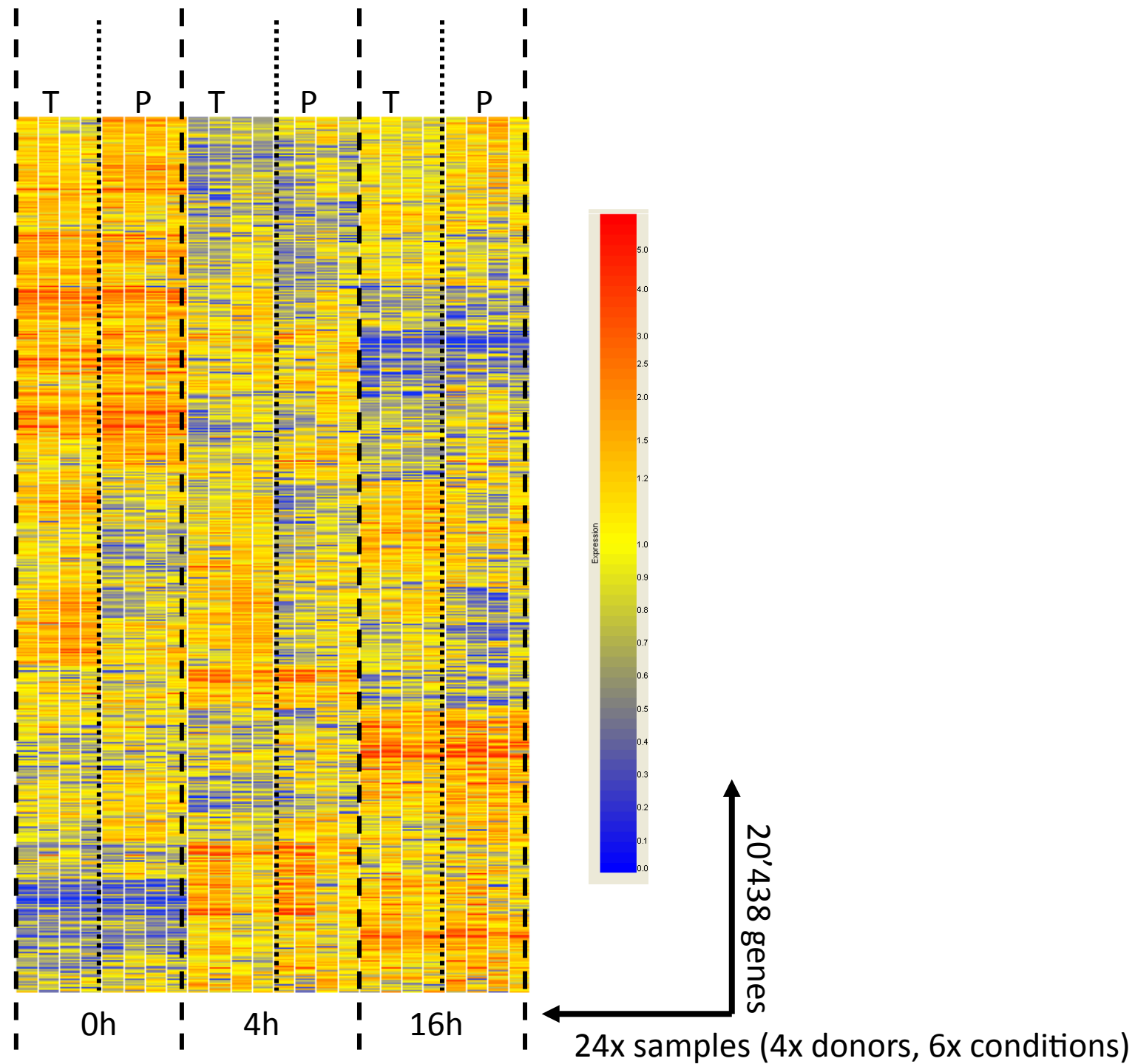

Supplement: Additional file 3 — Shows a heatmap of the preliminary list of 20'438 probe sets filtered on flags described in Fig. 4. [file 1745-7580-5-5-S3.PDF]
