# Supplementary material for: Ribosomal protein mRNAs are translationally-regulated during human dendritic cells activation by LPS
Source: Immunome Res. 2009 Nov 27;5:5. doi: 10.1186/1745-7580-5-5 (PMC2788525; doi:10.1186/1745-7580-5-5)
Supplement: Additional file 4 — Shows the complete list of the 375 probe sets with statistically significant interaction and is related to Fig. 4. [file 1745-7580-5-5-S4.PDF]

Table S2: 375 probe sets with statistically significant interaction

condition ()*	0h tot		0h poly		4h tot		4h poly		16h tot		16h poly																					
timepoint ()*S	0h		0h		4h		4h		16h		16h																					
Systematic	Normalized	Raw	Normalized	Raw	Normalized	Raw	Normalized	Raw	Normalized	Raw	Normalized	Raw	Common	Synonyms	Genbank	Gene Symb	Map	Chromosom	EC	Description	Product	Phenotype	Function	DBid	GO biologic	GO cellular	GO molecul	RefSeq	UniGene	PubMedID	Type	User Notes
65588_at	1.315415	33.775	0.95081	24.025	0.670032	17.075	1.0720171	27.15	1.245518	31.875	0.782252	22.075			AA827892		20q12	20		CDNA clone IMAGE:3880075, partial cds									Hs.400876			
64438_at	0.985203	89.75	0.996111	91.4	0.76535	69.8	0.53486395	48.625	1.138968	99.925	1.023568	90.20001	FLJ22222		W19668		17q25.3	17		hypothetical protein FLJ22222									Hs.436237			
46142_at	1.373382	29.7	1.098764	23.375	1.080123	23.1	1.1150539	23.5	0.578321	12.575	0.866672	18.075	FLJ12681		AI003763		16p13.3	16		hypothetical protein FLJ12681									Hs.58362			
38149_at	0.693486	648.2	0.856542	825.05	1.098863	1030.425	0.992936	948.1	1.258637	1192.55	1.024778	1026.575	ARHGAP25		D29642		2p13.3	2		KIAA0053 gene product				GI:40789060					Hs.1528			
33132_at	1.723687	224.15	1.920598	256.725	0.632327	109.925	0.9990268	141.9	0.466333	61.225	0.279099	43.85	CPSF160; P/cl.18; HSU3		U37012	CPSF1	8q24.23	8		cleavage and polyadenylation specific factor 1, 160kDa	cleavage and polyadenylation specifi			GeneID:298	GO:6379(m	GO:5847(m	GO:3723(R	NM_01329	Hs.493202	15489334;	protein-coding	
32069_at	0.451494	1677.95	0.798871	2857.875	0.811903	3035.725	1.334007	4780.6	1.05915	3944.075	1.431495	5195.075	N4BP1		AB014515		16q12.1	16		Nedd4 binding protein 1	KIAA0615 protein			GI:40788302		nucleus			Hs.323712			
244663_at	1.344127	22.725	2.564886	43.975	1.26571	20.6	0.6478717	11.8	0.58602	10.025	0.489243	8.95			AI675336			0		Transcribed sequences									Hs.156256			
244520_at	0.97548	37.05	1.203391	44.4	1.08023	41.925	1.1783208	46.675	0.661664	23.675	1.290303	50.3			AW206728			0		Transcribed sequences									Hs.129094			
243444_at	1.079438	92.775	0.527012	51.2	1.195163	106	0.75234723	72.35	0.911156	79.02499	0.921113	84.475			AI221894			0		qq99q08.x1 Soares_NFL_T_GBC_S1 Homo sapiens cDNA clone IMAGE:1843358 3' similar to contains Alu repetitive element;, mRNA sequence.												
241418_at	0.471069	26.325	0.62911	34.3	2.351828	123.825	1.7070578	90.575	1.172342	61.5	0.887245	47.675			AI819386			0		MRNA; cDNA DKFZp686B14224 (from clone DKFZp686B14224)									Hs.531333			
239879_at	1.988253	65.025	1.638796	54.625	0.149494	4.325	0.89509076	26.6	1.126575	36	1.158478	34.575	LOC284998		N63566		2q12.2	2		CDNA FLJ39302 fis, clone OCBBF2012990									Hs.410180			
239753_at	1.341264	25.85	0.864154	16.05	0.733219	14.975	1.1291218	20.95	1.132433	22.2	0.792427	15.5			BE560888			0		601346327F1 NIH_MGC_8 Homo sapiens cDNA clone IMAGE:3679471 5', mRNA sequence.												
239346_at	0.755837	76.975	1.320239	136.9	0.740999	75.85001	0.9123516	92.425	1.70032	172.975	1.713836	177.05			AI097560			0		Transcribed sequence with weak similarity to protein ref:NP_060265.1 (H.sapiens) hypothetical protein FLJ20378 [Homo sapiens]									Hs.530977			
239225_at	1.425281	97.9	1.52288	100.4	0.699892	56.175	0.93576795	64.675	0.319954	22.6	1.014195	79.025			AI161380			0		Transcribed sequence with weak similarity to protein sp:P39188 (H.sapiens) ALU1_HUMAN Alu subfamily J sequence contamination warning entry									Hs.525603			
239161_at	1.070958	45.45	0.973389	42.6	2.053683	90	1.3606158	59.975	0.507338	22.05	0.915939	39.65	FDX1		BE302085		11q22	11		ferredoxin 1									Hs.744			
238953_at	1.748242	26.075	1.644468	20.5	1.904588	24.975	0.22683215	3	0.386277	5.875	0.594614	9.35			AA993833			0		CDNA clone IMAGE:5206119, partial cds									Hs.318639			
238509_at	0.464008	23.775	0.425754	19.925	1.966218	84.625	0.5251932	25.475	1.94383	82.925	1.212515	53.2	CUL1		AI628926		7q36.1	7		cullin 1									Hs.348153			
237400_at	0.755251	17.05	0.323463	7.425	1.712404	38.525	1.4811014	33.375	0.604788	15.625	1.16527	28.675	ATP5S		AI308101		14q22.1	14		ATP synthase, H+ transporting, mitochondrial F0 complex, subunit s (factor B)									Hs.440456			
236772_s_at	1.350771	47.05	1.737698	60.075	0.971924	32.6	0.90206766	29.475	0.69831	24.925	1.128856	39.15			AA554330			0		Transcribed sequences									Hs.146125			
236656_s_at	0.649007	9.7	0.281563	5.375	3.020515	36.95	1.6006215	20.35	1.502273	18.675	0.166619	2.05			AW014647			0		Full length insert cDNA YI37C01									Hs.432924			
235837_at	1.043506	67.82501	0.779927	51.825	0.892503	57.175	1.1620828	75.6	1.004215	65.55	1.125655	74.55			AA813998			0		Transcribed sequence with moderate similarity to protein ref:NP_054848.1 (H.sapiens) PRO0478 protein [Homo sapiens]									Hs.47232			
235773_at	1.582603	69.125	1.193226	53.25	0.72414	31.25	0.9088095	39.525	0.704512	31.575	1.206124	53.85	LOC284307		AW029293		19q13.43	19		hypothetical protein LOC284307									Hs.454830			
235751_s_at	1.285813	42.475	0.52184	18.95	0.697255	23.05	0.76663476	27.35	2.571286	105.95	1.335382	66.875	LOC284013		AA977975		17p13.3	17		secretory protein LOC284013									Hs.122561			
235320_at	1.510971	27.85	1.716107	33.9	0.794505	17.1	0.61704737	12.525	0.644582	12.175	0.927212	17.225	ARL6		AL138043		3q12.1	3		DKFZp761M1815_r1 761 (synonym: hamy2) Homo sapiens cDNA clone DKFZp761M1815 5', mRNA sequence.												
235146_at	0.173049	53.975	0.332256	97.8	0.865744	228.5	1.1791185	317.75	4.974675	1362.425	5.176966	1467.375	KIAA1145		N51717		12q22	12		KIAA1145 protein									Hs.173392			
235141_at	0.529335	71.89999	0.45007	63.95	0.763102	98.7	0.93665445	140.85	3.122662	427.2	4.894507	687.35	MRVLDC2		AW009562		5q13.1	5		hypothetical protein FLJ30532									Hs.390601			
235061_at	0.150477	128.225	0.098335	85.5	1.475427	1272.25	1.0128495	892	2.082816	1754.225	0.785161	708.825	DKFZp761G058		AV706522		4q22.1	4		AV706522 ADB Homo sapiens cDNA clone ADBDAF07 5', mRNA sequence.												
234931_at	1.314641	54.5	0.730995	30.2	1.142255	47.15	0.8383141	36.175	0.944034	38.825	1.152357	47.95	AYP1p1		Z48511			0		H.sapiens XG mRNA (clone PEP11).												
234512_x_at	1.709618	2592.175	0.926269	1411.025	1.138585	1736.225	1.5421082	2347.05	0.777532	1153.625	0.507348	780.275	RPL7A		AL136226		9q34	9		dJ486D24.1 (Ribosomal protein L7A pseudogene) match: proteins: Sw:P17076 Sw:P29453; Human DNA sequence from clone RP3-486D24 on chromosome 6 Contains a ribosomal protein L7A (RPL7A) pseudogene, a CpG island, ESTs, GSSs and STSs, complete sequence.												
234295_at	0.614083	133.45	1.514944	315.45	0.480579	100.275	0.874159	183.6	0.933	197.725	1.371813	293.375	DBR1		AK000116	DBR1	3q22.3	3		debranching enzyme homolog 1 (S. cerevisiae)	debranching enzyme homolog 1			GeneID:511	GO:375(RN	GO:5634(n	GO:8419(R	NM_01621	Hs.477700	17081983;	protein-coding	
233632_s_at	0.237889	88.6	0.355386	129.3	1.356696	543.625	1.6917347	672.15	1.494395	553.05	1.115845	432.025	SEP1; FLJ41903; DKFZp		AL117516	XRN1	3q23	3		5'-3' exoribonuclease 1	5'-3' exoribonuclease 1 isoform a			GeneID:544	GO:7049(c	GO:5737(c	GO:3677(D	NM_01900	Hs.435103	17081983;	protein-coding	
233200_at	1.127869	20.2	0.694115	12.125	1.257232	22.875	1.3129369	23.675	0.73491	13.575	1.272601	22.75			AF161364			0		HSPC101 mRNA, partial cds	HSPC101			GI:6841142					Hs.259683			
233013_x_at	2.622875	442.925	0.959302	156.6	0.977733	162	0.64422333	105.975	1.956813	319.625	0.782684	135.625	LOC220906		AU146717		10p12.1	10		Transcribed sequence with moderate similarity to protein sp:P39193 (H.sapiens) ALU6_HUMAN Alu subfamily SP sequence contamination warning entry									Hs.372013			
232422_at	1.193952	78.47501	0.640409	42.35	0.937349	63.325	0.60067266	40.25	1.222023	81.07501	1.262663	88.875	LOC87769		BC001077		13q32.3	13		hypothetical protein BC004360	LOC87769 protein			LocusID:87769; GI:33875960					Hs.187191			
232087_at	0.701811	49.95	1.090708	78.05	0.938033	66.275	1.0887228	78.675	1.140513	81.3	1.171664	88.85001	LOC256643		AW628045		xp22.13	23		hypothetical protein LOC256643									Hs.28896			
231959_at	0.980699	57.525	1.172001	66.225	0.363545	24.625	0.08749151	5.35	1.582191	89.02499	0.820546	61.45001	C14orf46		AK023979		14q24.2	14		chromosome 14 open reading frame 46									Hs.351381			
231920_s_at	0.821113	104.575	0.606643	85.55	1.202927	157.325	1.4879481	229.575	1.037319	129.125	0.539017	79.9	CSNK1G1		AK025179		15q22.1-q2	15		casein kinase 1, gamma 1									Hs.405789			
231212_x_at	1.199007	59.8	0.804112	40.25	1.183583	59.55	1.2127709	60.25	0.750978	37.475	1.075314	55.525			AA161476			0		zq49q09.s1 Stratagene hNT neuron (#937233) Homo sapiens cDNA clone IMAGE:633088 3' similar to contains Alu repetitive element;contains element MER28 repetitive element ;, mRNA sequence.												
230934_at	2.071994	111.8	0.98424	52.75	1.340599	69.625	0.9469956	49.35	0.49638	28.85	0.924656	50.525			BF508609			0		RAB3 GTPase-ACTIVATING PROTEIN									Hs.306327			
230657_at	1.048739	134.275	0.905597	120.175	1.021413	129.55	0.75614595	96.5	1.15289	155.05	1.527021	197.525	CLOCK		AI423466		4q12	4		clock homolog (mouse)									Hs.436975			
230550_at	1.91112	1333.5	2.432918	1596.125	1.299529	805.375	0.88145125	534.825	0.029033	24.75	0.023935	18.725	MS4A6A		AA045175		11q12.1	11		membrane-spanning 4-domains, subfamily A, member 6A									Hs.371612			
230422_at	0.812829	2712.625	0.814051	2714.475	1.521129	5158.425	1.3027722	4317.725	0.97364	3763.225	0.495929	2072.975	FPRL2		AW026543		19q13.3-q1	19		formyl peptide receptor-like 2									Hs.445466			
230376_at	3.564265	161.2	1.489418	69.575	1.408538	65.35	0.6429696	31	0.676854	31.85	0.472272	22.675			AI339915			0		Transcribed sequences									Hs.44324			
229764_at	1.211902	274.05	1.253544	284.05	2.513061	490.625	1.6875595	381.675	0.605134	93.875	0.382283	69.525	FLJ41238		AW629527		3q28	3		FLJ41238 protein									Hs.338851			
229625_at	0.103306	49.175	0.069055	35.2	5.94295	3239.925	7.594252	4199.775	1.473322	873.575	1.377978	1070.45	GBP5		BG545653		1p22.2	1		guanylate binding protein 5									Hs.237809			
229602_at	2.564584	43.35	2.553528	42.95	1.465377	25.025	0.57238674	11.125	0.267906	5.15	0.782827	12.575			H97567			0		Transcribed sequences									Hs.129777			
229390_at	0.210486	1272.55	0.185268	1030.55	1.222232	6279.475	1.5362024	7642.125	1.146251	5855.275	1.308856	6717.175			AV734646			0		chromosome 6 open reading frame 187									Hs.381220			
228852_at	0.753117	61.775	0.919734	72.575	1.268069	100.775	0.91546255	72.225	1.508365	119.9	1.22703	98.625	ENSA		AV726322		1q21.3	1		endosulfine alpha									Hs.511916			
228826_at	1.160806	67.125	0.719373	43.2	1.467727	84.45001	1.1187774	65.05	0.688793	40.875	0.755477	44.85			AK000271			0		Clone IMAGE:5755907, mRNA									Hs.180804			
228707_at	0.626167	46.55	0.880464	65.8	0.750039	54.35	0.76579756	58.1	1.520228	113.425	2.550232	201.875	CLDN23		BF110053		8p23.1	8		claudin 23									Hs.183617			
228700_at	1.332964	152.85	1.466659	171.525	1.045905	120.8	1.1279138	129.9	0.890609	99.05	0.510622	58.05	MGC39350		AA063608		Xp11.4	23		ze87e08.s1 Soares_fetal_heart_NbHH19W Homo sapiens cDNA clone IMAGE:365990 3' similar to contains Alu repetitive element;, mRNA sequence.												
228584_at	0.907498	26.3	0.853956	23.875	1.157557	29.675	0.53242546	14.725	0.96114	24.3	1.040431	27.3	SGCB		AI089025		4q12	4		sarcoglycan, beta (43kDa dystrophin-associated glycoprotein)									Hs.438953			
228562_at	0.752972	95.95	0.911764	114.475	0.984983	137.95	1.0829474	145.5	2.045941	269.7	1.850906	262.575	ZBTB10		N29918		8q13-q21.1	8		zinc finger and BTB domain containing 10									Hs.436991			
228439_at	0.277865	31.825	0.170985	21.6	1.214943	146.075	1.1590089	135.1	1.063355	119.775	1.236799	143.625	MGC20410		AW083820		11q13.1	11		hypothetical protein BC012330									Hs.446698			
228301_x_at	2.616125	632	0.910822	223.225	1.365358	338.1	0.72116333	190.825	0.844506	217.475	0.557827	144.6	NDUFB10		AA854479		16p13.3	16		ai75a10.s1 Soares_parathyroid_tumor_NbHPA Homo sapiens cDNA clone IMAGE:1402266 3' similar to SW:NIDM_BOVIN Q02373 NADH-UBIQUINONE OXIDOREDUCTASE PDSW SUBUNIT ;, mRNA sequence.												
228258_at	3.476829	113.2	2.576706	86.75	0.899577	32.15	0.30068412	12.275	0.330758	15.95	0.513831	18.475	FLJ00332		AW008519		11q13.1	11		ribosomal protein S6 kinase, 70kDa, polypeptide 2									Hs.32156			
228167_at	0.679194	139	1.321958	272.775	1.098492	226.025	1.6463871	345.825	0.399899	94.2	0.613022	143.575	KLHL6		AW574798		3q27.3	3		kelch-like 6 (Drosophila)									Hs.439354			
228024_at	0.987653	145.975	1.528222	234.4	1.022358	150.475	1.0206145	160.1	0.570994	84.45	1.05632	171.575	FLJ32642		AW028100		8p22	8		hepatocellular carcinoma related protein 1									Hs.309697			
227872_at	1.344016	81.95001	1.006241	60.575	0.368465	24.1	0.72815406	48.6	1.055818	64.025	1.147736	69.97501	RPC155		AA527570		10q22-q23	10		polymerase (RNA) III (DNA directed) (155kD)									Hs.436896			
227840_at	0.729561	109.275	1.463098	222.2	0.979998	143.85	1.0936534	171.05	1.072584	159.85	1.300436	201.05	LOC130355		AA738440		2q14.2	2		Hypothetical protein LOC130355, mRNA (cDNA clone IMAGE:5189916), partial cds									Hs.99488			
227728_at	3.176872	335.55	2.732365	295.025	0.729985	78.875	0.81302416	102.675	0.726384	78.425	1.14645	127.375	PPM1A		AA886888		14q23.1	14		protein phosphatase 1A (formerly 2C), magnesium-dependent, alpha isoform									Hs.130036			
227682_at	0.535834	181.55	0.838405	267	1.698983	567.95	1.6640115	550.275	1.015835	317.475	0.962318	311.425			BE645154			0		Transcribed sequence with weak similarity to protein pir:A45973 (H.sapiens) A45973 trichohyalin - human									Hs.99216			
227680_at	0.393314	69.325	0.981759	172.375	0.573769	100.375	1.2329837	219.375	1.479221	273.8	2.397358	435.125	FLJ20403		AI057121		1p22.2	1		hypothetical protein FLJ20403									Hs.306221			
227609_at	0.051413	421.65	0.07367	593.1751	0.91791	7149.85	1.1297114	8686.45	1.180631	9006.075	1.261935	9668.351	EPSTI1		AA633203		13q13.3	13		epithelial stromal interaction 1 (breast)									Hs.343800			
227559_at	2.269781	154.25	1.863929	126.975	0.99872	65.55	0.73508275	48.8	0.628574	41.75	0.872831	57.475			AL521129			0		Transcribed sequence with strong similarity to protein pir:S55507 (H.sapiens) S55507 protein disulfide-isomerase									Hs.406328			
227539_at	0.664962	248.525	0.865323	314.25	1.994977	748.975	1.5630857	586.175	0.90354	337.025	0.862545	343.9	GNA13		AW298099		17q24.3	17		guanine nucleotide binding protein (G protein), alpha 13									Hs.9691			
227347_x_at	0.018867	3.05	0.031731	4.9	3.922479	683.575	1.2741846	227.275	1.52974	232.725	0.457991	97.85001	HES4		NM_02117	HES4	1p36.33	1		hairy and enhancer of split 4 (Drosophila)	hairy and enhancer of split 4			GeneID:578	GO:30154(	GO:5634(n	GO:3677(D	NM_02117	Hs.154029	16710414;	protein-coding	
227344_at	1.015788	421.225	0.854959	353.175	0.748334	318.3	0.7419058	320.9	1.324877	553.8	1.495227	637.6	ZNFN1A1		AI439886		7p13-p11.1	7		zinc finger protein, subfamily 1A, 1 (Ikaros)									Hs.435949			
227203_at	1.650567	549	1.578967	525.125	0.873742	285.175	0.91876745	298.075	0.709348	230.175	1.306182	443.35	FBXO13		AA115629		5q21.3	5		F-box only protein 13									Hs.179112			
227185_at	1.928082	156.575	0.821215	63.775	1.321913	102.9	0.929581	71.72501	1.050363	82.77499	0.898842	73.45			BE378494			0		Similar to RIKEN cDNA B930041F14 gene (LOC388769), mRNA									Hs.6872			
227178_at	0.975858	223.3	1.052445	237.975	0.784204	180.425	0.66843855	158.725	1.239549	283.125	0.949372	218.35	CUGBP2		AI652861		10p13	10		CUG triplet repeat, RNA binding protein 2									Hs.211610			
227141_at	1.625147	648.375	0.834375	333.775	0.842541	350.125	0.6008366	244.35	1.480952	613.15	1.334206	565.35	LOC127253		AW205739		1p31.1	1		UI-H-BI1-afv-f-03-0-UI.s1 NCI_CGAP_Sub3 Homo sapiens cDNA clone IMAGE:2723093 3', mRNA sequence.												
227138_at	1.963448	106.125	1.291513	71.125	1.190298	64.05	0.9362694	50.775	0.34566	18.6	0.564934	31.675	LOC253263		AA025858		3p22.3	3		MRNA; cDNA DKFZp586B1024 (from clone DKFZp586B1024)									Hs.483205			
226994_at	0.776108	632.225	1.432148	1177.725	0.728362	595.925	1.0730867	872.925	0.952246	771.875	2.128852	1870.225	DNAJA2		AW057513		16q11.1-q1	16		DnaJ (Hsp40) homolog, subfamily A, member 2									Hs.368078			
226767_s_at	0.997267	73.97501	1.324544	98.625	0.558811	47.375	0.5288742	39.225	1.489166	109.225	0.676933	54.775	DKFZP566J2046		AI341467		16p13.3	16		hypothetical protein DKFZp566J2046									Hs.322482			
226762_at	1.058942	140.2	0.976335	132.575	1.465635	205.025	1.1706493	156.325	0.64768	86.45	0.635101	86.675	PURB		AV709094		7p13	7		CDNA FLJ33107 fis, clone TRACH2000959									Hs.349150			
226633_at	0.330684	1580.675	0.537824	2569	0.844709	4058.675	1.079338	5185.275	1.467964	6993.475	1.617459	7800.2	RAB8B		AI807023		15q22.1	15		RAB8B, member RAS oncogene family									Hs.365655			
226630_at	0.749546	50.175	1.444008	98.14999	0.444734	28.925	0.4086161	27.3	1.567383	102.925	1.78712	122.475	C14orf106		BF062175		14q21.3	14		chromosome 14 open reading frame 106									Hs.437941			
226603_at	0.109061	487.275	0.172415	765.575	1.153921	5428.775	1.5334921	6930.325	1.122696	4940.925	1.271744	5590.725	FLJ39885		BE966604		7q21.3	7		601660289R1 NIH_MGC_71 Homo sapiens cDNA clone IMAGE:3905950 3', mRNA sequence.												
226087_at	1.488302	479.55	0.629421	203.4	1.087038	357.825	0.62450373	208.1	1.342876	435.7	0.993027	326.325	LZIC		BG180887		1p36.22	1		leucine zipper and CTNNBIP1 domain containing									Hs.327252			
225973_at	0.256301	205.475	0.417351	330.275	0.820388	737.975	1.331272	1131.775	1.555857	1327.075	2.082272	1723.525	TAP2		AA573502		6p21.3	6		transporter 2, ATP-binding cassette, sub-family B (MDR/TAP)									Hs.502			
225785_at	1.353246	862.175	1.053222	720.3	1.274159	802.925	0.8990098	631.55	0.718524	465.4	0.713094	468.625			BG112359			0		CDNA clone MGC:24463 IMAGE:4082362, complete cds									Hs.530633			
225634_at	0.950655	434.5	1.160763	527.575	1.986644	978.35	1.8342594	864.275	0.179571	84.925	0.129746	65.77499	ZC3HAV1		BG533558		7q34	7		zinc finger CCCH type, antiviral 1									Hs.35254			
225559_at	0.996974	133.85	1.173337	159.275	1.090393	147.475	0.9417417	126.4	0.936566	125.2	1.26076	170.025	FLJ33839		AK023163	C3orf19	3p25.1	3		chromosome 3 open reading frame 19	hypothetical protein LOC51244			GeneID:51244; HGNC:28033; HPRD:HPRD_1282(				NM_01647	Hs.517820	16189514;	protein-coding	
225507_at	0.998447	182.4	1.243478	243.725	0.707481	128.7	0.5544694	105.75	1.081482	195.225	1.157886	215.3	C6orf111		BF591408		6q16.3	6		chromosome 6 open reading frame 111									Hs.414993			
225477_s_at	1.173408	115.775	1.900426	177.125	0.553229	55.35	0.9955289	94.55	0.948702	87.55	0.962298	89.95	NR2C2		AL138444		3p25	3		mitochondrial ribosomal protein S25									Hs.458338			
225386_s_at	0.723814	414.275	1.233455	697.1	2.356545	1517.325	2.9635937	1903.175	0.347903	202.925	0.448934	261.15	LOC92906		AI559701		2p22.3	2		hypothetical protein BC008217									Hs.404336			
225146_at	1.31907	77.8	0.926774	53.3	0.899158	52.85	0.6646651	40.3	1.18802	69.35	1.653657	99.77501	C9orf25		AW005237		9p13.2	9		chromosome 9 open reading frame 25									Hs.7961			
224981_at	1.172289	641.275	0.229048	121.2	1.955428	1034	1.1235873	623.975	1.605431	842	0.567056	311.525	LOC124446		AL520900		16p12.1	16		Prostate derived STE20-like kinase PSK (PSK), mRNA									Hs.291623			
224931_at	1.367747	122.3	1.24208	109.325	0.729993	67.475	0.8926895	80.375	0.353766	33.1	0.951258	84.9	FLJ20473; SLC41A1-L2		AK021925	SLC41A3	3q21.2	3		solute carrier family 41, member 3	solute carrier family 41, member 3 is			GeneID:549	GO:6812(cation transpo		GO:8324(c	NM_00100	Hs.573007	15302935;	protein-coding	
224905_at	1.208497	528.175	1.442429	637.75	1.011569	442.175	0.98809206	433.3	0.438974	192.75	0.698549	308.825	WDR26		AA482548		1q42.12-q4	1		zt34b03.s1 Soares ovary tumor NbHOT Homo sapiens cDNA clone IMAGE:724205 3', mRNA sequence.												
224826_at	0.974524	178.25	1.337571	241.675	0.686419	125.4	0.5930478	107.1	1.052458	205.625	1.579696	299.15	KIAA1434; FLJ11085; N		AK001947		20p12.3	20		hypothetical protein KIAA1434	hypothetical protein LOC56261			RP5-1022P	GO:6071(glycerol metal		GO:8889(gl	NM_01959	Hs.636359	14702039;	protein-coding	
224807_at	0.239557	103.3	0.170741	77.2	0.98336	460.675	1.1010145	565.625	2.080337	912.5	2.063992	934.125	FLJ22411; FLJ90346; K		AB040966	GRAMD1A	19q13.11	19		GRAM domain containing 1A	GRAM domain containing 1A			GeneID:57655; HGNC:29305; HPRD:HPRD_1115				NM_02089	Hs.515351	16303743;	protein-coding	
224763_at	2.884287	66.95	1.735017	40.525	1.188591	28.375	0.8136842	18.975	0.490484	12	0.768295	19.1			BF724210			0		MRNA; cDNA DKFZp434J0428 (from clone DKFZp434J0428)									Hs.506072			
224714_at	1.297895	449.6	1.863174	646.65	0.408779	142.225	0.6905629	240.375	0.884476	309.725	1.291571	466.675	MKI67IP		AL542544		2q14.3	2		MKI67 (FHA domain) interacting nucleolar phosphoprotein									Hs.367842			
224444_s_at	1.161037	107.575	0.404421	40.375	1.100191	101.875	0.60053796	56.95001	1.459182	129.15	1.153867	101.95	FLJ27347; FLJ27348; N		BC005997	C1orf97	1q32.3	1		chromosome 1 open reading frame 97	hypothetical protein LOC84791			GeneID:84791; HGNC:28227; HPRD:HPRD_1750(				NM_03270	Hs.523932	16710414;	protein-coding	
224356_x_at	1.38942	3559.825	1.478838	3776.35	1.644019	4081.7	1.0514251	2709.2	0.049032	201.2	0.037771	141.35	CDA01; MS4A6; 4SPAN		AF237908	MS4A6A	11q12.1	11		membrane-spanning 4-domains, subfamily A, member 6A	membrane-spanning 4-domains, subf			GeneID:642	GO:7165(si	GO:16021(i	GO:4872(re	NM_02234	Hs.523702	15489334;	protein-coding	
224187_x_at	1.389565	6827.7	1.214546	6008.5	0.858144	4225.05	1.0418125	5248.15	0.693287	3501.275	0.559965	3051.725	HSPA8		AF217511		11q24.1	11		heat shock 70kDa protein 8	uncharacterized bone marrow protei			GI:7688965					Hs.180414			
223746_at	0.684164	11.45	0.995472	11.725	0.473835	7.75	1.7179109	20	1.415228	16.45	0.534362	6.925	KRS2; MST1; YSK3; DKF		BC005231	STK4	20q11.2-q1	20		serine/threonine kinase 4	serine/threonine kinase 4			GeneID:678	GO:6915(a	GO:16021(i	GO:5524(A	NM_00628	Hs.472838	17192257;	protein-coding	
223703_at	2.296169	277.4	1.055189	127.875	1.811761	221.175	0.9861426	120	0.519995	63.475	0.532357	66.35	CDA017		AF267860	C10orf11	10q22.2-q2	10		chromosome 10 open reading frame 11	chromosome 10 open reading frame			GeneID:83938; HGNC:23405; HPRD:I			GO:5515(p	NM_03202	Hs.118161	16964243	protein-coding	
223526_at	1.2784	265.8	1.095776	230.775	0.90086	187.35	0.8057575	169.3	0.837216	174.25	1.122711	232.4	XTP13; HsT3108; PNAS		AF277189	C18orf21	18q12.2	18		chromosome 18 open reading frame 21	chromosome 18 open reading frame			GeneID:83608; HGNC:28802; HPRD:HPRD_1268(				NM_03144	Hs.37883	16964243;	protein-coding	
223491_at	0.775164	319.1	1.237237	517.425	0.795302	327.375	0.81025666	342.9	2.375543	989.8001	2.384908	1016.175	HSPC042; MGC57611		BC001228	COMMD2	3q25.1	3		COMM domain containing 2	COMM domain containing 2			GeneID:51122; HGNC:24993; HPRD:HPRD_1308				NM_01609	Hs.591315	15799966;	protein-coding	
223434_at	0.181389	167.65	0.30988	276.825	1.241123	1104.35	1.6882077	1520.9	1.576686	1505.825	1.365303	1260.025	FLJ10961; DKFZp686E(		AL136680	GBP3	1p22.2	1		guanylate binding protein 3	guanylate binding protein 3			GeneID:263	GO:6955(immune respo		GO:5525(G	NM_01828	Hs.656774	16710414;	protein-coding	
223402_at	3.95607	483.775	1.136808	144.325	2.06446	270.25	0.5697069	82.25	0.270657	43.625	0.264381	39.075	VHZ; MOSP; LDP-3; DUS		BC001140	DUSP23	1q23.2	1		dual specificity phosphatase 23	dual specificity phosphatase 23			GeneID:549	GO:6470(p	GO:5634(n	GO:16787(	NM_01782	Hs.425801	16710414;	protein-coding	
223344_s_at	0.558471	164.7	0.607246	205.375	2.790103	821.375	1.3582668	508.175	1.091971	262.375	0.73347	201.825	CFFM4; MS4A8; 4SPAN2		AB026043	MS4A7	11q12	11		membrane-spanning 4-domains, subfamily A, member 7	membrane-spanning 4-domains, subf			GeneID:584	GO:7165(si	GO:16021(i	GO:4872(re	NM_02120	Hs.530735	16303743;	protein-coding	
223220_s_at	0.11379	190.9	0.228683	380.975	0.936948	1587.7	1.3911282	2320.825	1.200162	2002.125	1.30588	2245.6	BAL; BAL1; FLJ26637; I		AF307338	PARP9	3q13-q21	3		poly (ADP-ribose) polymerase family, member 9	poly (ADP-ribose) polymerase family			GeneID:836	GO:16477(	GO:5634(n	GO:3950(N	NM_03145	Hs.518200	16809771;	protein-coding	
223039_at	1.207086	1197.5	0.692003	782.0501	0.96562	945.2001	0.8251789	900.125	1.488446	1513.75	0.994762	1109.225	MGC1842		BC004144		22q11.2	22		Homo sapiens hypothetical protein MGC1842, mRNA (cDNA clone IMAGE:2819509), with apparent retained intron.												
223023_at	2.228025	167.725	0.970124	73.35	0.959855	71.22501	0.79631364	59.725	2.127984	158.675	0.922526	74.85	GS15; BET1L1; HSPC19		BC000688	BET1L	11p15.5	11		blocked early in transport 1 homolog (S. cerevisiae)-like	blocked early in transport 1 homoloc			GeneID:512	GO:15031(	GO:16021(integral to m		NM_01652	Hs.414418	15489334;	protein-coding	
223015_at	1.205741	1019.2	0.987696	844.575	0.84911	719.375	1.0601553	896.65	1.052808	897.3	0.980656	828.325	CDA02; EIF-2A; MST08		AF212241	EIF2A	3q25.1	3		eukaryotic translation initiation factor 2A, 65kDa	eukaryotic translation initiation facto			GeneID:839	GO:6417(r	GO:5737(c	GO:43022(	NM_03202	Hs.655782	16928686;	protein-coding	
222977_at	1.126313	395.2	0.646868	231.175	1.413144	493	0.99128586	361.8	0.99639	349.925	0.833669	298.225	SURF4		AL518882		9q34.2	9		surfeit 4									Hs.284296			
222872_x_at	0.208265	842.6	0.246148	992.125	1.655614	7002.6	1.810682	7441	1.118656	4533.425	0.838323	3417.925	FLJ22833		AU157541		2q32.3	2		hypothetical protein FLJ22833									Hs.118183			
222845_x_at	1.119636	4912.825	1.011821	4423.15	1.046879	4593.95	1.1451175	5030.725	0.797657	3468.825	1.026432	4499.75	S1R; ZPRO; CGI-119		AF161526	TMBIM4	12q14.1-q1	12		transmembrane BAX inhibitor motif containing 4	transmembrane BAX inhibitor motif c			GeneID:51643; HGNC:2		GO:16021(i	GO:5515(p	NM_01605	Hs.505934	17353931;	protein-coding	
222788_s_at	1.013238	77.45001	0.790485	66.55	1.200924	88.7	0.64841604	56.5	1.027819	76.5	1.111229	89.82501	FLJ11220		BE888593		1p13.1	1		hypothetical protein FLJ11220									Hs.425559			
222435_s_at	1.319216	295.4	0.514245	118.425	1.543611	319.925	0.78431195	166.85	1.376079	285.1	0.79283	165.6	Ubc6p; CGI-76; NCUBE1		AF161502	UBE2J1	6q15	6	6.3.2.19	ubiquitin-conjugating enzyme E2, J1 (UBC6 homolog, yeast)	ubiquitin-conjugating enzyme E2, J1			GeneID:514	GO:6464(p	GO:5783(e	GO:16874(	NM_01602	Hs.163776	16720581;	protein-coding	
222410_s_at	0.471359	1593.525	0.838002	2771.25	0.827286	2760.875	1.240907	4089.325	1.005572	3252.15	1.339538	4378.925	TFAF2; MGC3157; MSTP		AF121856	SNX6	14q13.2	14		sorting nexin 6	sorting nexin 6 isoform a			GeneID:585	GO:7154(c	GO:5737(c	GO:5478(in	NM_02124	Hs.583855	17148574;	protein-coding	
222403_at	1.616136	318.5	1.407378	285.3	1.25917	253.6	1.0414124	219.05	0.466186	91.60001	0.497748	100.025	MTCH2		AV756532		11p11.2	11		mitochondrial carrier homolog 2 (C. elegans)									Hs.279609			
222229_x_at	1.727256	1738.175	0.757484	769.1	1.268085	1283.7	1.0690846	1084.2	0.95244	955.125	0.601033	604.85	RPL26		AL121871		17p13	17		Human DNA sequence from clone RP13-258O15 on chromosome X, complete sequence.												

222129_at	2,237932	289,675	2,42296	312,5	0,636332	90,55	0,5183566	73,8	0,850615	108,35	1,175023	152,9	C2orf17	AK026155		2q36.1	2		chromosome 2 open reading frame 17							Hs.22412	
222122_s_at	0,858732	256,15	1,303473	394,05	0,954285	283,05	1,4072313	412,025	0,7408	219,9	0,766429	230,125	THOC2	BG403671		xq25-q26.3	23		THO complex 2							Hs.81376	
222010 at	1.229402	254.4	0.933069	191.175	1.231968	255.45	1.053686	215.125	0.690869	142.25	0.83364	172	ACAT2	BF224073		6q25.3-q2	6		acetyl-Coenzyme A acetyltransferase 2 (acetoacetyl Coenzyme A thiolase)							Hs.278544	
221891 x at	1.191833	6448.1	1.074756	5862.05	0.884856	4863.425	1.1201382	6190.6	0.739249	4072.975	0.689736	3865.75	HSPA8	AA704004		11q24.1	11		heat shock 70kDa protein 8							Hs.180414	
221879_at	1,965307	233,175	2,252155	270,825	0,977587	104,8	1,1178079	123,3	0,236722	26,925	0,126937	15,925	MGC4809	AA886335		15q22.31	15		serologically defined breast cancer antigen NY-BR-20							Hs.528715	
221875_x_at	0,464397	1841,6	0,544911	2250,625	0,893652	3591,7	1,1292707	4758,45	1,760055	6923,425	1,369728	5578,8	HLA-F	AW514210		6p21.3	6		major histocompatibility complex, class I, F							Hs.411958	
221806_s_at	0,892824	113,625	0,907761	118,25	1,061941	135,125	0,6247902	84,95	1,12639	142,15	0,863125	118,625	FLJ10707	BF590997		3p25.3	3		hypothetical protein FLJ10707							Hs.405917	
221770 at	1.021083	159.575	1.712004	273.975	0.548066	89.45	0.5862623	98.975	1.224211	196.175	1.016466	159.825	RPE	BE964473		2q32-q33.	2		601658180R1 NIH_MGC_68 Homo sapiens cDNA clone IMAGE:3876354 3', mRNA sequence.								
221571 at	1.343869	302.575	1.12342	249.85	1.057118	234.95	1.0120205	225.275	0.199718	49.5	0.248717	58.45	TRAF3	AI721219		14q32.33	14		as68b11.x1 Barstead colon HPLRB7 Homo sapiens cDNA clone IMAGE:2333853 3', mRNA sequence.								
221524_s_at	1,711403	932,3001	0,789071	478,45	0,863402	506,1	0,44545713	290,725	1,566886	906,05	0,544999	331,55	RAGD; bA11D8.2.1; DK	AF272036	RRAGD	6q15-q16	6		Ras-related GTP binding D	Ras-related GTP binding D	GeneID:585	cell growth	GO:5737(c	GO:5525(G	NM_02124	Hs.485938	15489334; protein-coding
221517_s_at	0,398957	98,65	1,194361	269,15	0,713643	165,325	1,138467	266,95	1,190027	273,1	1,479316	353,55	MED17; CRSP77; DRIP8	AF105421	CRSP6	11q14	11		cofactor required for Sp1 transcriptional activation, subunit	cofactor required for Sp enhances vi	GeneID:944	GO:30521(	GO:119(me	GO:16455(	NM_00426	Hs.444931	16109376; protein-coding
221475_s_at	1,182985	12469,6	0,724681	7912,275	1,044297	11054,18	1,0963489	11715,18	0,936043	9906,7	0,817524	8836,675	EC45; RPL10; RPLY10;	NM_00294	RPL15	3p24.2	3		ribosomal protein L15	ribosomal protein L15	GeneID:613	GO:6412(tr	GO:5622(in	GO:3723(R	NM_00294	Hs.381219	16608517; protein-coding
221210 s at	1.357732	381.1	2.219148	623.975	0.904147	241.65	1.1225094	296.5	0.373196	106.425	0.642292	181.45	NPL1; c112; C1orf13;	NM 03076	NPL	1q25	1		N-acetylneuraminate pyruvate lyase (dihydrodipicolinate syn	N-acetylneuraminate pyruvate lyase	GeneID:808	GO:5975(carbohydrate		GO:8747(N	NM 03076	Hs.496969	16710414; protein-coding
220789 s at	0.726164	107.625	1.161689	178.225	0.681213	100.65	0.9864708	147.65	1.52801	232.775	1.31399	217.25	CPR2; FASTKD4; KIAA0	NM 00474	TBRG4	7p14-p13	7		transforming growth factor beta regulator 4	cell cycle progression 2 protein isofo	GeneID:923	GO:6915(a	GO:5739(m	GO:5524(A	NM 00474	Hs.231411	16964243; protein-coding
220445_s_at	0,12985	2,225	0,119998	1,975	1,795684	33,625	0,47110945	15,525	11,10009	186,475	5,372806	107,5	TRAG3; MGC149851; M	NM_00490	CSAG2	Xq28	23		CSAG family, member 2	CSAG family, member 2	GeneID:959	GO:42493(response to drug)			NM_00490	Hs.522810	16888034; protein-coding
220439_at	1,18108	22,375	1,02398	20,2	0,97508	19,1	0,519613	10,75	0,570691	13,075	0,964641	21,05	RIN3	NM_024892		14q32.13	14										
220330_s_at	0,415426	1383,65	0,707225	2310,075	0,908987	3025,975	1,240361	4128,4	1,921902	6090,625	1,868938	5961,325	HACS1; NASH1	NM_02213	SAMSN1	21q11	21		SAM domain, SH3 domain and nuclear localization signals 1	SAM domain, SH3 domain and nuclea	GeneID:64092; HGNC:10528; HPRD:I			GO:1784(p	NM_02213	Hs.570423	15489334; protein-coding
220058 at	1.015063	24.425	1.86239	41.325	0.412619	10.025	0.12742536	2.925	1.402706	30.925	1.059789	25.6	MGC3048	NM 02405	C17orf39	17p11.2	17		chromosome 17 open reading frame 39	hypothetical protein LOC79018	GeneID:79018; HGNC:28453; HPRD:HPRD_1268				NM 02405	Hs.187422	15489334; protein-coding
219935 at	1.304331	20.55	0.680298	17.4	1.182217	13.85	0.8107969	13.725	0.146227	2.225	0.924873	13.8	ADMP-2; ADAMTS11; FL	NM 00703	ADAMTS5	21q21.3	21		ADAM metallopeptidase with thrombospondin type 1 motif,	ADAM metallopeptidase with thromb	GeneID:110	GO:6508(p	GO:5578(p	GO:5178(in	NM 00703	Hs.58324	17265493; protein-coding
219457_s_at	1,891591	167,625	1,363227	119,2	1,031029	91,2	0,955746	83,9	0,070929	6,975	0,161924	17,325	FLJ11700; FLJ22439;	NM_02483	RIN3	14q32.12	14		Ras and Rab interactor 3	Ras and Rab interactor 3	GeneID:798	GO:6897(e	GO:5575(c	GO:5096(G	NM_02483	Hs.326822	15489334; protein-coding
219279_at	0,762084	557,325	1,223118	886,625	0,493601	340,075	0,6209652	439,125	1,405775	987,575	1,523419	1068,45	DOCK10	NM_017718		2q36.3	2										
219215_s_at	2,917384	214,525	0,99525	71,025	1,35186	98,125	0,8823949	62,95001	0,833902	59,225	0,696251	54,825	AEZ; ZIP4; FLJ20327; M	NM_01776	SLC39A4	8q24.3	8		solute carrier family 39 (zinc transporter), member 4	solute carri Acrodermatitis enteropa	GeneID:556	GO:6811(io	GO:16021(i	GO:46873(	NM_01776	Hs.521934	17202136; protein-coding
219157 at	1.468033	583.9999	1.554271	626.05	0.980532	402.675	0.69605947	312.525	0.670854	270.2	0.575657	246.85	MAV; MAYVEN; ABP-KEL	NM 00724	KLHL2	4q21.2	4		kelch-like 2, Mayven (Drosophila)	kelch-like 2, Mayven	GeneID:112	GO:6886(in	GO:15629(	GO:3779(a	NM 00724	Hs.388668	16189514; protein-coding
219138 at	1.095099	110.15	1.005221	128.825	0.667539	71.225	1.6077635	190.575	0.74758	76.5	1.554984	222.2	L14; RL14; hRL14; CTG-	BC000606	RPL14	3p22-p21.	3		ribosomal protein L14	ribosomal protein L14	GeneID:904	GO:6412(tr	GO:5842(c	GO:3723(R	NM 00103	Hs.446522	17081983; protein-coding
218976_at	3,025915	67,425	5,360732	119,025	0,804907	24,525	0,91488844	30,725	0,597846	12,8	0,450029	10,175	JDP1; RP11-57G10.2	NM_02180	DNAJC12	10q22.1	10		DnaJ (Hsp40) homolog, subfamily C, member 12	J domain containing protein 1 isoform	RP11-57G1	GO:6457(protein folding		GO:31072(	NM_02180	Hs.260720	16391838; protein-coding
218819_at	1,085216	445,9	0,944273	388,775	0,977796	413,45	0,67261285	285,425	1,122804	461,975	1,194394	521,4	HDB; INT6; DBI-1; DDX2	NM_01214	INTS6	13q14.12-	13		integrator complex subunit 6	integrator c Nonsmall cell lung cance	RP11-24B1	GO:16180(	GO:32039(	GO:5524(A	NM_01214	Hs.439440	16271964; protein-coding
218800_at	0,871622	37,95	0,533761	24,35	1,187931	53,775	1,2613299	55,075	1,056795	47,025	1,207931	53,8	SRD5A2L	NM_024592		4q12	4		steroid 5 alpha-reductase 2-like	steroid 5 alpha-reductase 2-like	GeneID:796	superoxide	GO:16021(i	GO:3865(3	NM_02459	Hs.590906	14702039; protein-coding
218740 s at	1.146082	319.625	1.382234	385.15	1.168093	316.7	1.6007332	448.8	0.640744	173.625	0.560092	158.2	C53; IC53; HSF-27; MST	NM 02519	CDK5RAP3	17q21.32	17		CDK5 regulatory subunit associated protein 3; synonyms: C	CDK5 regulatory subunit associated	GeneID:802	GO:7420(b	GO:8372(c	GO:42808(	NM 02519	Hs.20157	15790566; protein-coding
218716 x at	1.212057	168.25	2.623114	360.775	1.014584	141.15	1.0506546	152.275	0.825713	112.5	0.953039	130.875	CGI-02	NM 01212	MTO1	6q13	6		mitochondrial translation optimization 1 homolog (S. cerevis	mitochondrial translation optimizatio	GeneID:258	GO:6118(el	GO:5739(m	GO:50660(	NM 01212	Hs.347614	15901843; protein-coding
218714_at	1,202306	145	0,661668	79,175	1,030654	123,8	0,94285804	115,425	1,377742	164,25	1,043191	123,75	MGC3121; DKFZp781A1	NM_02403	PRR14	16p11.2	16		proline rich 14	proline rich 14	GeneID:78994; HGNC:28458; HPRD:HPRD_1454				NM_02403	Hs.293629	16189514; protein-coding
218712_at	0,970254	30	2,32742	70,95	0,604409	19,075	0,6537551	20,65	1,13225	33,675	1,544411	47,375	FLJ20508	NM_01785	C1orf109	1p34.3	1		chromosome 1 open reading frame 109	hypothetical protein LOC54955	GeneID:54955; HGNC:26039; HPRD:HPRD_0791				NM_01785	Hs.272673	15489334; protein-coding
218611_at	0,22267	645,35	0,424769	1221,6	0,750814	2239,4	1,3713624	3996,8	1,196197	3463,425	1,629935	4750,9	SBBI48; MGC102760	NM_01654	IER5	1q25.3	1		immediate early response 5	immediate early response 5	GeneID:51278; HGNC:5393; HPRD:HPRD_06210;				NM_01654	Hs.15725	16710414; protein-coding
218549 s at	1.208496	509.2	1.163888	499.6	0.657898	321	0.52116966	260.475	0.878637	373.4	1.19052	518.225	CGI-90; FLJ20665	NM 01603	FAM82B	8q21.3	8		family with sequence similarity 82, member B	hypothetical protein LOC51115	GeneID:51115; HGNC:24285; HPRD:I			GO:5488(bi	NM 01603	Hs.145386	15489334; protein-coding
218467_at	1,136269	2579,55	0,922397	2094,325	0,764691	1736,8	0,862799	1965,4	1,219654	2787,55	1,181542	2737,675	PAC2; HCCA3; CLAST3;	NM_02023	TNFSF5IP1	18p11.21	18		tumor necrosis factor superfamily, member 5-induced protei	tumor necrosis factor superfamily, m	GeneID:56984; HGNC:2		GO:5829(cytosol); GO:4		NM_02023	Hs.464652	16251969; protein-coding
218404_at	0,406857	788,9	0,190791	394,95	0,904138	1775,5	1,2193046	2387,5	1,499447	3217,125	1,662095	3378,95	MGC33054	NM_01332	SNX10	7p15.2	7		sorting nexin 10	sorting nexin 10	GeneID:298	GO:7154(cell communic		GO:35091(	NM_01332	Hs.571296	17012226; protein-coding
218253_s_at	2,183228	563	1,272408	328,375	1,027744	275,275	0,8245373	220,125	0,684049	179,825	0,623909	162,8	HCA56	NM_00689	LGTN	1q31-q32	1		ligatin	ligatin	GeneID:193	GO:6886(in	GO:5737(c	GO:3723(R	NM_00689	Hs.497581	15302935; protein-coding
218197_s_at	1,649424	64,175	1,978157	87,575	0,961579	36,825	0,46277636	17,025	1,108223	42,4	0,70159	28,15	OXR1	NM_018002		8q23	8										
218194 at	1.457716	644.6751	1.112286	496.4	0.783346	359	0.618314	301.35	0.95289	433.8	1.042655	479.225	RFN; SFN; CGI-114; MGC	NM 01552	REXO2	11q23.1-q2	11		REX2, RNA exonuclease 2 homolog (S. cerevisiae)	small fragment nuclease	GeneID:259	GO:9117(n	GO:5622(in	GO:8408(3	NM 01552	Hs.7527	16381901; protein-coding
217947_at	0,852728	7830,7	0,481903	4545,825	1,11444	10353,1	0,99314785	9061,15	1,044062	9503,35	0,891311	8252,45	CKLFSF6; PRO2219; FL	NM_01780	CMTM6	3p22.3	3		CKLF-like MARVEL transmembrane domain containing 6	CKLF-like MARVEL transmembrane d	GeneID:549	GO:6935(c	GO:5615(e	GO:5125(c	NM_01780	Hs.380627	15489334; protein-coding
217923_at	0,825329	413,6	0,960625	474,875	0,957776	481,075	0,910186	445,175	1,630052	801,225	1,263633	629,875	PEF1A; PEFLIN	NM_01239	PEF1	1p34	1		penta-EF-hand domain containing 1	penta-EF hand domain containing 1	GeneID:553115; HGNC:		GO:16020(	GO:5509(c	NM_01239	Hs.470417	16429158; protein-coding
217868_s_at	1,251388	1220,475	0,609278	641,575	0,913226	894,975	0,7194152	828,4	1,175657	1179,45	1,340607	1467,5	DREV; DREV1; CGI-81; F	NM_01602	METTL9	16p13-p12	16		methyltransferase like 9	methyltransferase like 9 isoform 1	GeneID:51108; HGNC:24586; HPRD:HPRD_1683				NM_01602	Hs.279583	16730941; protein-coding
217846_at	3,371622	1952,125	1,195846	697,075	1,101566	685,4	0,57095355	355,15	0,936877	551,075	0,525022	308,675	GLNRS; PRO2195	NM_00505	QARS	3p21.3-p21	3	6.1.1.18	glutaminyl-tRNA synthetase	glutaminyl-tRNA synthetase	GeneID:585	GO:6425(g	GO:5737(c	GO:5524(A	NM_00505	Hs.79322	16189514; protein-coding
217750 s at	0.282745	1036.5	0.358533	1304.7	1.362281	5094.6	1.3247617	4898.75	1.027651	3732.45	0.950892	3474.575	HOYS7; FLJ13855	NM 02307	UBE2Z	17q21.32	17		ubiquitin-conjugating enzyme E2Z (putative)	ubiquitin-conjugating enzyme E2Z (p	GeneID:652	GO:6464(p	GO:5634(n	GO:16874(	NM 02307	Hs.514297	16189514; protein-coding
217599_s_at	0,770255	117,325	0,601441	88,12501	2,152794	325,85	1,7084352	254,275	1,284066	185,175	0,645274	92,60001	HIC	BE910600		7q31.2	7		I-mfa domain-containing protein							Hs.132739	
217591_at	0,406265	6,825	0,415017	8,950001	2,183646	43,025	1,7322153	28,8	1,402282	22,725	0,115992	1,925	SKIL	BF725121		3q26	3		SKI-like							Hs.272108	
217553_at	0,343596	19,675	0,410183	26,075	2,812033	182,625	1,4048793	98,375	1,74843	111,875	1,143	74,7	MGC87042	AW129021		7p15.3	7		similar to Six transmembrane epithelial antigen of prostate							Hs.448528	
217504_at	1,195541	297,175	1,792878	418,275	1,44041	328,6	1,436799	342,75	0,316972	76,125	0,236231	56,57501	ABCA6	AA099357		17q24.3	17		ATP-binding cassette, sub-family A (ABC1), member 6							Hs.15780	
217408 at	1.297826	456.8	2.145482	753.4	0.477514	174.65	0.6685257	238.825	1.04394	373.75	1.170932	432.875	PTD017; S18amt; C6orf	AL050361	MRPS18B	6p21.3	6		mitochondrial ribosomal protein S18B	mitochondrial ribosomal protein S18	DAOB-47P1	GO:6412(tr	GO:5622(in	GO:3735(s	NM 01404	Hs.655329	16702430; protein-coding
217388_s_at	0,180194	1194,575	0,283998	1879,675	1,48424	10245,78	1,6523952	11138	1,037666	6852,85	0,985647	6502,725	KYNU	D55639		2q22.3	2		kynureninase (L-kynurenine hydrolase)							Hs.444471	
217028_at	0,559546	1192,475	0,808814	1732,1	0,403208	948,3749	0,6377703	1516	1,847236	3652,55	2,160519	4336,35	CXCR4	AJ224869		2q21	2		Homo sapiens CXCR4 gene encoding receptor CXCR4.		GI:3059120; TrEMBL:O60835						
216834_at	0,088773	459,35	0,318386	1192,775	0,821482	2978,125	1,3205818	4394,25	3,237311	9923,5	3,152705	9772,2	1R20; BL34; IER1; IR20	S59049	RGS1	1q31	1		regulator of G-protein signalling 1	regulator of G-protein signalling 1	GeneID:599	GO:7193(G	GO:5886(p	GO:5096(G	NM_00292	Hs.75256	16710414; protein-coding
216693 x at	1.039281	21.925	0.73391	16.925	0.84968	21.175	1.1221484	24.95	2.783837	54.725	0.988176	21.05	HDGFRP3	AL133102		15q11.2	15		hepatoma-derived growth factor, related protein 3							Hs.127842	
216550 x at	0.826483	180.225	1.969363	435.175	0.462617	97.85001	1.0390952	230.55	1.109273	248.9	1.440737	321.175	ANKRD12	X80821		18p11.22	18		ankyrin repeat domain 12	ribosomal protein L18a homologue	GI:527580; TrEMBL:O15371					Hs.388877	
216348_at	1,579659	228,375	0,636329	92,15	1,271922	180,575	0,80077696	115,325	1,156952	162,975	0,576671	82,52499	RPS17	AL049693		15q	15										
216342_x_at	1,356658	4683,2	0,669394	2369,725	1,306717	4486,1	1,0392591	3592,125	0,863402	2977,1	0,546664	1909,3	RPS4X	AL121916		xq13.1	23										
215773_x_at	1,032682	107,775	1,943184	205,3	0,574253	62,20001	0,7021883	76,775	0,888264	93,525	1,114538	116,7	ADPRT2; PARP-2; ADPR	AJ236912	PARP2	14q11.2-q1	14	2.4.2.30	poly (ADP-ribose) polymerase family, member 2	poly (ADP-ribose) polymerase family	GeneID:100	GO:6284(b	GO:5634(n	GO:3677(D	NM_00548	Hs.409412	16461352; protein-coding
215165 x at	1.080297	323.525	1.710173	510.75	0.589058	184.15	0.61724186	193.6	0.913329	274.6	1.214868	364.325	OPRT	AL080099	UMPS	3q13	3	4.1.1.23	uridine monophosphate synthetase (orotate phosphoribosyl	uridine mon Oroticaciduria	GeneID:737	GO:6207('de novo' pyri		GO:16829(	NM 00037	Hs.2057	17237621; protein-coding
215089 s at	0.935633	124.8	0.907522	125.325	0.683392	90.825	1.0351983	143.475	1.630612	218.275	1.319895	189.375	RBM10	AW409974		xp11.23	23		RNA binding motif protein 10							Hs.348276	
214830_at	1,230644	1518,45	1,34274	1680,65	1,170102	1463,175	0,8772976	1085,575	0,143966	206,825	0,096875	141,1	SLC38A6	AI537540		14q23.1	14		solute carrier family 38, member 6							Hs.200738	
214789_x_at	1,112023	561,05	1,487609	768,45	0,386293	203,1	0,505035	278,975	0,762096	386,125	1,33138	689,1	SRP46	AA524274		11q22	11		Splicing factor, arginine/serine-rich, 46kD							Hs.155160	
214751_at	0,957112	117,55	1,262393	158,275	0,960478	117,9	0,681284	83,725	1,351241	169,7	1,317011	168	LOC90333	BE541042		19q13.42	19		601064390F1 NIH_MGC_10 Homo sapiens cDNA clone IMAGE:3450599 5', mRNA sequence.								
214749 s at	0.947904	2921.8	0.754123	2342.625	0.904559	2849.025	0.8885845	2845.125	1.211108	3777.725	1.230725	3992.05	FLJ20811	AK000818	ARMCX6	Xq21.33-q2	23		armadillo repeat containing, X-linked 6	armadillo repeat containing, X-linked	RP4-545K15.1		GO:16021(integral to m		NM 00100	Hs.83530	17081983; protein-coding
214560 at	0.460901	160.5	0.672547	251.5	2.290925	827.325	1.9249024	721.825	1.242877	513.8	0.63453	226.5	FMLPY; FPRH1; FPRH2; F	NM 00203	FPRL2	19q13.3-q1	19		formyl peptide receptor-like 2	formyl peptide receptor-like 2	GeneID:235	GO:7186(G	GO:16021(i	GO:4982(N	NM 00203	Hs.445466	16002663; protein-coding
214351_x_at	2,306254	7844,45	0,547519	1908,35	1,812374	6212,5	0,78685695	2729,425	1,165969	3986,15	0,243485	949,875	RPL13	AA789278		16q24.3	16		ribosomal protein L13							Hs.410817	
214280_x_at	1,099928	915,7	1,119237	938,9	0,701163	583,175	0,99860287	849,05	1,369816	1152,5	1,027535	865,925	HNRNPA1; MGC102835	X79536	HNRPA1	12q13.1	12		heterogeneous nuclear ribonucleoprotein A1	heterogeneous nuclear ribonucleopr	GeneID:317	GO:6405(R	GO:5737(c	GO:3676(n	NM_00213	Hs.693600	17229681; protein-coding
214221_at	2,444457	147,775	3,908022	223,725	0,846094	46,2	0,89872545	50,2	0,852446	46,5	0,988979	55,4	ALMS1	AI825212		2p13	2		Alstrom syndrome 1							Hs.97393	
214116 at	0.777731	48.975	1.332082	86.375	0.941242	59.425	1.0740229	67.725	0.762448	48.8	1.354403	88.325	BTD	AI767414		3p25	3		biotinidase							Hs.78885	
213982 s at	0.138023	512.95	0.209932	788.15	1.171296	4399.95	1.0620972	4252.9	1.509409	5658.525	1.464912	5712.075	HHL	BG107203		1q24	1		expressed in hematopoietic cells, heart, liver							Hs.242271	
213941_x_at	1,16405	8788,95	0,733349	5625,625	1,104706	8323,225	1,1028398	8298,925	0,834139	6279,15	0,698263	5322,1	RPS7	AI970731		2p25	2		ribosomal protein S7							Hs.444012	
213859_x_at	0,390529	119,925	0,843506	255,1	1,518925	481,475	2,1822927	691,7	0,849879	250,65	1,019383	297,45	SMARCA5	AI652586		4q31.1-q31	4		SWI/SNF related, matrix associated, actin dependent regulator of chromatin, subfamily a, member 5							Hs.135705	
213588_x_at	1,175362	8524,676	0,737121	5436,075	1,071558	7777,025	1,0723349	7777,525	0,917748	6684,2	0,731343	5401,05	RPL14	AA838274		3p22-p21.	3		ribosomal protein L14							Hs.446522	
213374 x at	1.133705	264.975	2.435096	567.575	0.779168	198.3	1.0820035	272.2	0.564163	133.125	0.918851	214.075	HIBCH	AW000964		2q32.3	2		3-hydroxyisobutyryl-Coenzyme A hydrolase							Hs.236642	
213292 s at	1.83453	196.975	2.011986	216.425	1.062928	117.7	1.1768913	129.875	0.43479	49.425	0.742137	81.35001	SNX13	AA908770		7p21.1	7		sorting nexin 13							Hs.269881	
213080_x_at	1,236759	11959,43	0,841201	8339,55	0,982112	9572,975	1,1306888	11103,65	0,886515	8608,6	0,830548	8126,875	RPL5	BF214492		1p22.1	1		ribosomal protein L5							Hs.469653	
212973_at	1,489311	474,45	1,095296	353,575	0,936109	294,15	0,674651	216,5	1,055282	333,85	1,134906	364,55	RPIA	AI692341		2p11.2	2		ribose 5-phosphate isomerase A (ribose 5-phosphate epimerase)							Hs.79886	
212967_x_at	1,472141	3397,2	0,810764	1882,025	1,04326	2415,45	1,0631788	2490,375	1,068339	2468,325	0,70237	1624,75	NAP1L1	AW148801		12q21.1	12		nucleosome assembly protein 1-like 1							Hs.419776	
212930 at	1.895195	319.275	1.661284	280.3	1.071571	193.675	1.1514853	200.05	0.309549	50.4	0.377102	63.2	ATP2B1	AW576457		12q21.3	12		ATPase, Ca++ transporting, plasma membrane 1							Hs.20952	
212794 s at	0.588056	623.575	1.041727	1194.4	1.371374	1409.825	2.092285	2474.7	0.659783	677.125	0.641712	776.975	KIAA1033	AK001728	KIAA1033	12q24.11	12		KIAA1033	hypothetical protein LOC23325	GeneID:23325; HGNC:29174; HPRD:HPRD_1382				NM 01527	Hs.12144	15489334; protein-coding
212785_s_at	0,606457	273,325	1,071424	482,3	0,86879	392,275	0,97103995	438,575	1,16067	515,625	1,04242	471,8	HDCMA18P	AA160181		4q26	4		HDCMA18P protein							Hs.278635	
212783_at	0,690761	90,89999	0,96119	126,275	1,700845	228,825	2,0560184	267,6	1,021087	131,7	0,703676	94,35	RBBP6	AI538172		16p12.2	16		retinoblastoma binding protein 6							Hs.188553	
212702_s_at	2,054293	282	2,171982	308,525	0,692036	97,35001	0,95552427	131,85	0,680401	94,25	0,968738	136,65	BICD2	N45111		9q22.32	9		bicaudal D homolog 2 (Drosophila)							Hs.436939	
212591 at	0.669104	341.075	1.058908	542.15	0.707363	365	1.0413388	532.8	1.51726	778.25	1.823912	933.1751	KIAA0117	AA887480		1q42.3	1		Transcribed sequence with strong similarity to protein sp:P42696 (H.sapiens) Y117_HUMAN Hypothetical RNA-binding protein KIAA0117							Hs.418070	
212568_s_at	0,623771	114,35	1,000101	177,725	0,93331	199,25	0,8306124	153,075	1,666973	302,475	1,282734	229,075	DLAT	BF978872		11q23.1	11		dihydrolipoamide S-acetyltransferase (E2 component of pyruvate dehydrogenase complex)							Hs.115285	
212539_at	1,104729	602,475	1,757858	905,05	0,910388	491,35	0,96720415	514,4	0,488388	246,7	0,488014	235,125	CHD1L	AI422099		1q12	1		chromodomain helicase DNA binding protein 1-like							Hs.429920	
212508_at	2,425951	479,45	2,661376	523,275	0,577938	113,825	0,7550204	149,525	1,130451	225,3	0,990054	199,375	MAP-1; PNMA4	AK024029	MOAP1	14q32	14		modulator of apoptosis 1	modulator of apoptosis 1	GeneID:641	GO:6915(a	GO:5737(c	GO:5515(p	NM_02215	Hs.24719	16713569; protein-coding
212462_at	1,133562	127,325	0,999441	112,55	0,569886	63,55	0,3645466	41,525	1,142283	127,625	1,365677	153,05	MYST4	AU144267		10q22.2	10		MYST histone acetyltransferase (monocytic leukemia) 4							Hs.27590	
212446 s at	0.554738	421.4	0.800603	605.85	0.948257	765.5	1.0343351	807.1	1.578613	1191.525	1.850978	1424.875	LASS6	AI658534		2q31.1	2		hypothetical protein LOC253782							Hs.503941	
212335_at	0,504493	484,325	0,602415	592,725	0,749102	784,95	0,86935353	936,475	1,902546	1728,975	1,62463	1546,975	GNS	AW167793		12q14	12		glucosamine (N-acetyl)-6-sulfatase (Sanfilippo disease IIID)							Hs.334534	
212268_at	0,25156	632,225	0,367021	919,9251	1,277321	3794,475	1,9105958	5399,875	1,343241	3462,9	1,400056	3625,05	EI; LEI; PI2; MNEI; M/NEI;	NM_03066	SERPINB1	6p25	6		serpin peptidase inhibitor, clade B (ovalbumin), member 1	serine (or cysteine) proteinase inhibi	GeneID:1992; HGNC:33		GO:5737(c	GO:4867(s	NM_03066	Hs.381167	15952740; protein-coding
212247_at	0,656275	79,475	1,079532	130,65	0,963833	119,425	1,3592091	169,95	0,93367	114,475	0,97212	124,725	NUP205	AW008531		7q33	7		nucleoporin 205kDa							Hs.413636	
212243_at	1,654516	360,95	0,892146	197	0,878565	196,45	0,6198369	135,025	1,090015	237,175	0,922967	210,375	GRINL1A	BE645501		15q22.1	15		glutamate receptor, ionotropic, N-methyl D-aspartate-like 1A							Hs.201495	
212235 at	2.102173	152.65	1.372114	103.825	0.786556	59.75	1.0980203	86.4	0.500451	37.7	0.868878	66.025	PLXND1	AL575403		3q21.3	3		plexin D1							Hs.301685	
212215_at	2,321803	301,825	3,648718	475,75	0,907423	124,125	0,7570521	104,6	0,378988	48,475	0,588839	77,175	KIAA0436	AB007896		2p22.1	2		putative L-type neutral amino acid transporter		GI:266215	proteolysis and peptidol		catalytic activity; prolyl		Hs.110	
212175_s_at	0,892203	634,025	1,458623	1030	0,633675	444,75	0,8286107	582,025	1,024597	709,35	1,401977	990,975	AK2	AL513611		1p34	1		adenylate kinase 2							Hs.294008	
212159_x_at	4,851134	1055,825	6,410391	1366,6	1,019424	203,45	0,9209139	187,8	0,792505	159,125	0,551077	110,3	AP2A2	AI125280		11p15.5	11		adaptor-related protein complex 2, alpha 2 subunit							Hs.19121	
212052_s_at	1,553304	741,95	1,127397	541,775	0,640528	326,925	0,638856	312,225	0,927339	443,525	1,118017	546,7	FLJ41880; KIAA0676;	AB014576	TBC1D9B	5q35.3	5		TBC1 domain family, member 9B (with GRAM domain)	TBC1 domain family, member 9B (wi	GeneID:230	GO:32313(	GO:5622(in	GO:5097(R	NM_01504	Hs.155829	16303743; protein-coding
212039 x at	1.41454	11883.05	0.77723	6640.45	1.201482	10120.9	1.0957096	9211.125	0.785139	6585.15	0.367768	3142.2	RPL3	BG339228		22q13	22		ribosomal protein L3							Hs.119598	
211938_at	3,597041	1111,175	1,836161	574	0,946384	300,675	0,8138216	264,425	0,56914	182,05	0,493754	156,05	EIF4B	BF247371		12q13.13	12		eukaryotic translation initiation factor 4B							Hs.93379	
211754_s_at	1,04128	115,725	2,040602	226,075	0,91963	103,8	0,6846458	94,875	0,71827	77,075	1,221747	132,975	PMP34	BC005957	SLC25A17	22q13.2	22		solute carrier family 25 (mitochondrial carrier; peroxisomal m	solute carrier family 25 (mitochondri	GeneID:104	GO:6839(m	GO:5887(in	GO:5386(c	NM_00635	Hs.474938	15489334; protein-coding
211668_s_at	2,820938	44,125	1,875101	33,375	0,564684	11,05	0,72971773	13,3	0,251687	7,025	0,555158	10,9	ATF; UPA; URK; u-PA	K03226	PLAU	10q24	10	3.4.21.31	plasminogen activator, urokinase	urokinase p Alzheimer disease, late-	GeneID:532	GO:7596(b	GO:5615(e	GO:16301(	NM_00265	Hs.77274	17264329; protein-coding
211454 x at	0.750074	96.625	0.584313	76.52499	1.183917	154.35	1.3715041	175.375	0.832885	105.05	1.630153	218.45		AF336878			0		similar to other members in this gene family FKSG48 to FKS	FKSG51	GI:13384185						
211406 at	1.661069	96.125	0.630261	36.2	1.249782	75.05	0.81415457	47.05	1.258768	73.025	0.872049	49.5	HSPC039; PRO2309	AF119875	IER3IP1	18q12	18		immediate early response 3 interacting protein 1	immediate early response 3 interacti	GeneID:51124; HGNC:1		GO:5783(endoplasmic r		NM 01609	Hs.653122	16341674; protein-coding
211275_s_at	1,307401	3129,625	1,2943	3044,975	0,540069	1426,175	0,59087914	1563,225	0,82926	1967,075	1,111875	2647,975	GYG	AF087942	GYG1	3q24-q25.1	3		glycogenin 1	glycogenin self-glucosy	GeneID:299	GO:16051(carbohydrate		GO:8466(g	NM_00413	Hs.477892	17055998; protein-coding
211150_s_at	0,594329	55,85	0,981366	90,025	0,638053	66,15	0,8406419	78,975	1,800261	169,625	1,297349	118,775	DLAT	J03866		11q23.1	11	2.3.1.12	Homo sapiens M2 mitochondrial autoantigen dihydrolipoamic	dihydrolipoamide acetyltransferase	GI:619444						
211144_x_at	0,246078	17,025	0,41742	25,05	1,030036	65,4	0,92366236	58,55	6,616107	393,05	5,360167	317,85	TRGV9	M30894		7p15	7		T cell receptor gamma locus		GI:339407; GDB:G00-119-765					Hs.385086	
211138 s at	0.801941	681.6	0.948194	804.35	5.225483	4439.05	4.8961577	4167.2	0.700853	750.75	0.356879	374.675	dJ317G22.1	BC005297	KMO	1q42-q44	1	1.14.13.9	kynurenine 3-monooxygenase (kynurenine 3-hydroxylase)	kynurenine 3-monooxyg monooxyge	GeneID:856	GO:6118(e	GO:16021(i	GO:9055(e	NM 00367	Hs.409081	16716206; protein-coding
211063 s at	0.447936	831.225	1.118536	2003.55	0.45168	817.325	0.90118074	1639.65	1.343483	2436.8	2.050284	3772.9	NCK; MGC12668; NCKa	BC006403	NCK1	3q21	3		NCK adaptor protein 1	NCK adaptor protein 1	GeneID:469	GO:42110(	GO:5737(c	GO:8093(c	NM 00615	Hs.477693	16835242; protein-coding
210825_s_at	1,244185	2978,775	0,960293	2295,45	0,792653	1977,525	0,7051289	1738,5	0,938326	2313,85	1,056198	2618,05	STOM	AF130103		9q34.1	9		predicted protein of HQ0720; Homo sapiens clone FLB2914	PRO0720	GI:11493510						
210663_s_at	0,167847	152,525	0,300237	274,625	3,168431	3168,3	3,5684602	3845,075	1,236397	1142,65	0,812089	834,15	KYNU	BC000879	KYNU	2q22.2	2	3.7.1.3	kynureninase (L-kynurenine hydrolase)	kynureninase (L-kynurenine hydrolas	GeneID:894	GO:9435(N	GO:5737(c	GO:16787(	NM_00103	Hs.470126	12477932; protein-coding
210570_x_at	1,775868	122,025	1,281346	90,675	1,408577	99,85	0,60203314	46,425	0,861877	59,575	0,528442	40,625	JNK2; JNK2A; JNK2B; P	U35002	MAPK9	5q35	5	2.7.1.-	mitogen-activated protein kinase 9	mitogen-activated protein kinase 9 i	GeneID:560	GO:7254(J	nucleus	GO:5524(A	NM_00275	Hs.654461	17296730; protein-coding
210104 at	0.802585	65	1.218204	101.175	0.964205	77.7	1.1860567	102.3	1.504894	121.25	0.747056	67.32501	NY-REN-28	AF074723	MED6	14q24.2	14		mediator of RNA polymerase II transcription, subunit 6 homo	mediator of RNA polymerase II trans	GeneID:100	GO:45944(	GO:119(me	GO:3702(R	NM 00546	Hs.497353	15808511; protein-coding
210029 at	0.01	21.875	0.01	35.45	0.975005	6709.625	1.173211	7922.774	1.858612	11144.87	1.575858	9518.649	IDO; CD107B	M34455	INDO	8p12-p11	8	1.13.11.42	indoleamine-pyrrole 2,3 dioxygenase	indoleamine-pyrrole 2,3 dioxygenase	GeneID:362	GO:6955(immune respo		GO:9055(e	NM 00216	Hs.840	17393386; protein-coding
210028_s_at	0,845132	92,825	1,184616	128,725	0,835387	89,65	0,5849229	62,675	1,506146	160,9	1,269741	136,525	LAT; ORC3; LATHEO	AF125507	ORC3L	6q14.3-q16	6		origin recognition complex, subunit 3-like (yeast)	origin recognition complex, subunit 3	GeneID:235	GO:6260(D	GO:5664(n	GO:3677(D	NM_01238	Hs.410228	15910003; protein-coding
209705_at	1,161992	214,725	1,286202	238,85	0,754825	143,35	0,722674	134,675	0,999431	186,925	0,91899	170,275	M96	BG033764		1p22.1	1		likely ortholog of mouse metal response element binding transcription factor 2							Hs.31016	
209678_s_at	0,886767	221,975	1,222562	335,575	0,94828	242,6	0,9301282	262,6	0,95303	236,9	0,888197	256,9	PKCI; DXS1179E; MGC2	L18964	PRKCI	3q26.3	3	2.7.11.1	protein kinase C, iota	protein kinase C, iota	GeneID:558	GO:7015(a	GO:45177(	GO:5524(A	NM_00274	Hs.478199	17133348; protein-coding
209606 at	0.262968	1157.9	0.424713	1846.85	0.848217	3688.2	1.1487485	4978.95	1.675196	7223.6	1.812725	7845.8	HE; B3-1; CASP; CYBR;	L06633	PSCDBP	2q11.2	2		pleckstrin homology, Sec7 and coiled-coil domains, binding p	pleckstrin homology, Sec7 and coilec	GeneID:959	GO:30155(	GO:5938(c	GO:5515(p	NM 00428	Hs.270	16702224; protein-coding
209536 s at	0.459387	915.925	0.738923	1472.95	1.251857	2489	1.8470404	3666.7	0.910319	1784.8	1.108786	2176.375	EHD4	AF320070		15q11.1	15		Homo sapiens hepatocellular carcinoma-associated protein H	hepatocellular carcinoma-associated	GI:19113673						
209367_at	1,188188	95,2	1,278317	101,825	0,870871	70	0,76154524	59,725	1,353111	107,025	0,738132	59,15	UNC18B; Hunc18b; UNC	AB002559	STXBP2	19p13.3-p1	19		syntaxin binding protein 2	syntaxin binding protein placuble syr	GeneID:681	GO:6886(in	GO:5575(c	GO:30347(	NM_00694	Hs.515104	12773094; protein-coding
209265_s_at	0,784338	253,95	1,508135	487,025	0,574371	179,025	0,8128853	253,275	1,423856	459,95	1,754665	558,325	M6A; Spo8; MT-A70; M	BC001650	METTL3	14q11.1	14	2.1.1.62	methyltransferase like 3	methyltransferase like 3	GeneID:563	GO:1510(R	GO:5634(n	GO:3723(R	NM_01985	Hs.168799	16964243; protein-coding
209056_s_at	0,769809	633,175	1,152079	947,075	0,620835	509,8	0,9463224	775,15	1,054466	864,5	1,187017	975,5	CEF1; hCDC5; PCDC5RP;	NM_00125	CDC5L	6p21	6		CDC5 cell division cycle 5-like (S. pombe)	CDC5-like	RP1-319D2	GO:8380(R	GO:5634(n	GO:3677(D	NM_00125	Hs.485471	17353931; protein-coding
209030 s at	0.819395	24.9	1.042921	29.3	1.011597	26.475	0.5582288	16.8	1.613748	51.175	1.297865	54.2	BL2; ST17; IGSF4; NECL	NM 01433	CADM1	11q23.2	11		cell adhesion molecule 1	immunoglobulin superfamily, member	GeneID:237	GO:1913(T	GO:16323(	GO:30165(	NM 01433	Hs.370510	17130425; protein-coding
208974 x at	0.539143	1345.9	0.594229	1544.45	1.054134	2623.525	1.4206374	4087.25	1.383718	3411.625	1.119243	3100.45	IMB1; IPOB; Impnb; NTF	BC003572	KPNB1	17q21.32	17		karyopherin (importin) beta 1	karyopherin beta 1	GeneID:383	GO:6607(N	GO:5737(c	GO:8139(n	NM 00226	Hs.532793	16982803; protein-coding
208828_at	2,42049	838,75	1,917414	733,1	0,883649	338,05	0,45409256	166,4	1,027679	356,275	0,494333	195,575	p17; YBL1; CHRAC17; C	BC004170	POLE3	9q33	9		polymerase (DNA directed), epsilon 3 (p17 subunit)	DNA polymerase epsilon subunit 3	GeneID:541	GO:6260(D	GO:5622(in	GO:3893(e	NM_01744	Hs.108112	17081983; protein-coding
208805_at	0,597635	3008,175	0,91157	4614,8	1,823556	9353,725	1,8561027	9448,25	1,041956	5254,275	0,941298	4739,575	IOTA; p27K; PROS27; M	BC002979	PSMA6	14q13	14	3.4.25.1	proteasome (prosome, macropain) subunit, alpha type, 6	proteasome Myocardial infarcation, s	GeneID:568	GO:6511(u	GO:5829(c	GO:3723(R	NM_00279	Hs.446260	16845397; protein-coding
208765_s_at	1,042778	578,825	1,586394	940,475	0,636035	369,1	0,8563401	484,825	1,06905	622,75	0,856351	510,45	HNRNPR; hnRNP-R; FLJ2	NM_00582	HNRPR	1p36.12	1		heterogeneous nuclear ribonucleoprotein R	heterogeneous nuclear ribonucleopr	GeneID:102	GO:8380(R	GO:30530(	GO:3723(R	NM_00582	Hs.373763	17353931; protein-coding

208758_at	1.860893	670.95	2.153297	778.225	0.634224	245.45	0.7100556	272.625	0.958754	350.1	1.015726	373.175	PURH; AICAR; AICARFT;	D89976	ATIC
208752_x_at	1.547315	3253.4	0.812	1719.25	1.046004	2221.825	1.1131637	2374	1.063038	2246.2	0.70123	1476.175	NAP1L1	AI888672	
208714_at	1.271797	1377.65	1.6392	1753.425	1.045734	1128.65	1.1149801	1229.975	0.495686	528.2	0.285655	331.2	NDUFV1	AF092131	
208485_x_at	0.174335	123.025	0.182849	128.825	1.646327	1197.375	1.1610004	978.225	1.698576	1201.55	0.774258	611.0501	CASH; FLIP; MRIT; CLARI	NM_00387	CFLAR
208319_s_at	0.725142	364.3	0.805745	377.325	1.325739	619.55	1.7209873	861.075	1.001294	467.925	0.667853	321.875	RNPL; IS1-RNPL	NM_00674	RBM3
207891_s_at	1.511956	37.1	1.468673	35.225	0.785799	22.075	0.711882	16.525	1.292704	30.825	0.291504	10.85	TREX2	NM_017518	
207850_at	0.031666	10.1	0.064114	20.525	3.442125	1050.75	3.4673138	975.825	0.675517	223.35	1.564562	423.175	GRO3; GROg; MIP2B; SC	NM_00209	CXCL3
207777_s_at	0.237504	31.025	0.15328	21.425	0.90424	122.15	1.1368346	157.875	1.72625	219.225	1.786208	233.475	LYSP100-A; LYSP100-B	NM_00723	SP140
207618_s_at	1.304973	108.05	1.841254	152.475	0.804475	66.625	0.7096479	61.275	0.945429	78.275	0.987588	81.775	BCS; BJS; PTD; BCS1; FL	NM_00432	BCS1L
207505_at	1.173856	39.075	0.999853	32.3	0.91334	29.975	1.4135771	47.275	0.612994	20.825	1.180214	40.05	cGKII; PRKGR2	NM_00625	PRKG2
206928_at	1.106966	183.325	1.148083	215.4	1.257146	197.375	0.86455667	154.575	1.006287	153.6	1.079385	186.3	HZF16; HZF-16; MGC11	NM_00343	ZNF124
206760_s_at	1.26249	334.05	1.61374	413.8	2.025343	477.725	1.4501027	370.7	0.387114	86.3	0.138386	35	CD23; FCE2; CD23A; IGI	NM_00200	FCER2
206687_s_at	1.787573	685.25	1.955534	743.5	0.813491	342.375	1.0347012	425.5	0.378351	159.5	0.314588	134.025	HCP; HCPH; SHP1; SHP-1	NM_00283	PTPN6
206589_at	0.26722	9.125001	1.010821	32	0.332383	13.825	0.24905296	14.225	1.948892	65.375	3.839486	120.6	ZNF163	NM_00526	GFI1
206584_at	1.112	1983.75	1.256426	2256.575	1.221972	2179.725	0.9187768	1634.275	0.531908	1032.1	0.267554	549.5	MD-2	NM_01536	LY96
206555_s_at	0.985142	561.4251	1.23129	698.2	0.506997	286.475	0.83675075	470.875	0.965812	539.175	0.926787	568.4	DKFZp686C1054	NM_01773	THUMPD1
206544_x_at	1.802828	55.225	2.850768	90.45	0.823567	25.925	0.29108426	10.8	1.079177	31.8	0.652215	20.65	BRM; SNF2; SWI2; hBRM	NM_00307	SMARCA2
206521_s_at	1.220475	60.475	0.824697	42.15	0.462284	26.3	0.881836	45.55	1.2092	59.2	1.025025	50.8	TF2A1; TFIIA; MGC1299	NM_01585	GTF2A1
206332_s_at	0.688741	1152.575	1.091204	1814.1	1.040165	1709.725	1.4125506	2371.825	0.977407	1591.1	0.891407	1519.175	PYHIN2; IFNGIP1; MGC9	NM_00553	IFI16
205849_s_at	1.270554	4532.625	1.018031	3652.325	1.080312	3838.3	1.0194943	3664.5	0.475824	1754.2	0.276328	1033.95	OPC; QP-C; UQBC; UQBP	NM_00629	UQCRB
205804_s_at	0.397963	47.225	0.679369	83.825	2.828377	491.25	2.760993	595.75	1.146279	130.45	0.806287	91.825	T3JAM; FLJ44151; MGC	NM_02522	TRAF3IP3
205419_at	0.261125	566.4	0.354077	756.325	0.932181	2487.125	0.93322915	2462.3	2.117636	4142.3	1.537564	3013.9	EBI2	NM_00495	EBI2
205306_x_at	0.76531	558.425	0.913377	662.45	5.525697	4057.125	5.0709586	3734.575	0.73386	676.375	0.398631	370.575	KMO	AI074145	
205298_s_at	1.287296	300.025	2.203776	511.175	0.629883	141.45	0.7357883	165.3	1.19985	276.6	1.599026	369.8	BTN2A2	W58757	
205269_at	0.651632	457.65	0.96638	664.975	2.446063	1843.7	3.0512905	2258.125	0.484974	389.95	0.491683	409.975	LCP2	AI123251	
205207_at	0.026616	35.75	0.014038	19.675	6.365073	10551.55	7.065552	10501.25	0.871973	1269.975	0.957062	1394.85	HGF; HSF; BSF2; IL-6; IFI	NM_00060	IL6
205191_at	0.35152	112.975	0.963226	298.4	1.505558	523.125	2.1189997	744.675	0.814825	254.275	1.229242	382.05	TBCCD2; KIAA0215	NM_00691	RP2
205077_s_at	1.530088	411.875	1.004346	278.95	0.943123	248	0.75740033	207.3	1.161261	311.975	1.136847	306	MGC32646; MGC33136	NM_00264	PIGF
205050_s_at	1.354406	59.825	0.472269	22.125	1.264739	54.775	0.8858244	39.125	1.100037	47.15	0.68878	32.2	IB2; JIP2; PRKM8IPL	NM_01232	MAPK8IP2
205042_at	0.796481	120.425	0.79382	120.575	0.6128	92.3	1.0208957	153.725	1.560634	234.2	1.644993	247.075	NM; DMRV; IBM2; Uae1;	NM_00547	GNE
204972_at	0.045166	71.47501	0.041993	67.4	1.061441	1706.525	1.4342442	2324.15	1.275823	1961.15	1.238855	1931.925	MGC78578	NM_01681	OAS2
204956_at	1.529562	28.8	1.977478	36.75	0.514999	9.65	0.8542627	16.45	1.014316	19.175	0.90415	16.85	MSAP; c86fus	NM_00245	MTAP
204882_at	0.584245	517.95	0.811851	722.95	0.967439	852.1	1.0171952	905.9	1.301493	1155.85	1.00921	955.475	ARHGAP25	NM_014882	
204861_s_at	4.488409	272.75	4.899955	293.525	1.222331	73.8	0.8237032	49.55	0.160688	14.625	0.358552	23.825	BIRC1; NLRB1; psiNAIP;	NM_00453	NAIP
204794_at	0.313922	137.95	0.30149	125.7	3.956404	2640.25	3.9210749	2731.775	0.967728	394.675	1.275992	527.675	PAC1; PAC-1	NM_00441	DUSP2
204599_s_at	0.918798	246.725	1.025045	266.475	0.774182	204	0.94353527	246.2	1.455918	390.25	1.02198	276.4	p15; MAAT1; MGC8499	NM_00642	MRPL28
204528_s_at	1.393348	1523.55	0.844794	929.65	0.923678	1015.775	1.2878202	1411.425	0.982562	1092.9	0.73688	816.3001	NRP; NAP1; NAP1L; MG	NM_00453	NAP1L1
204520_x_at	0.518082	97.85	1.371638	245.65	0.57607	109.1	0.9395305	171.575	0.900063	162.425	1.468809	279.125	BRL; BRPF1; BRPF2; DKF	NM_01457	BRD1
204517_at	1.508792	807.05	1.052183	603.65	1.27357	749.575	0.9669412	552.2	0.766511	400.225	0.754043	403.075	PPIC	BE962749	
204490_s_at	0.537533	468.575	0.382556	337.925	1.866438	1876.125	2.604909	2665.525	1.082661	957.9	1.051599	965.7499	IN; LHR; MC56; MDU2; M	M24915	CD44
204224_s_at	0.018006	103.825	0.017252	98.675	1.514814	8569.201	1.5908796	8737.3	1.065287	5888.525	1.17465	6385.8	GCH; DYT5; GTPCH1; GT	NM_00016	GCH1
204118_at	0.311864	155.575	0.232325	112.875	1.478685	677.35	1.9265478	889.775	1.088419	547.5	1.000415	539.475	BCM1; BLAST; hCD48; m	NM_00177	CD48
204042_at	1.109061	12.725	0.492297	6.025	1.188873	14.425	0.8350529	10.7	0.368357	5.55	0.8695	15.325	SCAR3; WAVE3; KIAA0	AB020707	WASF3
204026_s_at	1.235149	220.7	3.180899	570.4	0.666688	123.55	0.8506786	160.925	0.490471	87.25	0.896184	163.05	KNTC2AP; HZwint-1; MG	NM_00705	ZWINT
203984_s_at	1.424202	388.775	1.892508	517.325	0.384731	106.35	0.48021212	132.15	0.77668	209.45	1.400907	381.975	MCH6; APAF3; APAF-3;	U60521	CASP9
203922_s_at	0.478361	76.85	0.485643	85.3	1.505498	261.475	1.6359429	326.325	1.888811	358.575	1.175359	219.275	CYBB	AI308863	
203796_s_at	2.346323	188.975	1.044227	84.125	0.353404	38.275	0.4004184	32.975	1.048502	81.975	0.905982	85.775	BCL7A	AI950380	
203778_at	1.690664	220.075	1.239451	171.1	0.188872	36.4	0.5380047	71.4	1.568541	210.575	0.952091	131.775	MANB1	NM_00590	MANBA
203525_s_at	1.027864	166.3	1.326805	211.825	1.011365	166.175	1.0070516	170.15	0.791919	128.075	0.847984	141.7	APC	AI375486	
203516_at	1.697819	59.175	0.617271	21.3	1.754256	56.675	0.6989511	24.275	0.985535	34.95	0.837124	34.65	SNT1; TACIP1; dJ1187J	NM_00309	SNTA1
203341_at	1.047678	285.95	1.893423	534.2	0.79952	217	0.88376015	239.275	1.110866	326.675	1.132583	343.15	CBF2; NOC1; HSP-CBF	NM_00576	CEBPZ
203203_s_at	0.778306	72.375	1.324316	132.55	1.430431	135.275	2.151728	229.85	0.681827	65.85	0.560381	70.475	HRB2; RIP-1	NM_00704	KRR1
203178_at	1.035084	326.45	1.894537	582.2	1.458631	482.8	2.3087149	800.6751	0.094962	28	0.0998	30.2	AT; AGAT	NM_00148	GATM
203067_at	0.776483	433.475	1.380765	773.95	0.678759	379.4	0.8200426	460.45	1.254145	699.275	1.450981	824.75	E3BP; OPDX; PDX1; proX	NM_00347	PDHX
203064_s_at	1.244151	314.025	0.812957	201.95	0.848704	216.35	0.68489593	169.575	1.408806	356.4	1.086097	270.85	ILF; ILF1; ILF-1	NM_00451	FOXK2
203063_at	3.284751	804.575	3.319295	822.1751	0.943212	257.325	0.9556557	262.275	0.206137	50.125	0.129793	32.775	FEM-2; POPX2; hFEM-2;	NM_01463	PPM1F
203060_s_at	1.378419	241.375	1.042481	211.9	0.677065	113.25	0.8969629	143.35	0.771676	124.3	1.359294	227	SK2; ATPSK2	AF074331	PAPSS2
202959_at	1.912136	110.625	1.470148	82.85	0.894672	50.55	0.86296076	48.525	0.720833	41.975	0.995086	56.075	MUT	AI433712	
202869_at	0.082906	106.075	0.124395	161.475	1.086429	1284.075	1.4380615	1723.225	2.019736	2653.35	1.951458	2608.525	OIAS; IFI-4; OIASI	NM_01681	OAS1
202813_at	1.230431	66.525	1.611841	87.575	0.939128	51.15	1.0027459	54.525	0.672617	38.475	0.258127	16.95	TRM3; TRP185; TRP-18	NM_00564	TARBP1
202520_s_at	0.949295	472.85	1.277469	630.125	0.837553	412.875	1.0082263	499.075	0.993686	492.45	0.976903	481.875	FCC2; COCA2; HNPCC; h	NM_00024	MLH1
202494_at	0.956303	145.725	1.38127	208.55	0.734558	114.05	0.93948543	147.15	0.916558	141.525	0.78744	122.45	CYP-33; MGC3736; MGC	NM_00611	PPIE
202447_at	0.804509	1047.225	1.439271	1907.125	1.10236	1441.775	1.6067092	2173.25	0.628957	829.975	0.686481	983.15	DECR; NADPH	NM_00135	DECR1
202347_s_at	1.094799	1770.15	0.729868	1203.05	0.875717	1430.075	0.77958095	1324.625	1.476364	2408.125	1.38137	2312.925	LIG; HYPG; UBE2K	AB022435	HIP2
202307_s_at	0.130582	481.6	0.190698	716.625	1.094882	4317.25	1.2625132	4948.05	1.433786	5264.4	1.289724	4846.3	APT1; PSF1; ABC17; AB	NM_00059	TAP1
202261_at	0.900149	316.3	1.315442	459.825	0.591598	209.925	0.8437379	296.625	1.148161	407.1	1.190191	423.425	YL1; Swc2; YL-1; TCFL1	NM_00599	VPS72
202251_at	0.5939	164	0.641384	176.9	1.006462	280.9	1.1112483	321.45	1.347723	373.2	0.973107	281.8	PRP3; RP18; HPRP3; Prp	NM_00469	PRPF3
202239_at	1.115942	529.9	2.131618	980.1751	0.983781	442.2	1.5346768	688.55	0.585444	261.2	0.593015	274.5	PH5P; p193; PARPL; VP	NM_00643	PARP4
202157_s_at	0.688794	1599.45	1.012249	2358.05	0.78024	1803.025	1.1345271	2651.5	2.548649	5925.95	2.367194	5517.775	ETR-3; NAPOR; BRUNOL	U69546	CUGBP2
202156_s_at	0.697688	295.425	0.882764	371.925	0.915442	388.925	0.9493186	399.2	2.915788	1250.225	2.492724	1073.175	CUGBP2	N36839	
202093_s_at	0.833061	273.525	0.935825	325.025	0.74497	249.75	1.152382	399.475	1.580656	529.5	1.799766	627.85	PD2; F23149_1; FLJ11	NM_01908	PAF1
202068_s_at	0.593287	93.4	0.466436	74	0.903358	137.4	1.1164174	170.2	2.409087	370.9	2.745684	436.1	FH; FHC	NM_00052	LDLR
202042_at	0.60193	402.2	0.852995	572.35	0.700684	491.9	1.220966	858.5	3.320005	2510.15	3.954309	2944.575	HRS; FLJ20491	NM_00210	HARS
202029_x_at	1.420348	9460.775	0.571141	3801.8	1.369815	9077.8	1.0360264	6887.85	0.961338	6353.15	0.449025	3099.375	RPL38	NM_00099	RPL38
201858_s_at	0.334904	1713.025	0.694237	3481.375	1.254275	6934.15	1.6421378	8856.625	1.601573	7771.175	1.316634	6746.875	PPG; PRG; PRG1; MGC92	J03223	SRGN
201850_at	0.46192	821.475	0.735025	1264.825	0.862127	1430.65	1.0834728	1932.675	1.896753	3241.825	1.199196	2344.65	MCP; AFCP	NM_00174	CAPG
201849_at	0.471647	515.8	0.248103	291.15	1.513348	1617.075	1.0234691	1101.975	1.490583	1556.4	1.251674	1340.8	NIP3	NM_00405	BNIP3
201837_s_at	0.786725	199.65	1.25652	319.825	0.682171	173.9	0.889408	226.85	1.235549	312.375	1.184678	303.4	STAF65(gamma)	AF197954	
201830_s_at	0.581681	299.675	1.261378	645.925	0.563649	289.475	0.8961913	452.5	3.234866	1638.025	4.480054	2310.2	NET1A; ARHGEF8	NM_00586	NET1
201825_s_at	1.174476	94.675	1.125803	94.55	0.767833	64.025	0.53711486	45.825	0.956443	76.875	1.421308	115.8	CGI-49	AL572542	
201798_s_at	0.524429	620.075	0.858444	1049.175	1.462567	1647.125	2.255264	2571.8	0.766094	890.55	0.913196	1096.95	MYOF; FLJ36571; FLJ9	NM_01345	FER1L3
201780_s_at	1.14576	1131.875	1.406139	1368.05	1.051165	1026.95	0.9097168	886.575	0.710028	747.7	0.571386	598.2501	RZF; MGC13689	NM_00728	RNF13
201768_s_at	0.516498	88.6	0.769856	134.55	1.260027	223.675	1.4393693	259.5	1.018564	172.15	1.26182	216.15	ENTH; EPN4; EPNR; CLIN	BC004467	CLINT1
201762_s_at	0.281021	1606.025	0.394025	2252.1	0.951923	5420.975	1.1811433	6837.65	1.789055	10186.18	1.496376	8571.925	PA28B; REGbeta; PA28b	NM_00281	PSME2
201752_s_at	1.355822	328.625	1.927873	504.725	0.673254	140.925	0.69512147	160.725	0.676404	148.925	0.678622	161.35	ADD3	AI763123	
201683_x_at	0.788235	190.85	0.73648	207.025	1.026222	253.95	0.9124079	283.025	1.047193	254.4	0.767998	224.275	C14orf92	BE783632	
201641_at	0.496243	952.675	0.429812	853.35	0.947332	1819.6	1.126518	2210.55	2.300319	4441.775	1.815872	3631.775	CD317	NM_00433	BST2
201537_s_at	1.17249	288.7	0.455238	114.475	0.914591	224.8	0.5615544	143.6	1.526765	377.725	1.023342	260.525	VHR	BC002682	DUSP3
201531_at	0.699451	255.175	0.486718	176.125	1.782471	675.35	1.8774208	814.725	0.93355	345.55	1.193814	433.2	TTP; G0S24; GOS24; TIS	NM_00340	ZFP36
201520_s_at	0.701654	983.3	1.019132	1428.125	0.715946	1016.2	1.1340269	1622.35	3.461133	4908.125	3.766629	5343.15	GRSF1	BF034561	
201508_at	0.075844	3.625	0.260267	13.125	1.028991	43.625	0.69308925	31.225	3.751444	168.25	2.508217	119.375	BP-4; IBP4; IGFBP-4; HT	NM_00155	IGFBP4
201460_at	1.061595	164.75	0.566796	87.375	1.179519	184.175	0.8665245	132.975	1.174732	180.4	1.281474	198.15	MAPKAPK2	AI141802	
201408_at	1.027622	279.175	1.330289	364.55	1.06823	294.825	0.924236	254.75	0.666776	193.025	0.695021	202.8	PPP1CB	W67887	
201403_s_at	1.070037	945.55	1.411298	1232.625	0.970914	843.9	1.5910311	1386	0.651384	564.825	0.765735	663.05	GST-III	NM_00452	MGST3
201377_at	1.57119	609.775	1.592005	613.65	0.815448	342.075	0.6049818	243.975	0.811698	323.025	0.587383	233.675	NICE-4; FLJ42300; KIAA	NM_01484	UBAP2L
201359_at	0.73196	422.425	1.179585	689.25	0.862996	509	1.5653397	909.575	0.90241	504	1.031947	604	COPB; FLJ10341; DKFZ	NM_01645	COPB1
201198_s_at	1.235196	493.075	1.548945	627.1751	0.994827	391.25	1.08117	434.225	0.80903	319.7	0.718296	291.85	PSMD1	AI860431	
201146_at	0.503763	1335.625	0.786996	2058.6	1.660994	4564.375	2.1073034	5836.7	0.931254	2484.325	1.00392	2720.425	NRF2	NM_00616	NFE2L2
201076_at	3.578628	846.825	2.09647	500.1	0.855619	213.75	0.6400851	158.9	0.966516	228.45	0.698193	167.025	NHPX; OTK27; SNU13	NM_00500	NHP2L1
201068_s_at	0.859069	2494.45	1.57493	4557.5	0.817804	2408	1.3545777	3946.5	0.873578	2561.525	0.967509	2840.65	S7; MSS1; MGC3004; N	NM_00280	PSMC2
201060_x_at	0.393603	299.25	0.657566	510.775	0.674062	511.2	1.6512817	1301.55	1.804677	1461.15	3.780525	2974.1	STOM	AI537887	
200992_at	1.193225	1042.375	1.228394	1090.6	0.613553	538.825	1.119735	979.975	0.942351	819.7	1.107369	979.4501	IPO7	AL137335	
200963_x_at	1.408751	13361.55	0.66837	6597.75	1.251432	11871.68	1.076232	10215.7	0.815897	7746.275	0.442687	4303.125	MGC88191	NM_00099	RPL31
200959_at	0.580803	746.925	1.324031	1685.575	0.927511	1164.375	1.5196047	1876.6	0.91127	1129.775	1.232727	1633.525	TLS; FUS1; FUS-CHOP	NM_00496	FUS
200933_x_at	1.240959	11865.88	0.690386	6785.15	1.186324	11390.8	1.0855352	10347.82	0.923996	8812.225	0.486721	4684.025	CCG2; SCAR; SCR10; DX	NM_00100	RPS4X
200926_at	1.359163	14539.52	0.691301	7460	1.426431	15357.25	1.1971173	12787.65	0.83907	8971.925	0.436204	4827.85	FLJ35016	NM_00102	RPS23
200912_s_at	1.008855	3559.8	1.312228	4647.15	0.430042	1500.5	0.79696363	2850.8	0.975433	3415.55	1.299054	4683.2	DDX2B; EIF4A; EIF4F; BN	NM_00196	EIF4A2
200861_at	1.498234	96.25	1.008362	65.1	1.402092	91.89999	0.7539163	47.95	0.681867	44.8	1.128233	78.27499	NOT1; CDC39; NOT1H;	NM_01628	CNOT1
200800_s_at	0.552076	797.4251	0.73919	1241.45	1.47371	2187.9	1.8291482	2966.175	1.349963	1943.9	0.890294	1486.95	HSP72; HSPA1; HSPA1B	NM_00534	HSPA1A
200717_x_at	1.252602	12811.18	0.808283	8387.725	1.14416	11744.4	1.0587822	10795.2	0.818293	8332.899	0.56231	5750.675	humL7-1; MGC117326	NM_00097	RPL7
200710_at	0.932504	542.025	1.378792	816.725	1.019948	591.55	1.5961715	934.925	0.567246	355.425	0.699819	422.9	ACAD6; LCACD; VLCAD	NM_00001	ACADVL
200609_s_at	0.96983	4313.55	0.968503	4274.625	1.011947	4488.85	1.2142303	5384.025	0.739425	3278.55	1.021584	4512.125	AIP1; NORI-1	NM_01749	WDR1
200600_at	0.643613	1189.725	0.638843	1198.075	1.33826	2446.575	1.2267902	2334.225	1.352204	2538.625	0.992815	1838.075	MSN	NM_00244	MSN
200094_s_at	1.851767	2189.475	0.912872	1107.55	0.927945	1102.55	1.1084036	1329.775	0.950032	1129.125	0.802333	972	EEF2	AI004246	
200032_s_at	1.08163	13607.08	0.680081	8880.125	1.224495	15577.15	1.1162739	14009.05	0.948737	11911.93	0.52046	6676.675	FLJ27456; MGC15545;	NM_00066	RPL9
200031_s_at	1.226582	13174.75	0.721076	7927.15	1.217978	13153.7	1.1948081	12823.25	0.787997	8460.649	0.362865	4028.025	RPS11	NM_00101	RPS11
200025_s_at	1.292347	10216.85	0.77506	6329.45	1.199224	9490.55	1.1320907	9028.025	0.895097	7060	0.631393	5106.45	RPL27	NM_00098	RPL27
200019_s_at	1.220815	8723.249	0.738831	5270	1.159944	8271.625	1.1707351	8344.425	0.834131	5964.55	0.492239	3675.375	FAU1; RPS30; FLJ2298	NM_00199	FAU
200017_at	1.253313	6782.5	0.604856	3278.65	1.356304	7318.375	1.2352219	6709.6	0.910849	4890.7	0.338833	1916.8	CEP80; UBA80; UBCEP1	NM_00295	RPS27A
200016_x_at	1.040592	10494.77	0.879021	8953.525	1.014655	10250.78	1.171798	11831.13	0.946545	9531.225	0.968856	9834.8	HNRNPA1; MGC102835	NM_00213	HNRPA1
1563458_at	1.430255	28.525	0.524006	10.25	1.012795	19.6	1.0053003	20.225	1.05559	20	1.277522	27.875	PARVA	AL832682	
1561539_at	1.132204	18.7	0.882129	15.675	1.597429	27	0.8646262	14.225	0.400575	7.625	1.369085	22.2		BC041984	
1560562_a_at	1.699825	28.475	1.292111	21.25	0.996955	17.7	0.13589138	2.45	0.735796	21.15	0.442674	10.15	MGC48625	AK026366	
1559530_at	0.667549	42.975	1.029929	69.125	0.922439	62.3	1.071154	69.07501	1.646473	105.525	1.086063	75.125	BLOS3	BC040703	
1558943_x_at	1.097766	147.325	1.768986	238.975	0.671376	88.875	0.73144394	95.95	1.005582	131.025	1.229093	161.6	LOC91661	BU686397	

208758_at	2q35	2	3.5.4.10	5-aminoimidazole-4-carboxamide ribonucleotide formyltransf	5-aminoimic	AICA-ribosiduria due to	GeneID:471	GO:6139(nucleobase, nu		GO:3937(IN	NM_00404	Hs.90280	17353931; protein-coding
208752_x_at	12q21.1	12		nucleosome assembly protein 1-like 1								Hs.419776	
208714_at	11q13	11		NADH dehydrogenase (ubiquinone) flavoprotein 1, 51kDa	51kDa subunit of NADH dehydrogen		GI:5138912					Hs.7744	
208485_x_at	2q33-q34	2		CASP8 and FADD-like apoptosis regulator	CASP8 and FADD-like apoptosis regu		GeneID:883	GO:6916(anti-apoptosis		GO:30693(	NM_00387	Hs.390736	17106251; protein-coding
208319_s_at	Xp11.2	23		RNA binding motif (RNP1, RRM) protein 3	RNA binding motif protein 3 isoform		GeneID:593	GO:6396(RNA processin		GO:3723(R	NM_00101	Hs.301404	16552754; protein-coding
207891_s_at	xq28	23		three prime repair exonuclease 2	three prime repair exonuclease 2 iso		GeneID:11219; LocusID:11219; MIM:300370; GI:11120680					Hs.170835	
207850_at	4q21	4		chemokine (C-X-C motif) ligand 3	chemokine (C-X-C motif) ligand 3		GeneID:292	GO:7186(G	GO:5576(e	GO:8009(cl	NM_00209	Hs.89690	16356540; protein-coding
207777_s_at	2q37.1	2		SP140 nuclear body protein	SP140 nuclear body protein isoform		GeneID:112	GO:6952(de	GO:5635(nu	GO:3677(D	NM_00100	Hs.632549	12477932; protein-coding
207618_s_at	2q33	2		BCS1-like (yeast)	BCS1-like	Bjornstad syndrome; GR	GeneID:617	GO:15980(	GO:16021(i	GO:5524(A	NM_00432	Hs.471401	17314340; protein-coding
207505_at	4q13.1-q21	4		protein kinase, cGMP-dependent, type II	protein kinase, cGMP-dependent, typ		GeneID:559	GO:6468(protein amino		GO:5524(A	NM_00625	Hs.570833	15555551; protein-coding
206928_at	1q44	1		zinc finger protein 124	zinc finger protein 124		GeneID:767	GO:6355(re	GO:5622(in	GO:3677(D	NM_00343	Hs.655205	16710414; protein-coding
206760_s_at	19p13.3	19		Fc fragment of IgE, low affinity II, receptor for (CD23)	Fc fragment of IgE, low affinity II, rec		GeneID:220	heterophilic	GO:5887(in	GO:19863(	NM_00200	Hs.465778	16899715; protein-coding
206687_s_at	12p13	12	3.1.3.48	protein tyrosine phosphatase, non-receptor type 6	protein tyrosine phosphatase, non-re		GeneID:577	GO:7186(G	GO:5575(ce	GO:16787(	NM_00283	Hs.63489	17404032; protein-coding
206589_at	1p22	1		growth factor independent 1	growth fact	Neutropenia, nonimmun	GeneID:267	GO:83(G1/S	GO:5622(in	GO:3677(D	NM_00526	Hs.73172	17207994; protein-coding
206584_at	8q21.11	8		lymphocyte antigen 96	MD-2 protein		GeneID:236	GO:7166(ce	GO:5886(pl	GO:15026(	NM_01536	Hs.660766	16940155; protein-coding
206555_s_at	16p12.2	16		THUMP domain containing 1	THUMP domain containing 1		GeneID:55623; HGNC:23807; HPRD:HPRD_0861				NM_01773	Hs.460232	17081983; protein-coding
206544_x_at	9p22.3	9		SWI/SNF related, matrix associated, actin dependent regulat	SWI/SNF-related matrix-associated a		GeneID:659	GO:6357(re	GO:5654(nu	GO:5524(A	NM_00307	Hs.298990	17081983; protein-coding
206521_s_at	14q31.1	14		general transcription factor IIA, 1, 19/37kDa	TFIIA alpha, p55 isoform 1		GeneID:295	GO:6355(re	GO:5634(nu	GO:16251(	NM_01585	Hs.592334	17081983; protein-coding
206332_s_at	1q22	1		interferon, gamma-inducible protein 16	interferon, gamma-inducible protein		GeneID:342	GO:42771(	GO:5730(nu	GO:3677(D	NM_00553	Hs.380250	17339605; protein-coding
205849_s_at	8q22	8	1.10.2.2	ubiquinol-cytochrome c reductase binding protein	ubiquinol-cy	Mitochondrial complex III	GeneID:738	GO:9060(ae	GO:16020(	GO:16491(	NM_00629	Hs.131255	16189514; protein-coding
205804_s_at	1q32.3-q41	1		TRAF3 interacting protein 3	TRAF3-interacting JNK-activating mo		GeneID:80342; HGNC:3(		GO:16021(i	GO:16301(	NM_02522	Hs.147434	17277885; protein-coding
205419_at	13q32.3	13		Epstein-Barr virus induced gene 2 (lymphocyte-specific G pro	EBV-induced G protein-coupled recep		RP11-461N	GO:7186(G	GO:5887(in	GO:45028(	NM_00495	Hs.784	16540462; protein-coding
205306_x_at	1q42-q44	1		kynurenine 3-monooxygenase (kynurenine 3-hydroxylase)								Hs.409081	
205298_s_at	6p22.1	6		zd24d07.s1 Soares fetal heart NbHH19W Homo sapiens cDNA clone IMAGE:341581 3', mRNA sequence.									
205269_at	5q33.1-qte	5		lymphocyte cytosolic protein 2 (SH2 domain containing leukocyte protein of 76kDa)								Hs.2488	
205207_at	7p21	7		interleukin 6 (interferon, beta 2)	interleukin (	Kaposi sarcoma, suscep	GeneID:356	GO:6953(ac	GO:5576(ex	GO:5125(cy	NM_00060	Hs.654458	17472849; protein-coding
205191_at	Xp11.4-p11	23		retinitis pigmentosa 2 (X-linked recessive)	XRP2 protei	Retinitis pigmentosa-2;	GeneID:610	GO:6241(C	GO:16020(	GO:5524(A	NM_00691	Hs.44766	17093403; protein-coding
205077_s_at	2p21-p16	2		phosphatidylinositol glycan anchor biosynthesis, class F	phosphatidylinositol glycan, class F is		GeneID:528	GO:6506(G	GO:5783(er	GO:4307(e	NM_00264	Hs.468415	17028267; protein-coding
205050_s_at	22q13.33	22		mitogen-activated protein kinase 8 interacting protein 2	mitogen-activated protein kinase 8 in		GeneID:235	GO:45768(	GO:5737(cy	GO:5078(M	NM_01232	Hs.558180	16456539; protein-coding
205042_at	9p13.3	9	2.7.1.60	glucosamine (UDP-N-acetyl)-2-epimerase/N-acetylmannosan	UDP-N-acet	Inclusion body myopathy	GeneID:100	GO:6054(N	GO:5737(cy	GO:5524(A	NM_00547	Hs.5920	17164266; protein-coding
204972_at	12q24.2	12	2.7.7.-	2'-5'-oligoadenylate synthetase 2, 69/71kDa	2'-5'-oligoadenylate synthetase 2 iso		GeneID:493	GO:6401(R	GO:16020(	GO:1730(2	NM_00103	Hs.414332	16541075; protein-coding
204956_at	9p21	9	2.4.2.28	methylthioadenosine phosphorylase	5'-methylthioadenosine phosphoryla		GeneID:450	GO:6139(nucleobase, nu		GO:17061(	NM_00245	Hs.193268	16778103; protein-coding
204882_at	2p13.3	2											
204861_s_at	5q13.1	5		NLR family, apoptosis inhibitory protein	NLR family, apoptosis inhibitory prot		GeneID:467	GO:6916(ar	GO:5622(in	GO:5524(A	NM_00453	Hs.654500	15372022; protein-coding
204794_at	2q11	2	3.1.3.48	dual specificity phosphatase 2	dual specificity phosphatase 2		GeneID:184	GO:188(ina	GO:5634(nu	GO:17017(	NM_00441	Hs.1183	16288922; protein-coding
204599_s_at	16p13.3	16		mitochondrial ribosomal protein L28	mitochondrial ribosomal protein L28		GeneID:105	GO:6412(tr	GO:5761(m	GO:3735(st	NM_00642	Hs.513230	15616553; protein-coding
204528_s_at	12q21.2	12		nucleosome assembly protein 1-like 1	nucleosome assembly protein 1-like		GeneID:467	GO:6260(D	GO:5678(ch	GO:5515(pr	NM_00453	Hs.524599	17081983; protein-coding
204520_x_at	22q13.33	22		bromodomain containing 1	bromodomain containing protein 1		GeneID:237	GO:8150(bi	GO:5634(nu	GO:46872(	NM_01457	Hs.127950	17081983; protein-coding
204517_at	5q23.2	5		601656143R1 NIH_MGC_66 Homo sapiens cDNA clone IMAGE:3855754 3', mRNA sequence.									
204490_s_at	11p13	11		CD44 molecule (Indian blood group)	CD44 antig	Blood group, Indian syst	GeneID:960	GO:7155(ce	GO:5575(ce	GO:5518(co	NM_00061	Hs.502328	17343740; protein-coding
204224_s_at	14q22.1-q2	14	3.5.4.16	GTP cyclohydrolase 1 (dopa-responsive dystonia)	GTP cyclohy	Dystonia, D conversion	GeneID:264	GO:6559(L-	GO:5737(cy	GO:3934(G	NM_00016	Hs.86724	16696853; protein-coding
204118_at	1q21.3-q22	1		CD48 molecule	CD48 antigen (B-cell membrane prot		GeneID:962	GO:6952(de	GO:5887(in	GO:48503(	NM_00177	Hs.243564	17222190; protein-coding
204042_at	13q12	13		WAS protein family, member 3	WAS protein family, member 3		RP11-496O	GO:30041(	GO:15629(	GO:3779(a	NM_00664	Hs.635221	15907837; protein-coding
204026_s_at	10q21-q22	10		ZW10 interactor	ZW10 interactor isoform b		GeneID:111	GO:7049(ce	GO:776(kin	GO:47485(	NM_00100	Hs.591363	16732327; protein-coding
203984_s_at	1p36.3-p36	1		caspase 9, apoptosis-related cysteine peptidase	caspase 9 isoform alpha cysteine pro		RP11-265F	GO:8632(ap	GO:5622(in	GO:30693(	NM_00122	Hs.329502	17299206; protein-coding
203922_s_at	xp21.1	23		cytochrome b-245, beta polypeptide (chronic granulomatous disease)								Hs.88974	
203796_s_at	12q24.13	12		B-cell CLL/lymphoma 7A								Hs.371758	
203778_at	4q22-q25	4	3.2.1.25	mannosidase, beta A, lysosomal	mannosidas	Mannosidosis, beta	GeneID:412	GO:5975(ca	GO:5764(ly	GO:4567(b	NM_00590	Hs.480415	16344560; protein-coding
203525_s_at	5q21-q22	5		adenomatosis polyposis coli								Hs.75081	
203516_at	20q11.2	20		syntrophin, alpha 1 (dystrophin-associated protein A1, 59kD	acidic alpha 1 syntrophin		GeneID:664	GO:6936(m	GO:5856(cy	GO:3779(a	NM_00309	Hs.31121	17081983; protein-coding
203341_at	2p22.2	2		CCAAT/enhancer binding protein zeta	CCAAT/enhancer binding protein zet		GeneID:101	GO:6355(re	GO:5634(nu	GO:3677(D	NM_00576	Hs.135406	17081983; protein-coding
203203_s_at	12q21.2	12		KRR1, small subunit (SSU) processome component, homoloc	HIV-1 rev binding protein 2		GeneID:111	GO:6364(rF	GO:5634(nu	GO:3723(R	NM_00704	Hs.645517	17081983; protein-coding
203178_at	15q21.1	15	2.1.4.1	glycine amidinotransferase (L-arginine:glycine amidinotransfe	L-arginine:g	AGAT deficiency	GeneID:262	GO:6601(cr	GO:5758(m	GO:15068(	NM_00148	Hs.75335	17101918; protein-coding
203067_at	11p13	11		pyruvate dehydrogenase complex, component X	pyruvate de	Lacticacidemia due to PI	GeneID:805	GO:8152(m	GO:5739(m	GO:8415(a	NM_00347	Hs.502315	16843025; protein-coding
203064_s_at	17q25	17		forkhead box K2	forkhead box K2		GeneID:360	GO:6357(re	GO:5634(nu	GO:3702(R	NM_00451	Hs.693612	16964243; protein-coding
203063_at	22q11.22	22	3.1.3.16	protein phosphatase 1F (PP2C domain containing)	protein phosphatase 1F		GeneID:964	GO:6915(ap	GO:8287(pr	GO:16787(	NM_01463	Hs.112728	16344560; protein-coding
203060_s_at	10q23-q24	10	2.7.7.4	3'-phosphoadenosine 5'-phosphosulfate synthase 2	3'-phospho	SEMD, Pakistani type	GeneID:906	GO:6139(nu	GO:5622(in	GO:5524(A	NM_00101	Hs.524491	17353931; protein-coding
202959_at	6p21	6		methylmalonyl Coenzyme A mutase								Hs.155212	
202869_at	12q24.1	12	2.7.7.-	2',5'-oligoadenylate synthetase 1, 40/46kDa	2',5'-oligoa	Diabetes mellitus, type	GeneID:493	GO:6955(in	GO:5737(cy	GO:5524(A	NM_00103	Hs.524760	17360168; protein-coding
202813_at	1q42.3	1		Tar (HIV-1) RNA binding protein 1	TAR RNA binding protein 1		RP5-827C2	GO:6396(R	GO:5634(nu	GO:3723(R	NM_00564	Hs.498115	16710414; protein-coding
202520_s_at	3p21.3	3		mutL homolog 1, colon cancer, nonpolyposis type 2 (E. coli)	MutL protei	Cafe-au-lait spots with c	GeneID:429	GO:7049(ce	GO:5634(nu	GO:5524(A	NM_00024	Hs.195364	17374836; protein-coding
202494_at	1p32	1	5.2.1.8	peptidylprolyl isomerase E (cyclophilin E)	peptidylprolyl isomerase E isoform 1		RP1-118J2	GO:8380(R	GO:5634(nu	GO:3723(R	NM_00611	Hs.524690	16710414; protein-coding
202447_at	8q21.3	8	1.3.1.34	2,4-dienoyl CoA reductase 1, mitochondrial	2,4-dienoyl CoA reductase 1 precurs		GeneID:166	GO:8152(m	GO:5739(m	GO:8670(2	NM_00135	Hs.492212	16421571; protein-coding
202347_s_at	4p14	4	6.3.2.19	huntingtin interacting protein 2	huntingtin interacting pr	intracellular	GeneID:309	GO:6464(pr	GO:5634(nu	GO:16874(	NM_00533	Hs.50308	17353931; protein-coding
202307_s_at	6p21.3	6		transporter 1, ATP-binding cassette, sub-family B (MDR/TAP	transporter	Bare lymphocyte syndro	DAQB-69D7	GO:6952(de	GO:5783(er	GO:5524(A	NM_00059	Hs.352018	17164240; protein-coding
202261_at	1q21	1		vacuolar protein sorting 72 homolog (S. cerevisiae)	transcription factor-like 1		GeneID:694	GO:16568(	GO:5634(nu	GO:3702(R	NM_00599	Hs.2430	16710414; protein-coding
202251_at	1q21.1	1		PRP3 pre-mRNA processing factor 3 homolog (S. cerevisiae)	PRP3 pre-m	Retinitis pigmentosa-18	GeneID:912	GO:8380(R	GO:5634(nu	GO:31202(	NM_00469	Hs.11776	17353931; protein-coding
202239_at	13q11	13	2.4.2.30	poly (ADP-ribose) polymerase family, member 4	poly (ADP-ribose) polymerase family		GeneID:143	GO:6281(D	GO:5622(in	GO:3677(D	NM_00643	Hs.591227	16344560; protein-coding
202157_s_at	10p13	10		CUG triplet repeat, RNA binding protein 2	CUG triplet repeat, RNA binding prot		GeneID:106	GO:6396(R	GO:5634(nu	GO:3723(R	NM_00102	Hs.309288	17383427; protein-coding
202156_s_at	10p13	10		CUG triplet repeat, RNA binding protein 2								Hs.211610	
202093_s_at	19q13.1	19		Paf1, RNA polymerase II associated factor, homolog (S. cere	Paf1, RNA polymerase II associated f		GeneID:54623; HGNC:25459; HPRD:HPRD_1014(				NM_01908	Hs.466714	16491129; protein-coding
202068_s_at	19p13.3	19		low density lipoprotein receptor (familial hypercholesterolem	low density	Hypercholesterolemia, fa	GeneID:394	GO:30299(	GO:5905(co	GO:5509(ca	NM_00052	Hs.213289	17328821; protein-coding
202042_at	5q31.3	5	6.1.1.21	histidyl-tRNA synthetase	histidyl-tRNA synthetase		GeneID:303	GO:6427(hi	GO:5737(cy	GO:5524(A	NM_00210	Hs.528050	15489334; protein-coding
202029_x_at	17q23-q25	17		ribosomal protein L38	ribosomal protein L38		GeneID:616	GO:6412(tr	GO:5842(cy	GO:3723(R	NM_00099	Hs.380953	17353931; protein-coding
201858_s_at	10q22.1	10		serglycin	serglycin precursor		GeneID:5552; HGNC:9361; HPRD:HPRD_01513; I				NM_00272	Hs.1908	16870619; protein-coding
201850_at	2p11.2	2		capping protein (actin filament), gelsolin-like	gelsolin-like capping protein		GeneID:822	GO:51016(	GO:8290(F-	GO:3779(a	NM_00174	Hs.516155	17081065; protein-coding
201849_at	10q26.3	10		BCL2/adenovirus E1B 19kDa interacting protein 3	BCL2/adenovirus E1B 19kD-interacti		GeneID:664	GO:6309(D	GO:5737(cy	GO:46982(	NM_00405	Hs.144873	17394490; protein-coding
201837_s_at	2pter-p25.	2		SPTF-associated factor 65 gamma	adenocarcinoma antigen ART1		GI:1106625	regulation c	nucleus	molecular function unkr		Hs.6232	
201830_s_at	10p15	10		neuroepithelial cell transforming gene 1	neuroepithelial cell transforming gen		GeneID:102	GO:35023(	GO:5575(ce	GO:5089(R	NM_00586	Hs.25155	16552434; protein-coding
201825_s_at	1q44	1		CGI-49 protein								Hs.238126	
201798_s_at	10q24	10		fer-1-like 3, myoferlin (C. elegans)	myoferlin isoform a		GeneID:265	GO:8015(ci	GO:16021(integral to m		NM_01345	Hs.655278	17081983; protein-coding
201780_s_at	3q25.1	3		ring finger protein 13	ring finger protein 13 isoform 1		GeneID:113	GO:16567(	GO:5634(nu	GO:46872(	NM_00728	Hs.12333	16166738; protein-coding
201768_s_at	5q23.1-q33	5		clathrin interactor 1	epsin 4	Schizophrenia, susceptib	GeneID:968	GO:6897(en	GO:5794(G	GO:8289(li	NM_01466	Hs.644000	17081983; protein-coding
201762_s_at	14q11.2	14		proteasome (prosome, macropain) activator subunit 2 (PA2	proteasome activator subunit 2		GeneID:572	GO:6955(in	GO:5829(cy	GO:8538(pr	NM_00281	Hs.434081	16189514; protein-coding
201752_s_at	10q24.2-q2	10		adducin 3 (gamma)								Hs.324470	
201683_x_at	14q11.2	14		chromosome 14 open reading frame 92								Hs.194035	
201641_at	19p13.2	19		bone marrow stromal cell antigen 2	bone marrow stromal cell antigen 2		GeneID:684	GO:42113(	GO:5887(in	GO:48503(	NM_00433	Hs.118110	16602701; protein-coding
201537_s_at	17q21	17	3.1.3.48	dual specificity phosphatase 3 (vaccinia virus phosphatase V	dual specificity phosphatase 3		GeneID:184	GO:6470(protein amino		GO:16787(	NM_00409	Hs.181046	16604064; protein-coding
201531_at	19q13.1	19		zinc finger protein 36, C3H type, homolog (mouse)	zinc finger protein 36, C3H type, hor		GeneID:753	GO:50779(	GO:5737(cy	GO:3677(D	NM_00340	Hs.534052	17369404; protein-coding
201520_s_at	4q13	4		G-rich RNA sequence binding factor 1								Hs.309763	
201508_at	17q12-q21	17		insulin-like growth factor binding protein 4	insulin-like growth factor binding pro		GeneID:348	GO:6259(D	GO:5576(ex	GO:5520(in	NM_00155	Hs.462998	17177834; protein-coding
201460_at	1q32	1		mitogen-activated protein kinase-activated protein kinase 2								Hs.75074	
201408_at	2p23	2		protein phosphatase 1, catalytic subunit, beta isoform								Hs.21537	
201403_s_at	1q23	1	2.5.1.18	microsomal glutathione S-transferase 3	microsomal glutathione S-transferase		RP11-466F	GO:6629(lip	GO:5783(er	GO:4364(gl	NM_00452	Hs.191734	16710414; protein-coding
201377_at	1q21.3	1		ubiquitin associated protein 2-like	ubiquitin associated protein 2-like		GeneID:9898; HGNC:29877; HPRD:HI			GO:5515(pr	NM_01484	Hs.490551	17081983; protein-coding
201359_at	11p15.2	11		coatomer protein complex, subunit beta 1	coatomer protein complex, subunit b		GeneID:131	GO:6888(El	GO:30126(	GO:5515(pr	NM_01645	Hs.339278	16713569; protein-coding
201198_s_at	2q37.1	2		proteasome (prosome, macropain) 26S subunit, non-ATPase, 1								Hs.3887	
201146_at	2q31	2		nuclear factor (erythroid-derived 2)-like 2	nuclear factor (erythroid-derived 2)-		GeneID:478	GO:6355(re	GO:5634(nu	GO:46983(	NM_00616	Hs.155396	17259171; protein-coding
201076_at	22q13.2-q1	22		NHP2 non-histone chromosome protein 2-like 1 (S. cerevisia	NHP2 non-histone chromosome prot		GeneID:480	GO:8380(R	GO:5730(nu	GO:3723(R	NM_00100	Hs.182255	16169070; protein-coding
201068_s_at	7q22.1-q22	7		proteasome (prosome, macropain) 26S subunit, ATPase, 2	proteasome 26S ATPase subunit 2		GeneID:570	GO:30163(	GO:5737(cy	GO:5524(A	NM_00280	Hs.437366	17353931; protein-coding
201060_x_at	9q34.1	9		tp32g06.x1 NCI_CGAP_Ut4 Homo sapiens cDNA clone IMAGE:2189530 3' similar to gb:M81635 ERYTHROCYTE BAND 7 INTEGRAL MEMBRANE PROTEIN (HUMAN);, mRNA sequence.									
200992_at	11p15.3	11		importin 7	hypothetical protein		GI:6807828; TrEMBL:O9NTE3					Hs.434408	
200963_x_at	2q11.2	2		ribosomal protein L31	ribosomal protein L31 isoform 1		GeneID:616	GO:6412(tr	GO:5842(cy	GO:3723(R	NM_00099	Hs.469473	17081983; protein-coding
200959_at	16p11.2	16		fusion (involved in t(12;16) in malignant liposarcoma)	fusion (involved in t(12;16) in malign		GeneID:252	GO:4(biolog	GO:8372(ce	GO:3677(D	NM_00101	Hs.513522	16230076; protein-coding
200933_x_at	Xq13.1	23		ribosomal protein S4, X-linked	ribosomal protein S4, X-linked X isofo		GeneID:619	GO:8283(ce	GO:5843(cy	GO:19843(	NM_00100	Hs.446628	15883184; protein-coding
200926_at	5q14.2	5		ribosomal protein S23	ribosomal protein S23		GeneID:622	GO:6412(tr	GO:5843(cy	GO:3723(R	NM_00102	Hs.527193	15883184; protein-coding
200912_s_at	3q28	3		eukaryotic translation initiation factor 4A, isoform 2	eukaryotic translation initiation facto		GeneID:197	GO:6446(re	GO:16281(	GO:5524(A	NM_00196	Hs.654367	16567544; protein-coding
200861_at	16q21	16		CCR4-NOT transcription complex, subunit 1	CCR4-NOT transcription complex, sul		GeneID:23019; HGNC:7877; HPRD:HI			GO:5515(pr	NM_01628	Hs.460923	17353931; protein-coding
200800_s_at	6p21.3	6		heat shock 70kDa protein 1A	heat shock 70kDa protein 1A		DAQB-147D	GO:6457(protein foldinc		GO:5524(A	NM_00534	Hs.520028	17332370; protein-coding
200717_x_at	8q21.11	8		ribosomal protein L7	ribosomal protein L7		GeneID:612	GO:6412(tr	GO:5842(cy	GO:3723(R	NM_00097	Hs.571841	17258209; protein-coding
200710_at	17p13-p11	17		acyl-Coenzyme A dehydrogenase, very long chain	acyl-Coenzy	VLCAD deficiency	GeneID:37;	GO:6118(el	GO:5739(m	GO:50660(	NM_00001	Hs.437178	16464760; protein-coding
200609_s_at	4p16.1	4		WD repeat domain 1	WD repeat-containing protein 1 isofo		GeneID:994	GO:7605(se	GO:5856(cy	GO:3779(a	NM_00511	Hs.128548	15629458; protein-coding
200600_at	Xq11.2-q12	23		moesin	moesin		GeneID:447	GO:6928(ce	GO:16324(	GO:8092(cy	NM_00244	Hs.87752	17237445; protein-coding
200094_s_at	19pter-q12	19		Transcribed sequence with strong similarity to protein sp:P13639 (H.sapiens) EF2_HUMAN Elongation factor 2								Hs.501443	
200032_s_at	4p13	4		ribosomal protein L9	ribosomal protein L9		GeneID:613	GO:6412(tr	GO:5622(in	GO:3723(R	NM_00066	Hs.412370	16341674; protein-coding
200031_s_at	19q13.3	19		ribosomal protein S11	ribosomal protein S11		GeneID:620	GO:6412(tr	GO:5842(cy	GO:19843(	NM_00101	Hs.433529	15883184; protein-coding
200025_s_at	17q21.1-q2	17		ribosomal protein L27	ribosomal protein L27		GeneID:615	GO:6412(tr	GO:5622(in	GO:3735(st	NM_00098	Hs.514196	15635413; protein-coding
200019_s_at	11q13	11		Finkel-Biskis-Reilly murine sarcoma virus (FBR-MuSV) ubiquit	ubiquitin-like protein fubi and ribosor		GeneID:219	GO:8150(bi	GO:5575(ce	GO:3723(R	NM_00199	Hs.387208	15883184; protein-coding
200017_at	2p16	2		ribosomal protein S27a	ubiquitin and ribosomal protein S27a		GeneID:623	GO:6464(pr	GO:5843(cy	GO:46872(	NM_00295	Hs.546292	16189514; protein-coding
200016_x_at	12q13.1	12		heterogeneous nuclear ribonucleoprotein A1	heterogeneous nuclear ribonucleopro		GeneID:317	GO:6405(R	GO:5737(cy	GO:3676(n	NM_00213	Hs.693600	17229681; protein-coding
1563458_at	11p15.3	11		parvin, alpha								Hs.44077	
1561539_at		0		Clone IMAGE:5303543, mRNA								Hs.434775	
1560562_a_at	19q13.42	19		hypothetical protein MGC48625								Hs.20506	
1559530_at	19q13.32	19		CDNA FLJ26676 fis, clone MPG03726								Hs.528492	
1558943_x_at	19q13.42	19		hypothetical protein BC001610								Hs.190394	

1557905_s_at	0.462194	227.2	0.336608	164.225	1.747478	999.775	2.746995	1702.1	1.330609	662.075	1.210527	668.35	CD44	AL552534		11p13	11		CD44 antigen (homing function and Indian blood group system)						Hs.306278	
1556162_at	0.818046	39.8	0.667273	32.45	1.125879	53.5	0.82342577	40.925	0.920719	44.175	1.418781	69.675	V8; EWI-3; MGC117164	AK097809	IGSF3	1p13	1		immunoglobulin superfamily, member 3	immunoglobulin superfamily, membe	RP4-781D12.1				NM_00100	Hs.171057	14702039; protein-coding
1555844_s_at	0.559473	1655.85	1.029472	3049.375	0.754418	2285.125	1.2920656	4007.45	1.069092	3211.6	1.127761	3369.75	HNRPM	AL713781		19p13.3-p1	19		heterogeneous nuclear ribonucleoprotein M	hypothetical protein	GI:19584535; GOA:O8TCM5; TrEMBL:O8TCM5					Hs.385766	
1554719_at	1.108954	28.95	0.790191	22.25	1.01857	26.025	1.3996282	38.1	0.713423	18.375	1.489687	41.4	CI-42KD; MGC5103	BC031332	NDUFA10	2q37.3	2	1.6.99.3	NADH dehydrogenase (ubiquinone) 1 alpha subcomplex, 10,	NADH dehydrogenase (ubiquinone) 1	GeneID:470	GO:6091(a	GO:5747(m	GO:5524(A	NM_00454	Hs.277677	16806233; protein-coding
1554519_at	0.034874	99.35001	0.037985	109.775	1.173641	3655.95	0.8659057	2592.375	1.682492	4751.4	1.133938	3192.525	LAB7; CD28LG; CD28LG	BC042665	CD80	3q13.3-q21	3		CD80 molecule	CD80 antigen precursor	GeneID:941	GO:42110(	GO:16021(i	GO:15026(	NM_00519	Hs.838	17201120; protein-coding
1554047_at	0.697849	24.7	0.874693	31.75	0.865868	29.9	0.7056306	25.85	1.085657	36.75	1.976232	67.5	APACD	BC024223	TXNDC9	2q11.2	2		thioredoxin domain containing 9	ATP binding protein associated with	GeneID:101	GO:8150(bi	GO:5575(ce	GO:3674(m	NM_00578	Hs.536122	16169070; protein-coding
1553768_a_at	0.543595	63.35	0.920909	114.275	1.133554	113.95	1.1402898	124.9	0.89363	115.075	0.585403	95.55	MGC46341; dJ94G16.1	NM_17367	DCBLD1	6q22.2	6		discoidin, CUB and LCCL domain containing 1	discoidin, CUB and LCCL domain cont	RP1-94G16	GO:7155(ce	GO:16021(integral to m		NM_17367	Hs.658304	15489334; protein-coding
1552942_at	1.07773	40	0.939303	35.425	1.45035	53.775	0.6767355	25.45	0.911276	34.3	1.15935	43.075	FLJ30430	NM_153009		1q42.2	1		hypothetical protein FLJ30430	hypothetical protein FLJ30430	GeneID:149373; LocusID:149373; GI:23308531					Hs.350904	
