# Supplementary material for: Ribosomal protein mRNAs are translationally-regulated during human dendritic cells activation by LPS
Source: Immunome Res. 2009 Nov 27;5:5. doi: 10.1186/1745-7580-5-5 (PMC2788525; doi:10.1186/1745-7580-5-5)

A)

Fold change Array

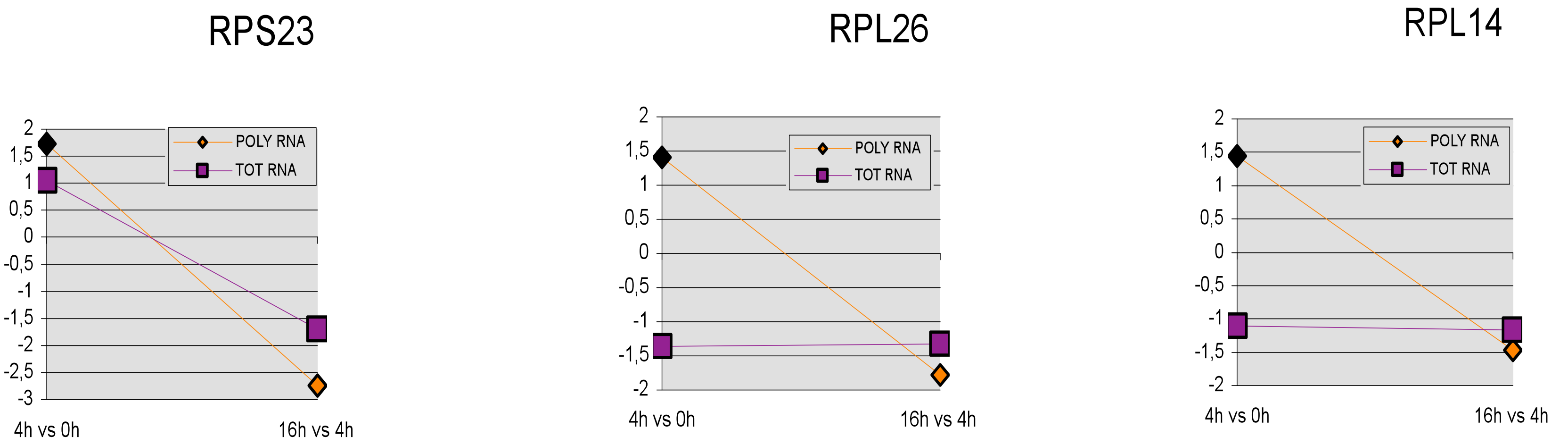

Fold change qPCR

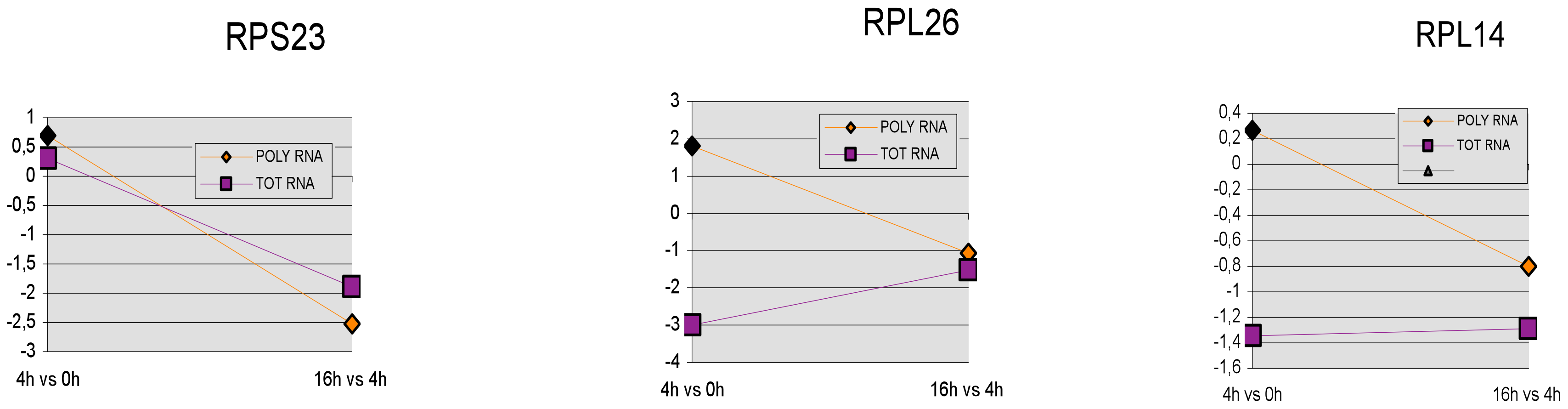

B)

Fold change Array

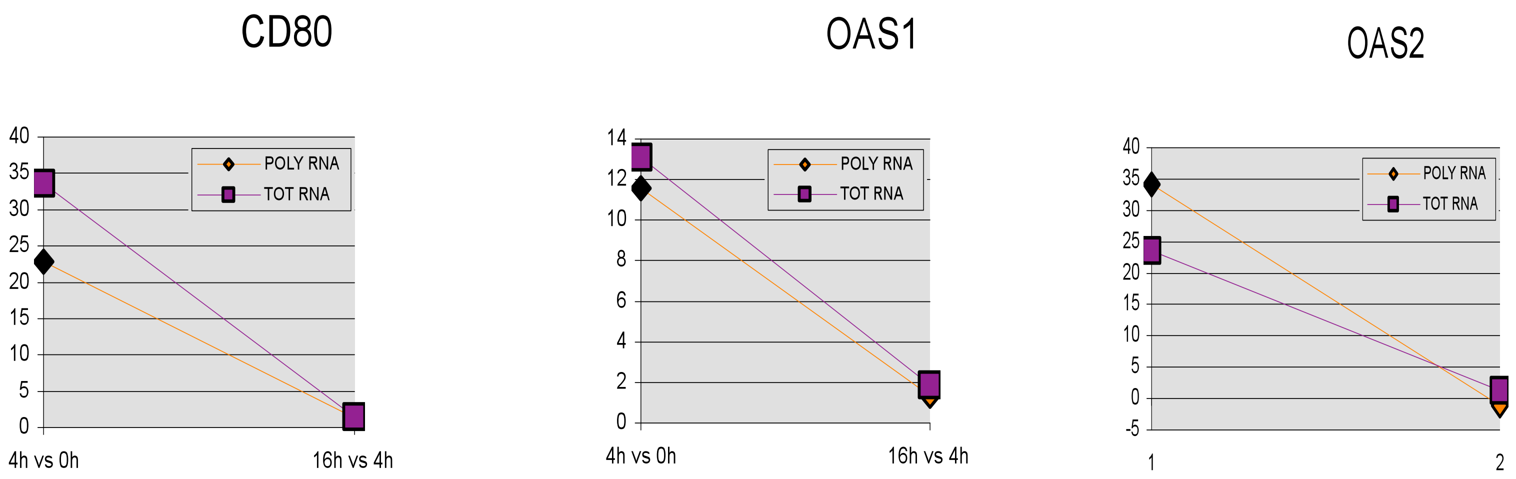

Fold change qPCR

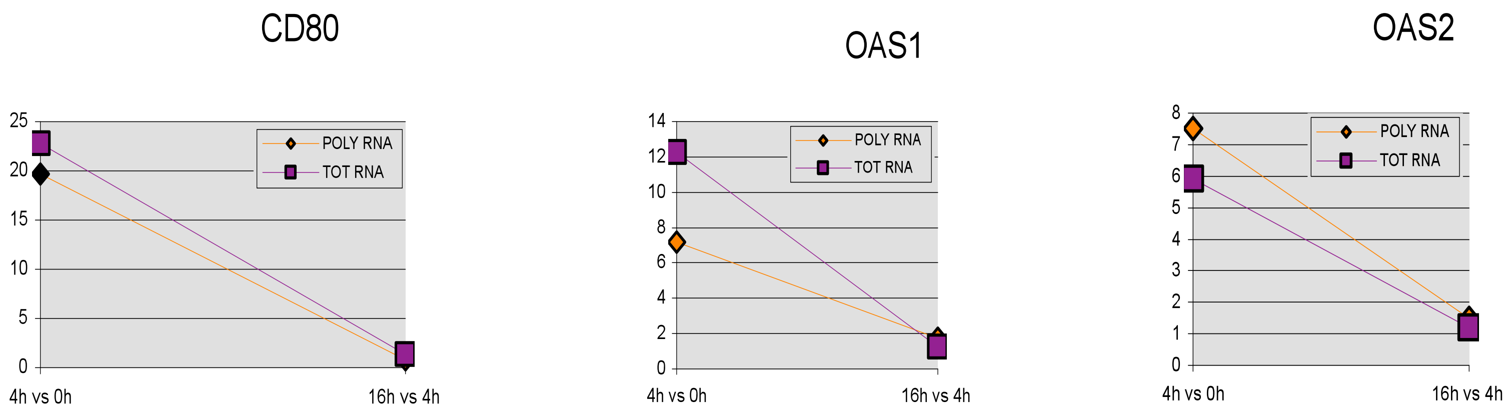

C)

Fold change Array

CASP9

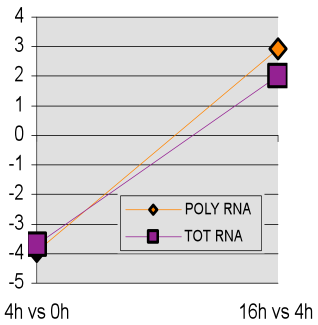

HLA-F

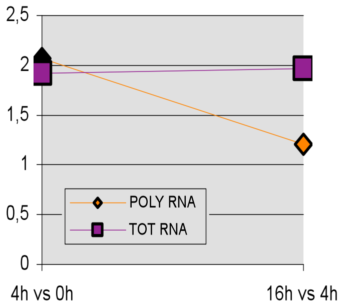

INDO

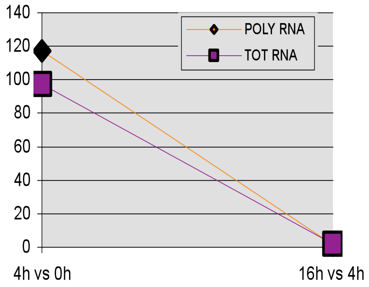

Fold change qPCR

CASP9

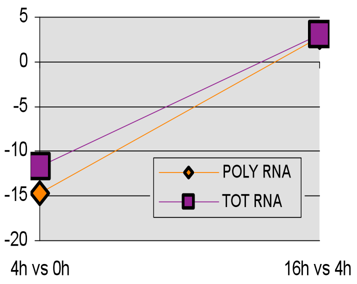

HLA-F

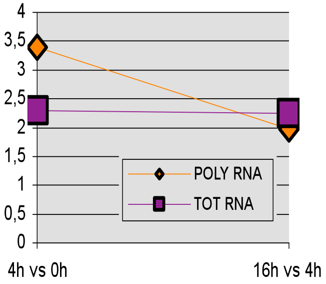

INDO

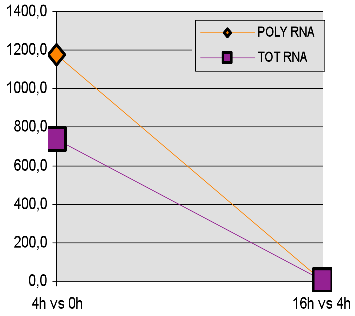

D)

Fold change Array

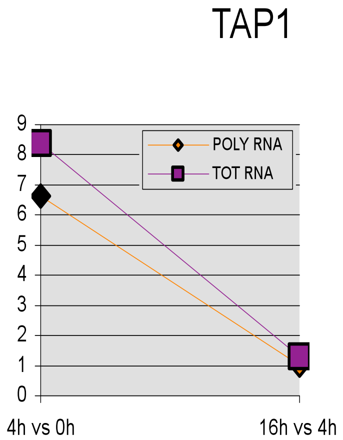

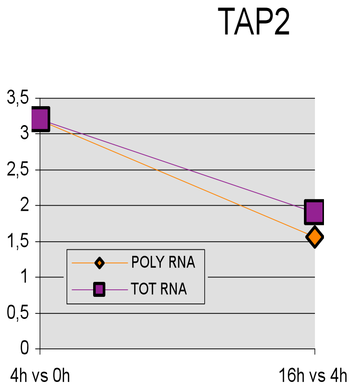

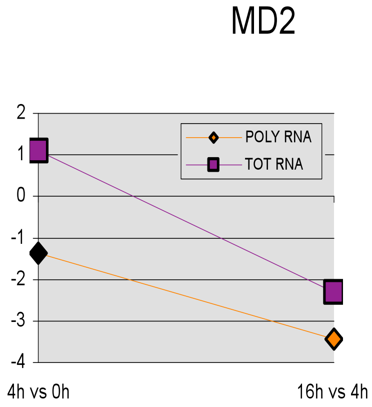

Fold change qPCR

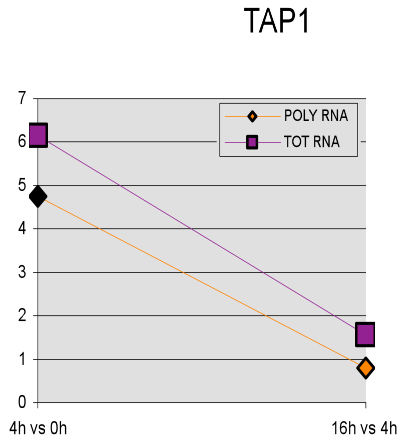

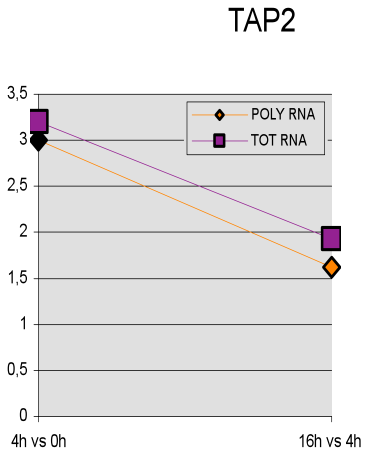

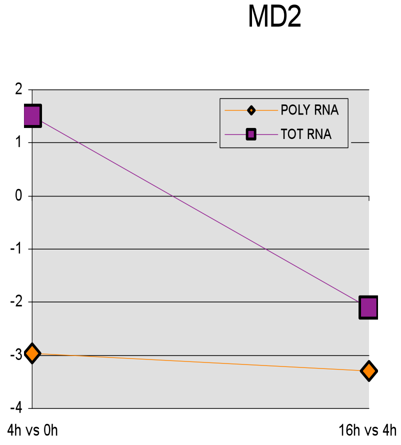

E)

Fold change Array

eIF4B

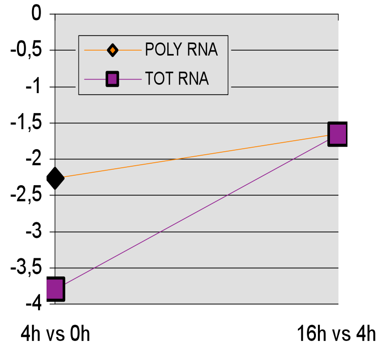

IL-6

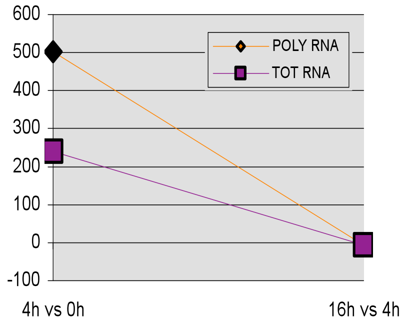

Ceppi et al, Figure S3E

Fold change qPCR

eIF4B

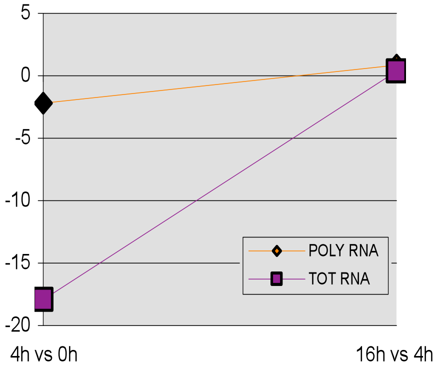

IL6

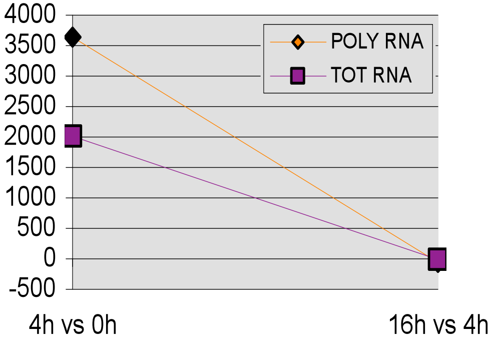

Supplement: Additional file 5 — (A-E) shows the validation of the array data by real time qPCR using Total and Polysomal RNA populations, and is related to Table 2. [file 1745-7580-5-5-S5.PDF]
