# Supplementary material for: Ribosomal protein mRNAs are translationally-regulated during human dendritic cells activation by LPS
Source: Immunome Res. 2009 Nov 27;5:5. doi: 10.1186/1745-7580-5-5 (PMC2788525; doi:10.1186/1745-7580-5-5)
Supplement: Additional file 6 — (A-D) shows correlations between Array and PCR Data after Spearman correlations. [file 1745-7580-5-5-S6.PDF]

Correlation between Array and PCR Data

RNA Type	Comparison			Spearman Correlation	p-value
Poly	PCR4v0	and	A4v0	0.97363	<.0001
Poly	PCR16v4	and	A16v4	0.88132	<.0001
Total	PCR4v0	and	A4v0	0.98681	<.0001
Total	PCR16v4	and	A16v4	0.95165	<.0001

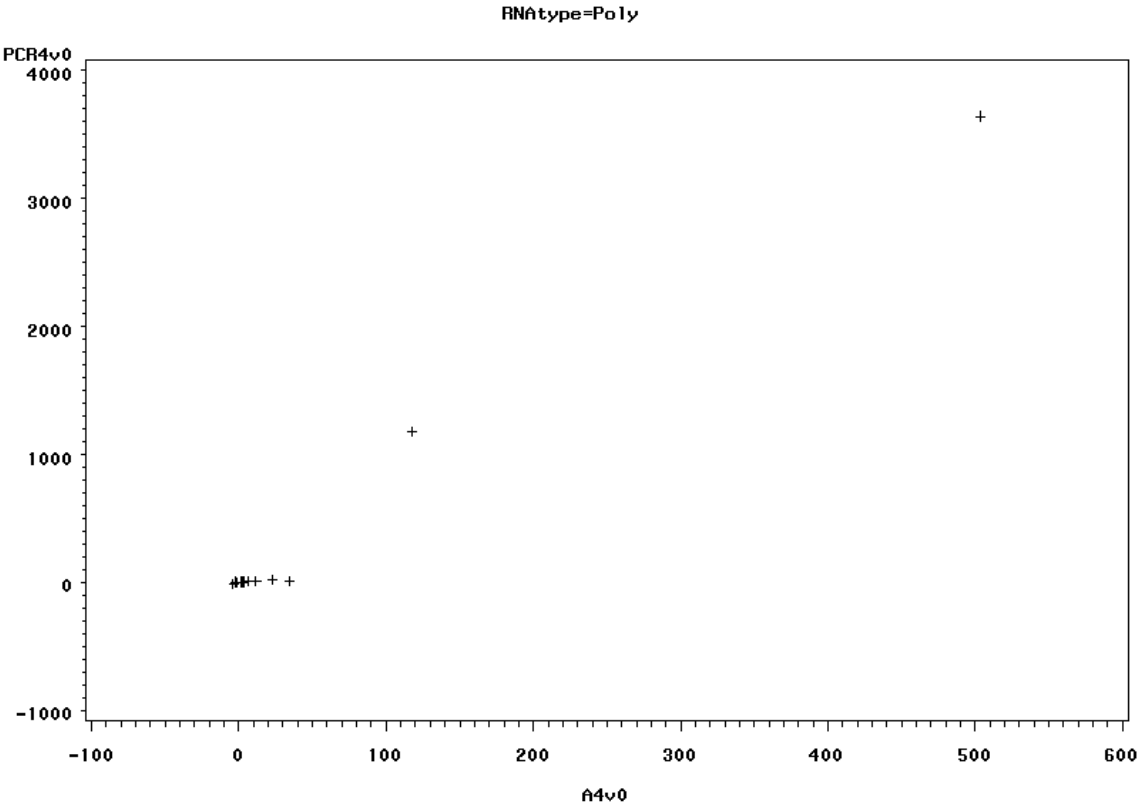

RNAtype=Poly

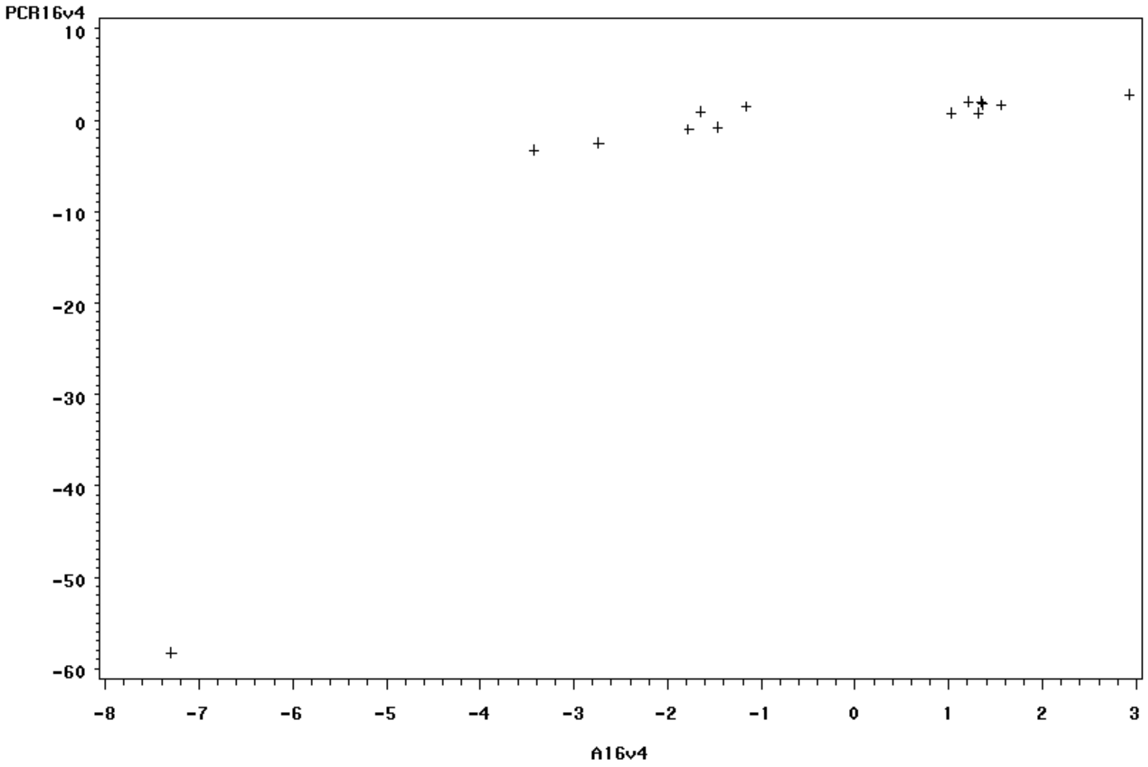

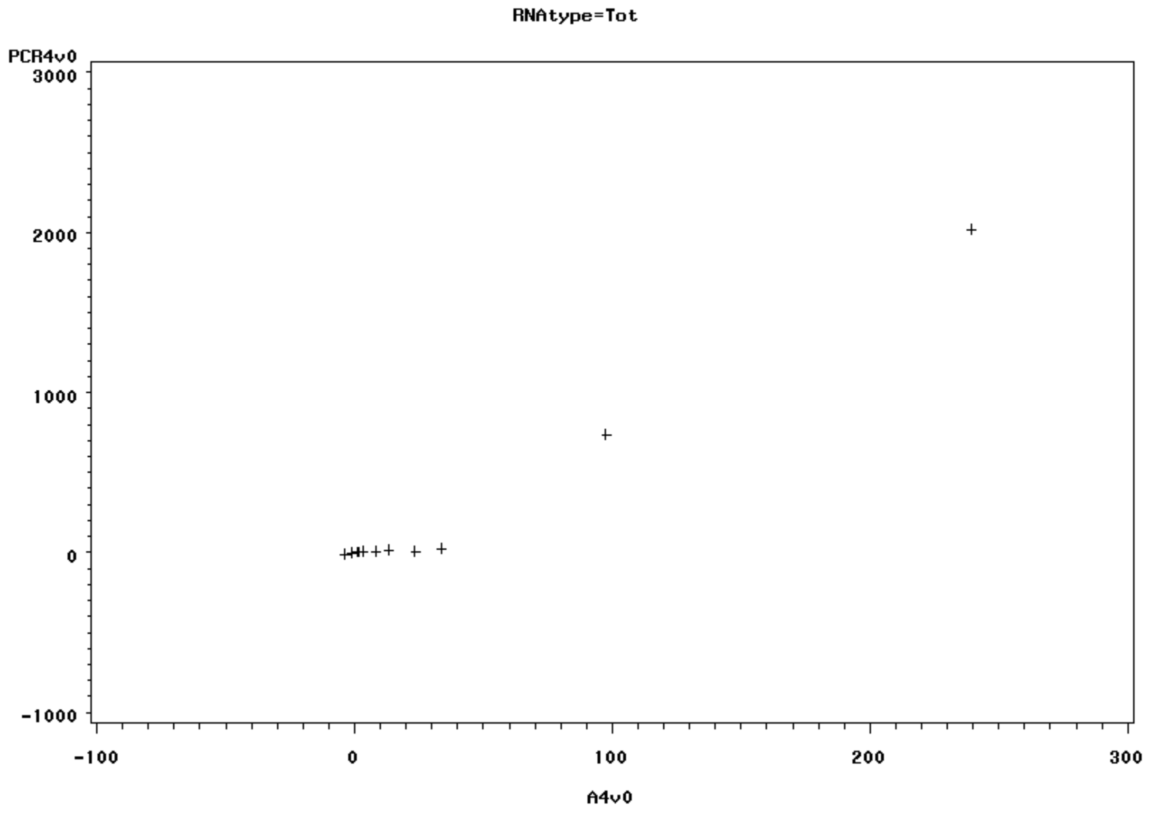
RNAtype=Tot
PCR4v0
3000
2000
1000
0
-1000
-100
0
100
200
300
A4v0

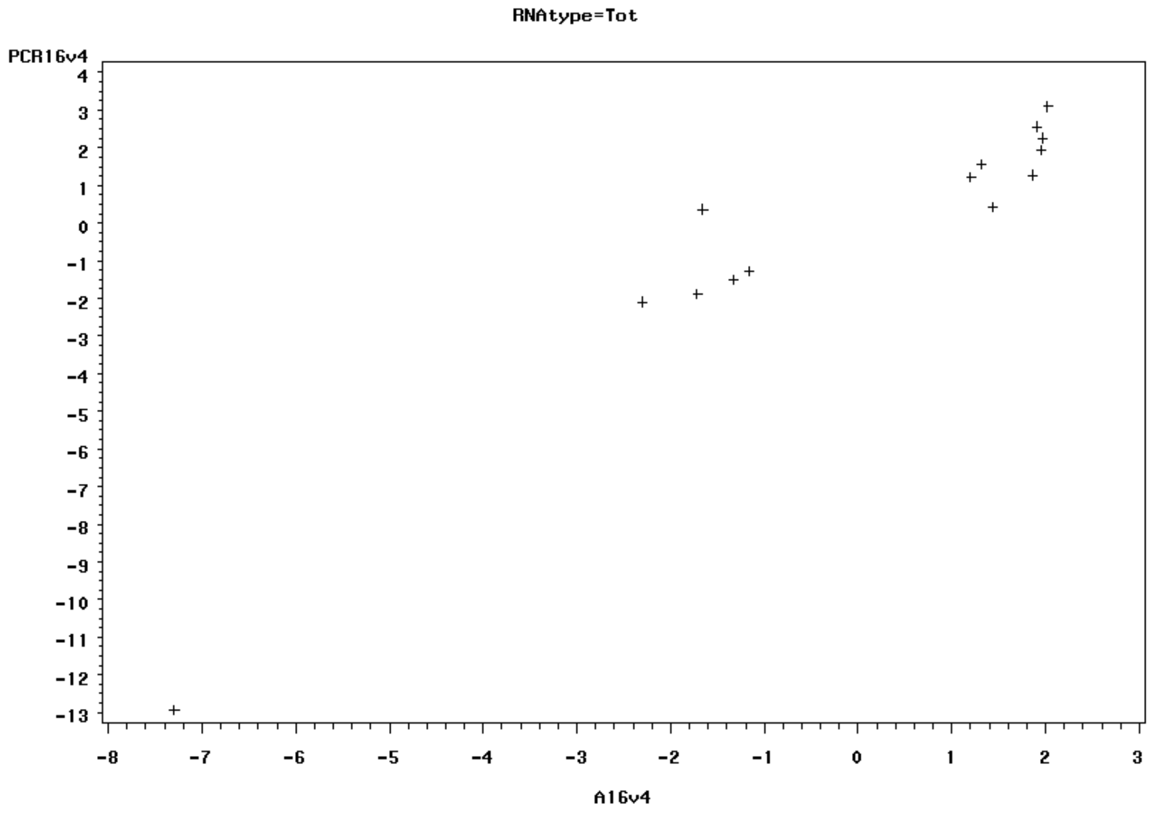
RNAtype=Tot
PCR16v4
4
3
2
1
0
-1
-2
-3
-4
-5
-6
-7
-8
-9
-10
-11
-12
-13
-8
-7
-6
-5
-4
-3
-2
-1
0
1
2
3
A16v4
