# Supplementary material for: Ribosomal protein mRNAs are translationally-regulated during human dendritic cells activation by LPS
Source: Immunome Res. 2009 Nov 27;5:5. doi: 10.1186/1745-7580-5-5 (PMC2788525; doi:10.1186/1745-7580-5-5)
Supplement: Additional file 7 — Shows the sequences of all employed primers for quantitative real-time PCR. [file 1745-7580-5-5-S7.PDF]

Primer sequences (5’to 3’ direction) employed for quantitative real-time RT-PCR

hRPS23 left	cccactttccgtaggatcaa
hRPS23 right	tgagaccagtgttgctggag
hIL-6 left	cacacagacagccactcacc
hIL-6 right	ttttctgccagtgcctcttt
hCD80 left	gcacatctcatggcagctaa
hCD80 right	cacaggagcaaggtttgtga
hCD48 left	cctgtacccaagcctgtcat
hCD48 right	ctgttctggagctcctttgg
hOAS1 left	acaggcagaagaggactgga
hOAS1 right	ggatcaagagtcccacctga
hOAS2 left	gcaccaataccccctacctt
hOAS2 right	tgcgccattctttgttagtg
hCASP9 left	ctagtttgcccacacccagt
hCASP9 right	gggactgcaggtcttcagag
hHLA-F left	ttgttgtccttggagctgtg
hHLA-F right	gtcccacacaaggaagctgt
hINDO left	cctgaggagctaccatctgc
hINDO right	tcagtgcctccagttccttt
hTAP1 left	gcagctcatggagaaaaagg
hTAP1 right	gaaaagggagggagatggag
hTAP2 left	aggaggctgcttcacctaca
hTAP2 right	tgagttcagctcccctgtct
hCXCR4 left	cgtggaacgtttttcctgtt
hCXCR4 right	ggtgctgaaatcaacccact
hRPL26 left	ggaaaaggctaatggcacaa
hRPL26 right	tcctttcctacttggcgaga
hRPL14 left	gtgcatgcagctcactgatt
hRPL14 right	ttcaatcttcttggcccatc
human MD2 left	ccgaggatctgatgacgatt
human MD-2 right	gggctcccagaaatagcttc
human eIF4B left	agagcagcagtcccctacaa
human eIF4B right	agttcccagtttggcctttt
human GAPDH left	ggcctccaaggagtaagacc
human GAPDH right	aggggtctacatggcaactg
